# Case fatality rate considering the lag time from the onset of COVID-19 infection to related death from 2020 to 2022 in Japan

**DOI:** 10.1016/j.ijregi.2023.04.013

**Published:** 2023-05-01

**Authors:** Shuko Nojiri, Yuta Kawakami, Daisuke Nakamoto, Manabu Kuroki, Yuji Nishizaki

**Affiliations:** a Clinical Translational Science, Juntendo University Graduate School of Medicine, Tokyo, Japan; b Medical Technology Innovation Centre, Juntendo University, Tokyo, Japan; c Faculty of Engineering, Yokohama National University, Kanagawa, Japan; d Department of Medical Education, Juntendo University Graduate School of Medicine, Tokyo, Japan

**Keywords:** COVID-19, SARS-CoV-2, Case fatality rate, Ecological study, Japan, Epidemiology, Lag time

## Abstract

**Importance:**

On an ecological scale, the lag time between coronavirus disease 2019 (COVID-19) infection and related fatality has varied between epidemic waves and prefectures in Japan. The variability in lag time across areas of Japan during the seven distinct waves can help derive a more appropriate estimation of the weekly confirmed case fatality rate (CFR) of COVID-19.

**Objective:**

To estimate the 7-day moving average CFR across area block levels in Japan from February 2020 to July 2022 using the lag time between COVID-19 infection and related fatality.

**Main outcomes and measures:**

The 7-day moving average CFR of COVID-19 for area blocks in Japan considering the lag time between infection and death (total and subgroup analysis of elderly).

**Results:**

Lag time was found to vary substantially among prefectures in Japan from the first wave to the seventh wave of the COVID-19 epidemic. The estimated 7-day moving average CFR based on the lag time reflects the Japanese COVID-19 pandemic and related policy interventions (e.g. vaccination of elderly people) rather than other standard CFR estimations.

**Conclusions and relevance:**

The variation in estimated lag time across prefectures in Japan for different epidemic waves indicates that it is inadequate to use the clinical results of the period from the start of infection to death for evaluation of the ecological scale of the CFR. Moreover, the lag time between infection and related fatality was found to be either shorter or longer than the clinically reported period. This revealed that preliminary reports of CFR may be overestimated or underestimated, even if they consider the lag based on clinical reports.

## Highlights

• The lag time between coronavirus disease 2019 infection and death differs between prefectures in Japan.
• The lag time varied from the first wave to the seventh wave.
• Using clinical results from the start of infection to death for the case fatality ratio is inadequate.

**KEY POINTS**

**Question:** How did the weekly confirmed case fatality rate (CFR) of coronavirus disease 2019 (COVID-19) change from February 2020 to September 2022, considering the lag between infections and deaths for each prefecture in Japan?

**Findings:** The lag time between weekly infections and deaths during the COVID-19 pandemic differed widely among prefectures in Japan. Additionally, it varied from the first epidemic wave to the seventh epidemic wave. This result affects the CFR estimation.

**Meaning:** The variation in the estimated lag time across prefectures in Japan for different COVID-19 epidemic waves indicates that using clinical results from the start of infection to death for evaluation of the ecological scale of the CFR is inadequate.

Alt-text: Unlabelled box

## Introduction

Coronavirus disease 2019 (COVID-19) is caused by severe acute respiratory syndrome coronavirus-2 (SARS-CoV-2), and was first identified in December 2019 [1]. SARS-CoV-2 is a positive-sense single-stranded RNA virus that belongs to the genus Betacoronavirus [2]. As of 23 June 2021, approximately 180 million people have been infected and about 3.88 million have died worldwide [3]. In Japan, around 790,000 people have been infected and approximately 15,000 have died, causing considerable disruption to civilian life and significant global economic loss. The COVID-19 mortality rate in Japan is the lowest among the world's wealthiest nations, and the estimated excess deaths: 1431–2587; excess percentage: 5.95–10.77%, despite the fact that Japan has the highest proportion of elderly people globally [4].

Most patients infected with SARS-CoV-2 have mild symptoms, such as a dry cough, sore throat and fever. Most cases resolve spontaneously; however, some develop fatal complications, such as organ failure, septic shock, pulmonary oedema, severe pneumonia and acute respiratory distress syndrome (ARDS) [5]. Patients who require intensive care support are older and have multiple comorbidities, including cardiovascular, cerebrovascular, endocrine, digestive and respiratory disease. Additionally, intensive care patients are more likely to report dyspnoea, dizziness, abdominal pain and anorexia [1].

Unlike many Western nations that faced overwhelming numbers of cases of COVID-19 and inflated excess deaths during the early phases of the pandemic in 2020, Japan only saw low levels of transmission and paradoxically observed lower-than-expected (i.e. exiguous) deaths [5]. Reasons suggested for these differences include: (1) Japanese culture and social distancing; (2) exposure to a milder form of SARS-CoV-2 that conferred herd immunity before the spread of a more virulent strain; (3) reduced susceptibility due to angiotensin-converting enzyme 2 receptor expression; (4) distinct human leukocyte antigen that confers immune resistance to CoV-2; and (5) the bacillus Calmette–Guérin vaccine used in Japan confers protection against COVID-19. The lessons behind this success are drawing worldwide attention, and this was probably due, in part, to the declaration of a national state of emergency (SoE) before uncontrolled transmission in April–May 2020. During the SoE, when protective measures such as mask wearing were boosted, businesses (e.g. restaurants and bars) were encouraged to abide by curfews to limit social gatherings in the evenings, and individuals were encouraged to work from home and restrict outings to those that were truly necessary. Unlike many other countries, the SoE provisions in Japan were voluntary (as Japan's constitution forbids enforcement), and the Japanese Government struggled with informing message [6]. While mobility data suggest that many of the population followed these precautions, compliance decreased with each subsequent SoE.

A COVID-19-related case fatality rate (CFR) metric has been developed [7] and reported in emerging infectious diseases, such as severe acute respiratory syndrome (CFR 9.6%) and Middle East respiratory syndrome (CFR 34.5%) [8]. COVID-19-related CFRs are difficult to calculate with certainty; however, according to estimates based on reports from China, the UK, Italy and the *Diamond Princess* cruise ship, the overall death rate from confirmed cases of COVID-19 is around 0.7–1.3%, rising sharply from <0.002% in children aged ≤9 years to 8% in people aged >80, which is much greater than seasonal influenza at approximately 0.1% [1,[9], [10], [11], [12], [13]]. However, CFRs depend on the pandemic phase, which differ in each country as COVID-19-associated deaths are counted differently in different countries [14]. Furthermore, the CFR is contingent on the policies, response and efficiency of local healthcare systems. Normally, mortality rate estimates are based on the number of deaths relative to the number of confirmed cases of infection. However, patients who die on any given day were infected much earlier, so this is not representative of the actual death rate. The denominator of the mortality rate should be the total number of patients infected at the same time as those who died, considering the appropriate lag time [15]. There is a critical need to consider time lags to death when calculating the CFR [16,17]. Without systematic sampling, it is challenging to determine the total number of infected individuals. However, the previously reported number of confirmed infections was speculated to be biased because of limited polymerase chain reaction (PCR) testing, and test capacity varied by prefecture. Yang et al. reported that the median time from symptom onset to radiological confirmation of pneumonia was 5 days [interquartile range (IQR) 3–7 days], from symptom onset to intensive care unit (ICU) admission was 11 days (IQR 7–14 days), and from ICU admission to death was 7 days (IQR 3–11 days) [18]. A median of 13 days passed from confirmation of pneumonia to death ([11–5] + 7 = 13). To the authors’ knowledge, no studies to date have clarified COVID-19 infection and mortality rates in Japan, although it is essential to have reliable estimates of the infection fatality risk so policy makers at local and state levels can make informed decisions.

The aim of this study was to investigate the lag time for reporting COVID-19 infection and mortality related to COVID-19 infection at area block level in Japan, and to compute the CFR, taking lag time into account. This analysis highlights the key determinants of how the SARS-CoV-2 virus spreads and causes death.

## Materials and methods

### Study design

This was an observational study.

### Data source

In order to quantify prefecture level variations in the lag between COVID-19 infection and mortality, daily count data of new confirmed cases of infection and death were collected for each prefecture in Japan from official reports published by the Japan Broadcasting Corporation. Other data sources are summarized in Table S1 (see online supplementary material). Cases of passengers on the *Diamond Princess* cruise ship that docked in Kanagawa were excluded from the data, but cases confirmed after the passengers returned home were included. Additionally, prefecture sociodemographic and environmental data in Japan were collected. In order to focus on elderly mortality in Japan, published estimates of weekly mortality rates by sex and age group were extracted, and the elderly population was defined as those aged >60 years from 2 to 27 September 2020 (Table S1, see online supplementary material).

### Surveillance system and confirmed cases of SARS-CoV-2

At the onset of the COVID-19 outbreak, the Japanese Ministry of Health, Labour and Welfare launched a surveillance system to collect information on people with COVID-19 throughout the country. COVID-19 cases were identified by reverse transcriptase PCR testing for SARS-CoV-2. The present study used publicly available data to calculate the CFR and lag time at prefecture level. As data on individual patients are more challenging to obtain, only aggregated data (i.e. patient counts) were required. The target period was 2 years and 6 months, from 9 February 2020 to 27 September 2022. Based on this data, two variables were defined: weekly counted infection cases and death cases for each prefecture. Weekly count data were used instead of daily count data in the analysis as the trends vary depending on the day of the week; the number on weekend days is relatively small compared with that on weekdays, which may affect the analysis. Furthermore, in order to investigate the elderly population epidemic effect, national open data, such as the Vaccination Record System provided by the Digital Agency, Ministry of Health, Labour and Welfare, were also used (Table S1, see online supplementary material).

The criteria for the epidemic wave definition is that the duration of epidemic waves was based on epidemic curves for each week of diagnosis in Japan. Each epidemic wave was defined as the beginning week when the number of cases ‘increased for at least 3 weeks and was at least 10% of the peak or 1.5 or more than the previous week for 2 consecutive weeks’, and the end week was defined as when the number of cases ‘decreased for at least 3 weeks and was at least 10% of the peak (before the next wave started)’. The first epidemic wave was from Week 13 2020 to Week 20 2020, the second epidemic wave was from Week 26 2020 to Week 39 2020, the third epidemic wave was from Week 44 2020 to Week 8 2021, and the fourth epidemic wave was from Week 9 2021 to Week 24 2021, when the Alpha strain was prevalent. The fifth wave was from Week 28 2021 to Week 38 2021, when the Delta strain was prevalent, and the sixth wave was from Week 51 2021 to Week 24 2022, when the Omicron strain was prevalent [19]. The seventh epidemic wave was from Week 37 2022 to Week 38 2022 (27 September 2022), when data collection was completed at national level. In the seventh epidemic wave, the BA.5 strain was the predominant strain.

### Case fatality rate

According to the World Health Organization definition, two measures are used to assess the proportion of infected individuals with fatal outcomes. The first is the infection fatality ratio, which estimates the proportion of deaths among all infected individuals. The second is the CFR, which estimates the proportion of deaths among the identified confirmed cases [3]. In the present study, the CFR was defined as the number of deaths among persons who tested positive for SARS-CoV-2 divided by the number of confirmed SARS-CoV-2 cases. In order to estimate the area block level CFR considering the lag between infection and death, data for each epidemic wave from February 2020 to September 2022 were analysed. In order to assess mortality in the elderly population, prefecture level CFR was also measured because some prefecture level mortality data were not available in the open data, which may cause bias when calculating area block level CFR.

### Statistical analyses

In order to consider the lag between infection and death for CFR estimation, lag analysis between the onset of COVID-19 infection and related death was conducted. Cross-correlation analysis between the COVID-19 weekly new infection case time series and weekly new death case time series was performed to explore the time lag and correlation coefficient between them. Cross-correlation is a measurement that tracks the movements of two or more sets of time series data relative to each other**.** The following paragraph will explain cross-correlation analysis, taking a typical city (Tokyo with 5375 confirmed cases in the first wave) as an example.

A cross-correlation analysis of each epidemic wave in Japan was conducted to evaluate the relationship between infection cases and death cases. Cross-correlation coefficients were calculated between the COVID-19 infection case time series and the death case time series with ±6-week lags. Let the target cross-correlation coefficient with k weeks lag be $r_{k}^{xy}$, which is computed as follows:

$$r_{k}^{xy}=\frac{\sum \left(y_{i}-y\right)\left(x_{i}-x\right)}{\sqrt{\sum {\left(y_{i}-y\right)}^{2}\sqrt{{\left(x_{i}-x\right)}^{2}}}},$$

where $x_{i}\;$is the normalized COVID-19 weekly new infection case time series, $y_{i}$ is the normalized COVID-19 weekly new death case time series, of the week, and k is the lag. Previous studies have used this cross-correlation method to estimate the lag between two time series [15,20]. These variables are normalized between 0 and 1 as follows:

$$x_{i}\;=\frac{X_{i}\;-min\left(X_{i}\right)}{max\left(X_{i}\right)-min\left(X_{i}\right)},$$

$$y_{i}\;=\frac{Y_{i}\;-min\left(Y_{i}\right)}{max\left(Y_{i}\right)-min\left(Y_{i}\right)}$$

where $X_{i}$ is the number of reported new infection cases in week i, $Y_{i}$ is the number of reported new death cases in week i, and min and max are, respectively, the minimum and maximum values assumed by each variable in its reference interval.

The lag time kwhen the corresponding cross-correlation coefficient was significant and maximized for each prefecture during the period was calculated. A significant positive time lag reflects the time interval between the increase in COVID-19 death cases in response to the rise in COVID-19 infection cases. The maximum correlation coefficient denotes how well the increase in the COVID-19 infection case series predicts an increase in the new COVID-19 death case series. Therefore, the maximum correlation coefficient was used to quantify the relationship between COVID-19 infection cases and death cases.

The cross-correlation analysis was performed for the seven epidemic waves. Using the results of the cross-correlation analysis, CFRs were computed considering the lag time between infection and death at area block level in Japan.

## Results

**Figure 1:**
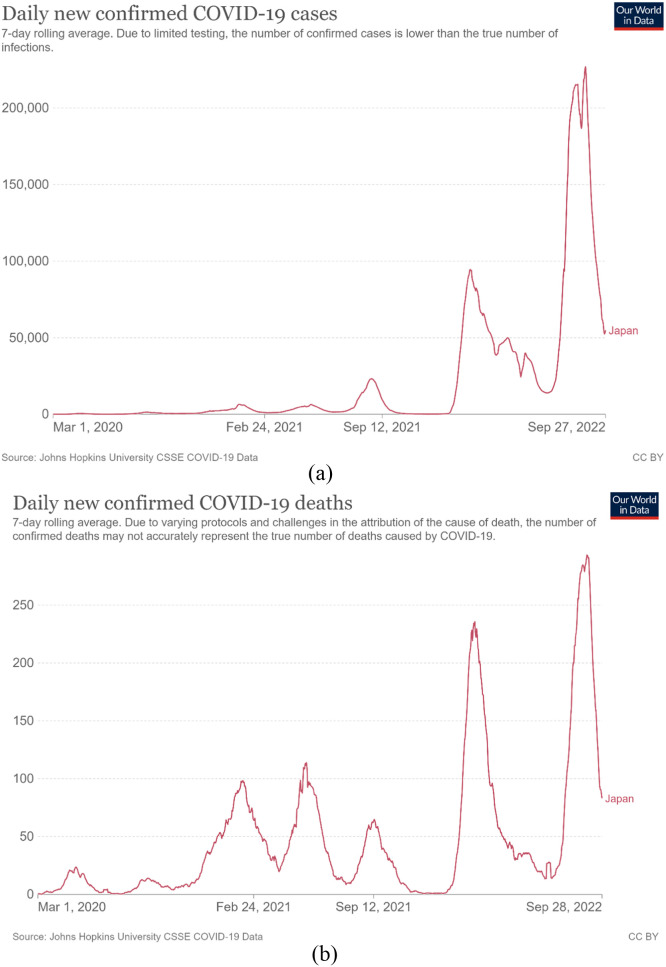
(a) Daily new confirmed cases of coronavirus disease 2019 (COVID-19) in Japan. (b) COVID-19 case fatality rate in Japan. Source: Ritchie H, Mathieu E, Rodés-Guirao L, Appel C, Giattino C, Ortiz-Ospina E, et al. Coronavirus pandemic (COVID-19). 2020. Published online at OurWorldInData.org. Retrieved from https://ourworldindata.org/coronavirus.

Table 1 summarizes the population density (per km^2^ in 2022), the gross domestic product (GDP) per capita (million yen/2018; unit: 1 million yen), and the proportion of elderly (%; >65 years old/2019 in thousands of persons and >75 years old/2019 in thousands of persons) for each prefecture. The prefectures with the highest population densities are Tokyo, Saitama, Kyoto, Kanagawa and Osaka, and the prefectures with the highest GDP per capita are Tokyo, Osaka, Aichi, Kanagawa and Saitama. Among the prefectures in Table 1, Hokkaido, Miyagi and Saitama have the highest aging rates. Figure 1a,b show the daily new confirmed COVID-19 cases and COVID-19 CFR in Japan from March 2020 to September 2022, respectively. The data source access site is shown in Table S1 (see online supplementary material).

**Table 1:** Prefectural characteristics.

2022							2019	2018		
Block	Prefecture	Population	Area	Proportion of Japan		Population density	Gross prefectural product (production side, nominal) (unit: 1 million yen)	Proportion of elderly		
			(km^2^)	(%)		(km^2^)		>65 years old	Aging rate	(%)
				Population	Area			(/’000 persons)		
	All Japan	88,285,927	13,250	70	3.5	6663	580,846,867			
Hokkaido	Hokkaido	3,973,007	802	76	1	4954	20,464,601	5286	1,656	31
Tohoku	Aomori	587,023	164	47.4	1.7	3587	4,533,207	1263	412	33
	Iwate	400,246	89	33.1	0.6	4508	4,847,594	1241	403	33
	Miyagi	1,508,978	267	65.6	3.7	5653	9,829,354	2316	643	28
	Akita	340,699	84	35.5	0.7	4055	3,624,750	981	357	36
	Yamagata	492,405	130	46.1	1.4	3800	4,336,714	1090	358	33
	Fukushima	773,118	189	42.2	1.4	4087	7,987,042	1864	576	31
Kanto	Ibaraki	1,169,451	277	40.8	4.5	4217	14,092,237	2877	833	29
	Tochigi	929,109	209	48.1	3.3	4452	9,261,942	1946	546	28
	Gunma	809,514	215	41.7	3.4	3773	9,308,340	1952	574	29
	Saitama	5,998,734	717	81.7	18.9	8366	23,642,796	7330	1,934	26
	Chiba	4,823,612	674	76.8	13.1	7157	21,279,583	6255	1,721	28
	Tokyo	13,844,009	1092	98.6	49.8	12,680	115,682,412	13,822	3,189	23
	Kanagawa	8,743,513	955	94.7	39.5	9158	35,205,391	9177	2,305	25
Chubu	Niigata	1,119,029	251	50.8	2	4465	9,185,179	2246	716	32
	Toyama	414,349	112	40	2.6	3712	4,910,232	1050	336	32
	Ishikawa	610,464	119	53.9	2.8	5151	4,779,462	1143	334	29
	Fukui	355,428	91	46.3	2.2	3894	3,694,563	774	234	30
	Yamanashi	254,878	60	31.5	1.3	4257	3,566,046	817	248	30
	Nagano	719,893	174	35.2	1.3	4139	8,454,339	2063	651	32
	Gifu	806,499	191	40.8	1.8	4225	7,936,830	1997	595	30
	Shizuoka	2,237,324	445	61.6	5.7	5026	17,866,284	3659	1,081	30
	Aichi	5,942,244	965	78.8	18.7	6157	40,910,717	7537	1,875	25
Kinki	Mie	774,064	190	43.7	3.3	4075	8,086,393	1791	527	29
	Shiga	754,141	130	53.3	3.2	5821	6,922,569	1412	363	26
	Kyoto	2,176,168	268	84.4	5.8	8133	10,846,020	2591	749	29
	Osaka	8,478,518	927	95.9	48.7	9146	41,188,364	8813	2,420	28
	Hyogo	4,306,048	601	78.8	7.2	7165	22,195,171	5484	1,577	29
	Nara	887,863	147	67	4	6036	3,925,192	1339	413	31
	Wakayama	348,232	88	37.7	1.9	3977	3,744,551	935	306	33
Chugoku	Tottori	210,681	54	38.1	1.5	3912	1,893,375	560	177	32
	Shimane	171,792	41	25.6	0.6	4175	2,689,278	680	231	34
	Okayama	917,819	207	48.6	2.9	4427	7,842,490	1898	571	30
	Hiroshima	1,831,138	302	65.4	3.6	6069	11,969,086	2817	817	29
	Yamaguchi	683,695	215	50.9	3.5	3179	6,350,497	1370	465	34
Shikoku	Tokushima	241,941	58	33.6	1.4	4207	3,222,366	736	243	33
	Kagawa	314,892	78	33.1	4.1	4047	4,008,678	962	303	32
	Ehime	720,814	157	54	2.8	4578	5,148,271	1352	441	33
	Kochi	306,502	53	44.3	0.7	5794	2,464,567	706	245	35
Kyushu	Fukuoka	3,786,685	599	73.7	12	6323	19,942,412	5107	1,408	28
	Saga	282,878	67	34.9	2.7	4247	3,219,595	819	244	30
	Nagasaki	631,342	126	48.1	3	5014	4,789,758	1341	429	32
	Kumamoto	865,846	166	49.8	2.2	5217	6,363,425	1757	537	31
	Oita	547,792	121	48.7	1.9	4546	4,525,054	1144	371	32
	Miyazaki	509,617	120	47.6	1.5	4254	3,703,950	1081	342	32
	Kagoshima	660,703	125	41.6	1.4	5287	5,772,861	1614	506	31
Okinawa	Okinawa	1,023,230	144	69.7	6.3	7116	4,633,329	1448	313	22

Sources:

Demographic data collection (2022) https://www.ipss.go.jp/syoushika/tohkei/Popular/P_Detail2022.asp?fname=T12-22.htm.

Prefectural Gross Domestic Product (production side, nominal) *Same for expenditure side (Excel format: 31KB).

https://www.esri.cao.go.jp/jp/sna/data/data_list/kenmin/files/contents/main_2019.html.

Chapter 1: Aging Population (Section 1.4) https://www8.cao.go.jp/kourei/whitepaper/w-2019/html/zenbun/s1_1_4.html.

### Transition in the lag time (standardized) from the first to the seventh epidemic wave

**Figure 2:**
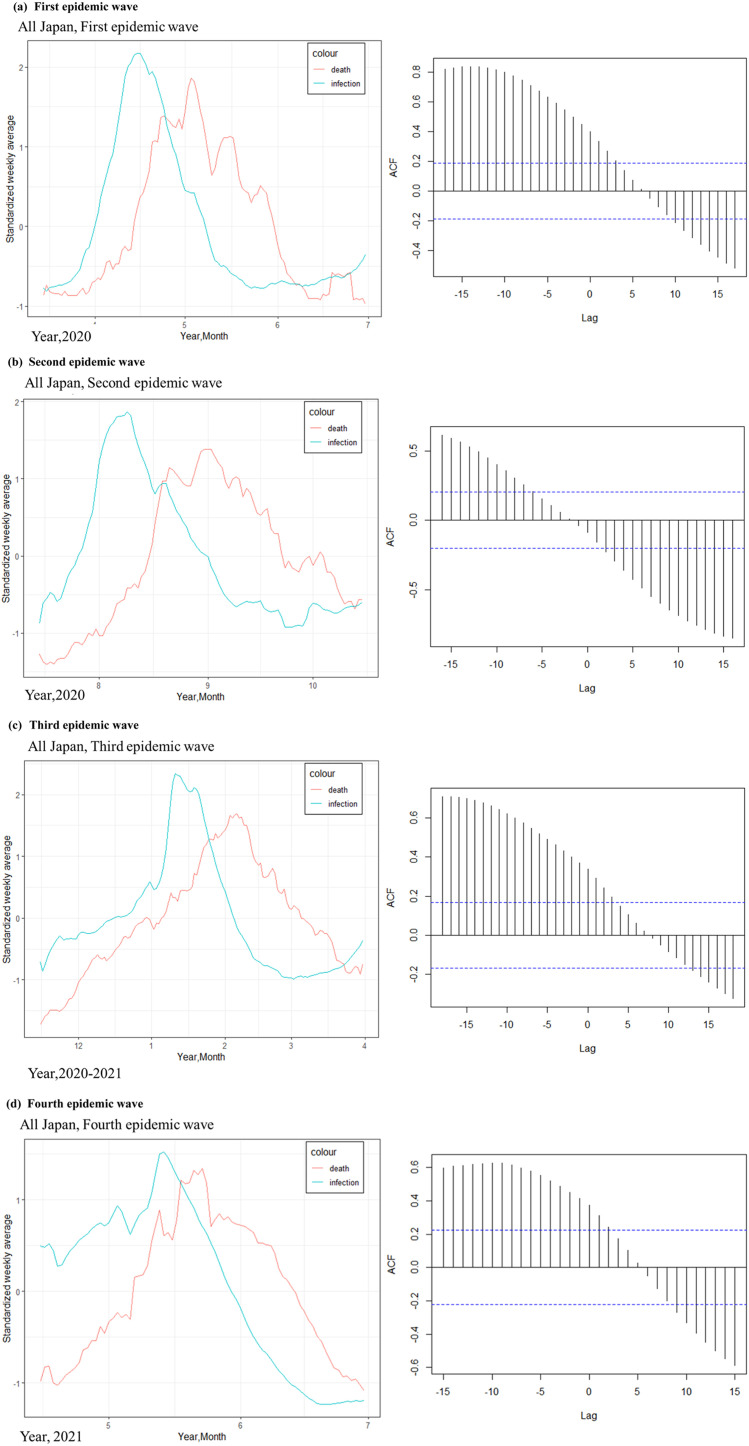

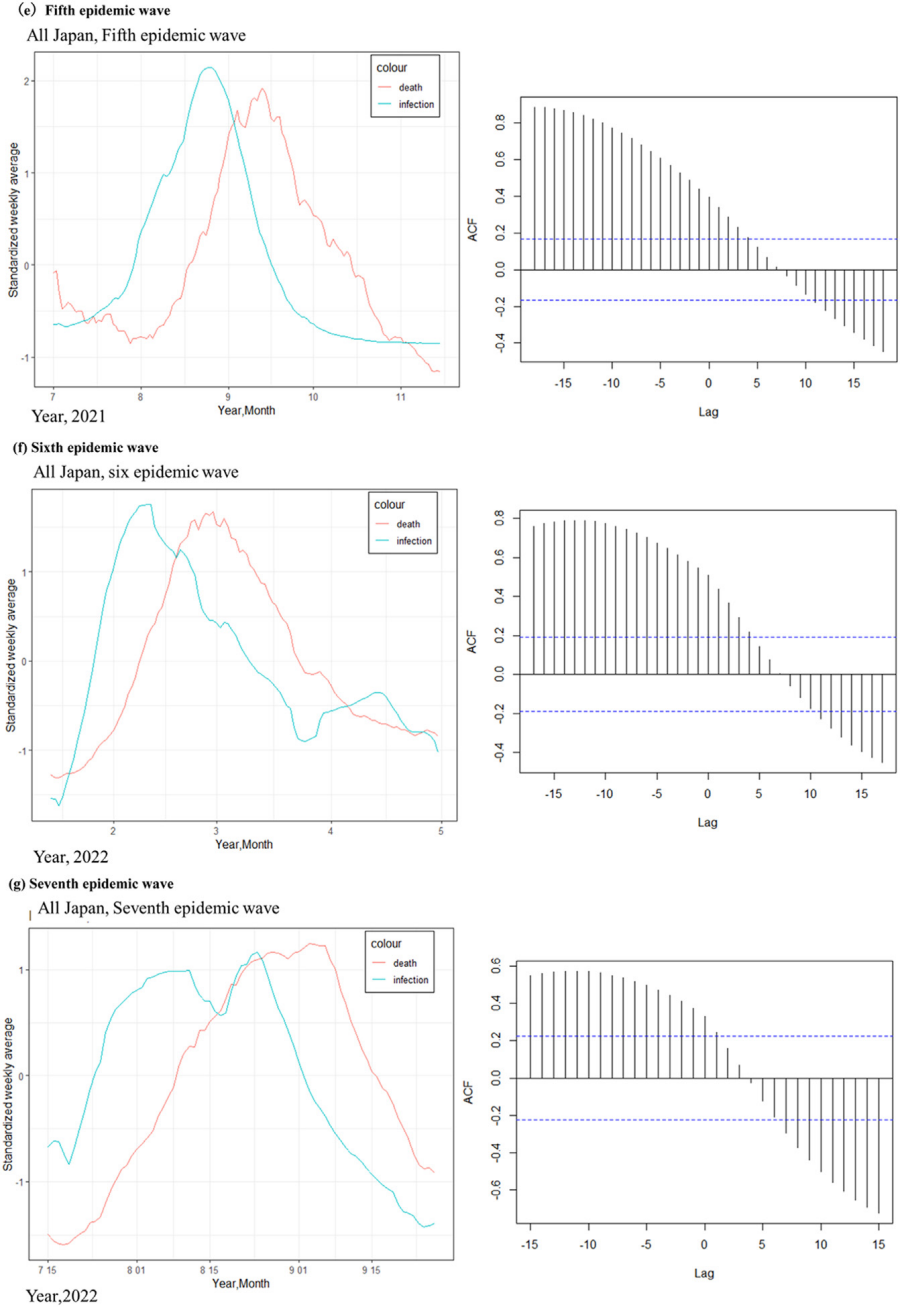
Coronavirus disease-19 epidemic and mortality curve and lag time in Japan.

Table 2 shows the cross-correlation analysis results of the time lag for each COVID-19 epidemic wave for each area block. The lag time between the infection and mortality peaks after normalization at block level for each epidemic wave (from the first to the seventh epidemic wave) is shown in Figure 1. In addition, the lag time between infection (symptomatic) and mortality in each epidemic wave in Japan is shown in Table 2. The epidemic waves in Hokkaido and Okinawa prefectures were shorter than those for the epidemic waves in other area blocks. The fourth epidemic wave was shorter than the other epidemic waves. Many areas had a longer lag in the third wave compared with the other epidemic waves. Figure 2 shows the standardized COVID-19 infection and death cases in Japan. The cross-correlation between the number of infections and the number of deaths peaked from a lag of 1 week to 4 weeks. The lag time was calculated by area block base, not by prefecture base, because the peak could not be identified. For Hokkaido and Okinawa prefectures, the lag times (from 7 days to 16 days) were shorter than those in the other area blocks (from 21 days to 29 days). The standardized infectious and mortality curves and CCF for lag time by area block are shown in Figure S2 (see online supplementary material). This result suggests that the increase in new COVID-19 infection cases was associated with the number of new COVID-19 deaths. Moreover, it implies that the number of infection cases at timet+3 weeks is significantly associated with the number of deaths at time *t*. Therefore, an increase in the number of infections leads to an increase in the number of deaths 3 weeks later in Tokyo for each wave. This study revealed spatial heterogeneity in lag time among prefectures, suggesting the existence of regional differences in the COVID-19 situation in Japan. In Figure S1 (see online supplementary material), the cumulative mortality number (stratification by age group) is shown, suggesting that the mortality of elderly males (age >80 years) increased in early 2022 (sixth epidemic wave).

**Table 2:** Lag time and its autocorrelation between infection (symptomatic) and mortality in each epidemic wave in Japan.

Block	First epidemic wave (autocorrelation)	Second epidemic wave (autocorrelation)	Third epidemic wave	Fourth epidemic wave	Fifth epidemic wave	Sixth epidemic wave	Seventh epidemic wave
All Japan	14 (0.840)	16 (0.621)	18 (0.730)	10 (0.674)	18 (0.884)	14 (0.812)	12 (0.577)
Hokkaido	14 (0.606)	-	15 (0.487)	15 (0.724)	11 (0.711)	10 (0.929)	6 (0.831)
Tohoku	-	-	26 (0.330)	22 (0.609)	26 (0.661)	27 (0.745)	24 (0.653)
Kanto	24 (0.380)	26 (0.430)	26 (0.192)	23 (0.272)	26 (0.449)	28 (0.760)	24 (0.411)
Chubu	25 (0.505)	27 (0.243)	27 (0.575)	24 (0.281)	27 (0.673)	29 (0.776)	26 (0.698)
Kinki	24 (0.498)	26 (0.472)	26 (0.686)	23 (0.879)	26 (0.609)	28 (0.790)	24 (0.613)
Chugoku	-		25 (0.680)	21 (0.499)	25 (0.554)	26 (0.504)	23 (0.845)
Shikoku	-	-	-	-	-	-	-
Kyushu	22	26	26	23	26	24	24
Okinawa	7 (0.704)	13 (0.577)	-	-	-	-	-

Hokkaido block (prefecture): Hokkaido.

Tohoku block (prefecture): Aomori, Iwate, Miyagi, Akita, Yamagata, Fukushima.

Kanto block (prefecture): Ibaragi, Tochigi, Gumma, Saitama, Chiba, Tokyo, Kanagawa.

Chubu block (prefecture): Niigata, Toyama, Ishikawa, Fukui. Yamanashi, Nagano, Gifu, Shuzuoka, Aichi.

Kinki block (prefecture): Mie, Shiga, Kyoto, Osaka, Hyogo, Nara, Wakayama.

Chugoku (prefecture): Tottori, Shimane, Okayama, Hiroshima, Yamaguchi.

Shikoku (prefecture): Tokushima, Kagawa,Ehime, Kochi.

Kyushu (prefecture): Fukuoka, Saga, Nagasaki, Kumamoto, Oita, Miyazaki, Kagoshima.

Okinawa (prefecture): Okinawa.

**Figure 3:**
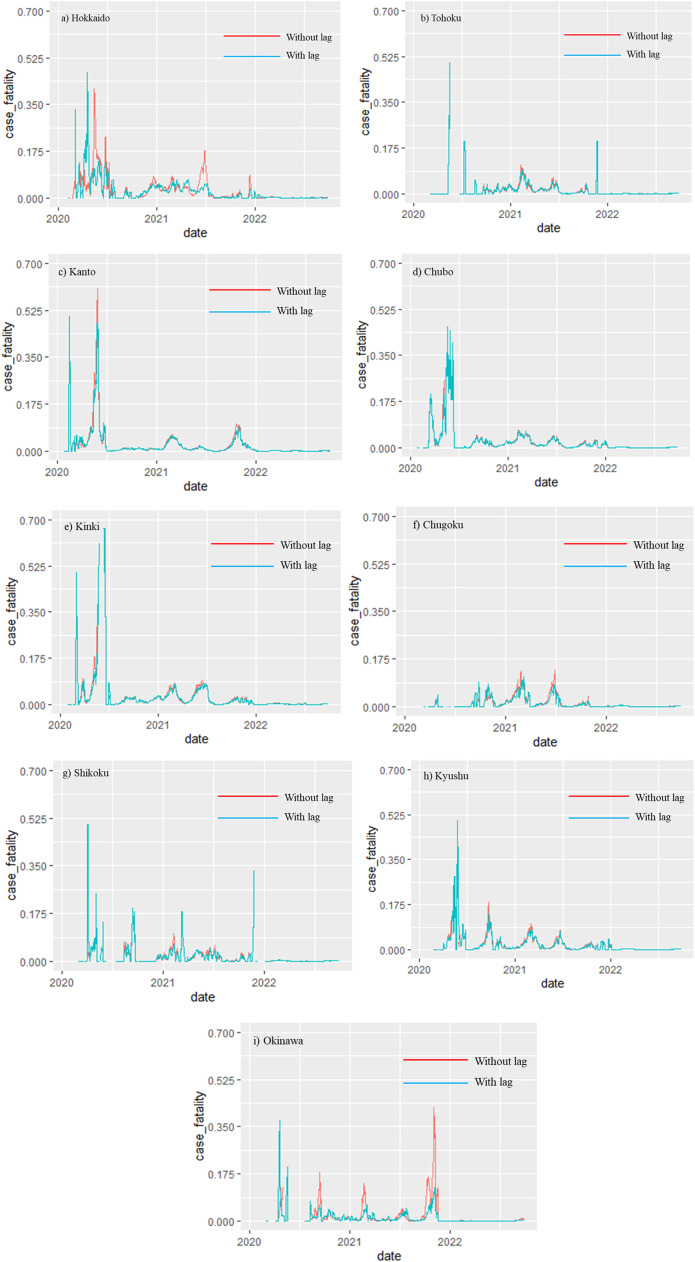
Case fatality rate in each area block.

**Figure 4:**
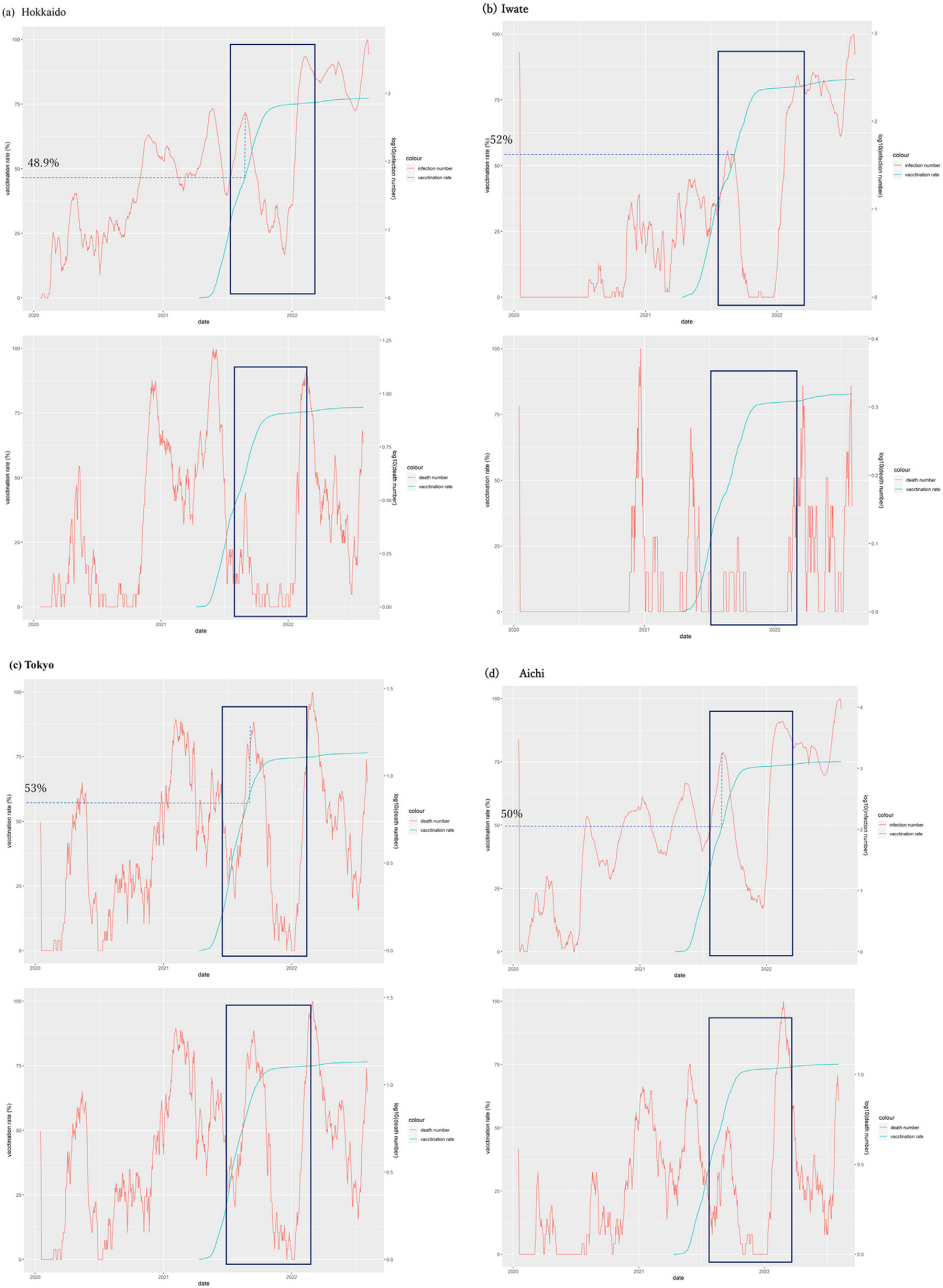

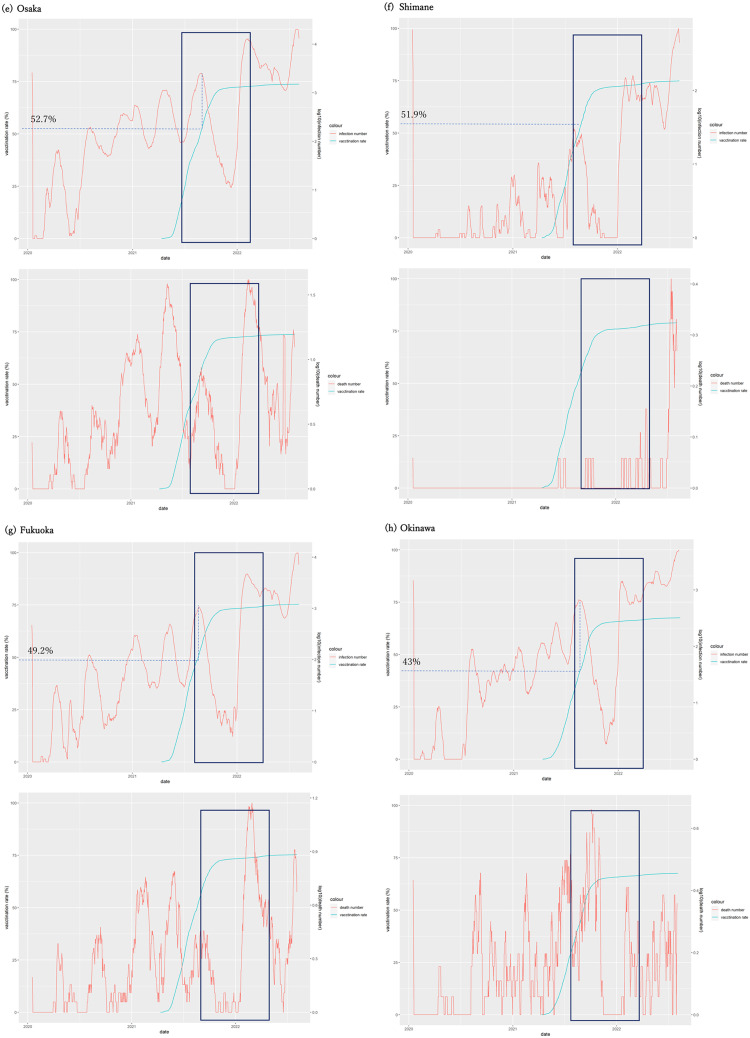
Seven-day moving average for number of infections, number of deaths and vaccination coverage in the selected prefectures.

**Figure 5:**
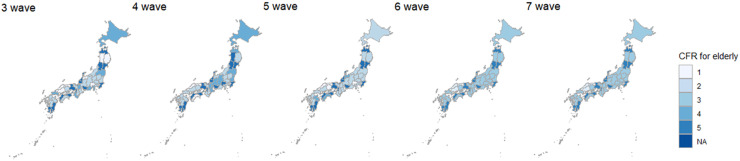
Case fatality rate in each epidemic wave in Japan (>60 years of age).

Figure 3 shows the CFR (with and without lag time) by area block. Some epidemic peaks in the CFR were overestimated, such as Hokkaido area in early 2020 and Okinawa area in late 2021. Figure 4 shows the 7-day moving average for number of infections, number of deaths and vaccination coverage for people aged >60 years in Hokkaido, Iwate, Tokyo, Aichi, Osaka, Shimane, Fukuoka and Okinawa prefectures. These analysis results indicate that the 7-day moving average for number of infections did not reach the lowest level during or just after vaccine coverage in urban areas, such as Tokyo, Aichi, Osaka and Fukuoka prefectures; on the other hand, the number of infections in rural areas, such as Iwate and Shimane prefectures, decreased to the minimum just after vaccine coverage. Some prefectures had very low numbers of deaths, such as Iwate and Shimane prefectures, and exact peaks were not identified. The maps in Figure 5 show the distribution of new COVID-19 infections and deaths in Japan.

### Spatio-temporal CFR estimation dynamic with lag time for the elderly population

Figure 5 shows the CFR change dynamic for the elderly population (age >60 years) in each epidemc wave. The average CFR in elderly people in Japan decreased from 8.4% (third epidemic wave) to 0.7% (seventh epidemic wave). For some prefectures, mortality data in high density populations were not available, such as Osaka and Hyogo, so the results may be an underestimate.

### CFR analysis for mortality and infection number

**Figure 6:**
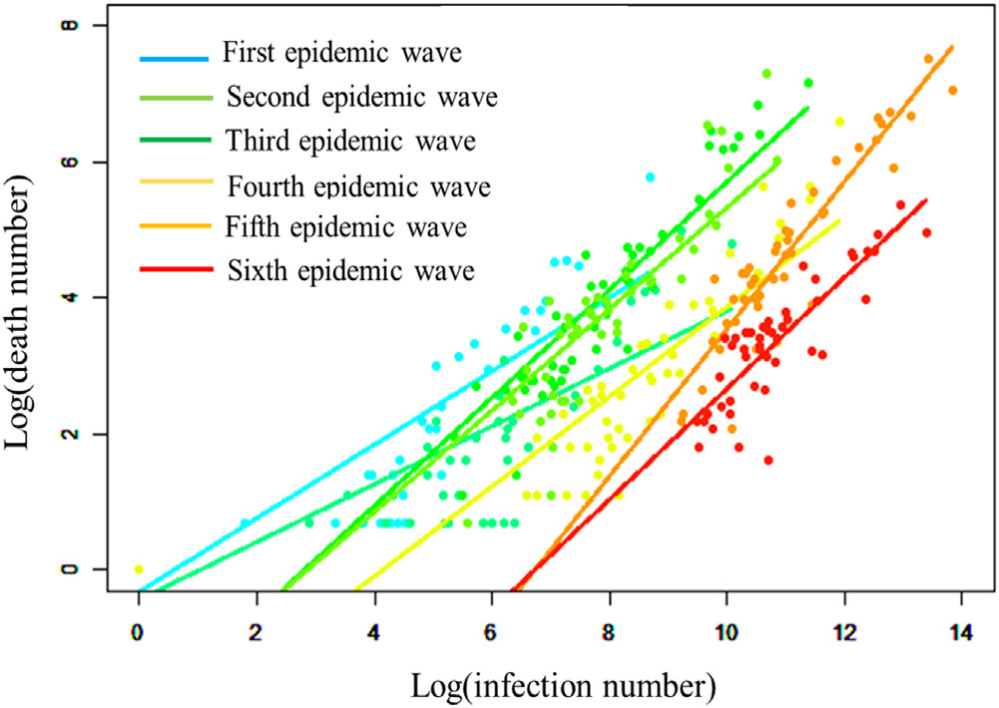
Regression analysis for the numbers of infections and deaths in each epidemic wave.

Figure 6 shows the regression analysis of the log of the mortality and infection numbers of COVID-19 infection in each epidemic wave. The regression models for each epidemic wave were: first epidemic wave, logy=0.54logx−0.34; second epidemic wave, logy=0.43logx−0.46; third epidemic wave, logy=0.79logx−2.25; fourth epidemic wave, logy=0.75logx−2.16; fifth epidemic wave, logy=0.66logx−2.75; sixth epidemic wave, logy=1.08logx−7.32; and seventh epidemic wave, logy=0.82logx−5.53.

## Discussion

This study investigated the relationship between mortality and infection to assess the CFR from 1 February 2020 to 28 September 2022. The results suggest that the lag time between infection with COVID-19 and mortality by the time-lag plots of the confirmed infected populations ranged from 7 to 29 days. The adjusted CFR varied by geographical area, and the CFR without lag time tended to be overestimated from the CFR with lag time. Initial vaccine coverage and mortality were monitored at prefectural level. The epidemic curve was found to peak when it reached around 43–50% vaccine coverage, and the number of infections was lower in rural areas compared with urban areas. The CFR focusing on the elderly population (age >60 years) was calculated with lag time, and later epidemic waves were found to have lower CFRs compared with earlier waves. Additionally, the regression log–log plot for numbers of infections and deaths revealed that the regression line coefficient was highest in the fifth wave.

For the change in lag time from the initial epidemic wave, such as the second wave and the third wave, many prefectures had a significant positive correlation for lag time. This implies that infection with COVID-19 began to spread to almost all areas of Japan in the third wave. Another important point is that the third wave had varied lag times. This is in contrast to the first and second waves, for which all prefectures had similar results. Testa et al. showed the lagged connection between COVID-19 cases and deaths in the USA in 2020, and deaths attributable to COVID-19 increased 4–6 weeks after observed surges in case rates [21]. However, the magnitude of the subsequent surge in deaths attributable to COVID-19 was affected by the age distribution of the population and the age-specific COVID-19 case rates. In the present study, the magnitude of the subsequent surge in deaths attributable to COVID-19 varied between waves; however, the lag times were shorter than those reported by Testa et al. The present data revealed that the mortality rates in the more recent waves were lower, and the exact peak trend may be difficult to follow from the data.

The CFR calculation for the proportion of individuals diagnosed with a disease who die from that disease leads to a wide variation in CFR estimates over the course of an epidemic, and may be biased. If people who are ill with the disease die more quickly than those who had recovered, the CFR may be overestimated. If the reverse is true, it may be underestimated. Using the CCF method to estimate the most appropriate lag time to calculate the CFR is a good way to avoid a biased estimate. However, the lag time could be shortened to 1 week for various reasons, such as a shortage of medical supplies, the proportion of elderly patients, or the mutation of SARS-CoV-2. The present study also estimated the adjusted CFR based on the lag time between mortality and onset of COVID-19 infection. The CFR was reported to be 15% (6/41) in Wuhan, China, during the initial period [22]. Subsequently, the CFR decreased to 4.3% [1]. Abou Ghayda et al. reported meta-analysis results showing that the CFR pattern was approximately 2.0% and 3.0% in Asia until 19 February 2020. The values increased to >4.0% before gradually decreasing again. Until 30 October 2020, the fixed model showed a CFR of 1.6%, while the random model showed a value of 1.5% [23]. These figures are consistent with the present results. The standard of care likely evolved over the course of the outbreak.

The present study showed that the CFRs for each epidemic wave changed over time. di Lego et al. reported that a decrease in CFR may not imply that vaccines are effective in reducing deaths, and testing for COVID-19 infection in vaccinated people is important to track the pandemic. In April 2021, priority vaccination of the elderly (approximately 36 million people) commenced in Japan. By the end of July 2021, it is estimated that approximately 80% of elderly people had been vaccinated twice, which almost achieves the goal of double-inoculating those elderly people who wished to be inoculated (Figure 5). A declining CFR, starting from the fifth wave (July 2021–September 2021), could indicate that the virus is reducing the mortality rate because of vaccination. Onozuka et al. found a decrease in mortality during the COVID-19 outbreak in February–December 2020 using a two-stage interrupted time series design in Japan [24]. From analysis of the national surveillance data from the first 4 months of the nationwide vaccination campaign (24 January–3 April 2021), Haas et al. reported that, as vaccine coverage increased, the incidence of SARS-CoV-2 outcomes reduced in all age groups in Israel [25]. The present results were inconsistent with their conclusion. On the other hand, as there are no data on the positivity rate among vaccinated and unvaccinated people, it is very difficult to include vaccine effectiveness, as suggested by di Lego [26].

In the initial stage, the regression coefficient *k* for the log–log plot of the numbers of infections and deaths in each epidemic wave was >1, which could indicate that the two populations are physically separated, such as in hospitals, nursing homes or other facilities housing vulnerable populations, where infection spreads easily with a high mortality rate. In the present results, in the sixth wave, *k* in the log–log plot was >1. In the fifth wave, the Alpha strain was replaced with the Delta strain, which is considered to be more infectious and to be associated with a higher risk of hospitalization [27]. In June 2021, the emergency measures were lifted. The spread of infection in Tokyo metropolitan area was clear by late June 2021, and in Kansai area by July 2021. This cluster occurred in dormitories, child care facilities and live music clubs, and the epidemic spread from there. Furthermore, the infection spread sequentially from areas with high concentrations of human flow into the city centre, and from urban to rural areas. As of October 7, the total number of vaccinations was 172,127,058, of which 64,553,077 were administered to the elderly (65 years and older) and 17,523,553 were administered in the workplace. The one or more times vaccination rate for the elderly was 90.8% of the population aged 65 years and over (35,486,339 persons), and the two-dose completion rate was 89.6% [27]. Thus, vaccination status affects the coefficient regression line in subsequent epidemic waves.

Basellini et al. reported that a time lag of at least 5 weeks was needed for a positive association between mobility and mortality in England [28]. The present study detected a lag between infection and death of 2 weeks, which is consistent with previous reports on the natural history of COVID-19 infection. For example, the incubation period (i.e. from infection to symptom onset) can last up to 2 weeks [11.5 days, 95% confidence interval (CI) 8.2–15.6 days] [29], and the course of disease (i.e. from symptom onset to death) can last up to 3 weeks (17.8 days, 95% CI 16.9–19.2 days) [6]. The lag should be longer in prefectures with better health care, and most deaths tend to occur in older patients. This lag will be affected by measures taken to protect the elderly population. Most critically ill and deceased COVID-19 patients did not develop severe clinical manifestations in the early stages of the disease. Some patients only showed a mild fever, cough or muscle soreness. However, the condition of these patients deteriorated suddenly in the later stages of the disease or in the process of recovery. ARDS and multi-organ failure occurred rapidly, resulting in death within a short time. Additionally, cytokine storm is one of the major causes of ARDS and multi-organ failure.

The mortality rate due to COVID-19 in Japan decreased from the first wave to the seventh wave. The Japanese people have followed the Japanese Government's ‘three Cs’ slogan to avoid closed spaces, crowded places and close-contact situations. Additionally, areas from buildings to taxis have improved ventilation, including the use of carbon dioxide monitors to show that indoor air is being exchanged. Other populations have returned to everyday life; however, Japanese people appear to remain cautious about moving around. Nevertheless, the changes seen in some regions from the second wave to the third wave, in which the lag time from a PCR-positive test to death became shorter, may be attributable to the following reasons: (1) the hospitalization criteria for patients with COVID-19 are not clear, and patients who typically require hospitalization are treated at home; (2) the number of beds for severe patients exceeded 100% capacity at the peak of the third wave in some regions, and the number of occupied beds for mild to moderate patients was close to 100% in some regions; and (3) facilities may avoid shifting to extracorporeal membrane oxygen due to an increased patients. After the SARS-CoV-2 variant B.1.1.529, referred to as the Omicron variant, was first reported in South Africa in November 2021, it spread rapidly to overtake the previously dominant Delta variant globally by the end of 2021. Rapid replacement of the Delta variant was observed in Japan, with the Omicron variant reaching 92% of the total newly-detected COVID-19 cases on 10–16 January 2022 [30]. The decrease in the mortality rate is thought to be due to the evolution of medications and ventilator therapies. Particularly between the second and third waves, antiviral and anti-inflammatory therapies began to be covered by health insurance in Japan. In particular, in the variant shift from the Delta variant to the Omicron variant, Suzuki et al. reported that the Omicron variant was associated with a reduction in hospitalization and risk of pneumonia compared with the Delta variant in a case–control study in 2022 [31]. From 2021 to 2022, vaccination rates increased, and new medicines such as sotrovimab, nirmatrelvir/ritonavir and molnupiravir became available to treat patients with mild COVID-19 in Japan, which may explain the decreased severity of patients in Japan. Considering the lag time between reporting infection and death, several studies have shown an influence on the CFR [32]. As the number of severe infected cases increases, patients do not receive the appropriate treatment, such as ventilator support or extracorporeal membrane oxygen; therefore, building adequate healthcare capacity is the most important way to mitigate future case fatalities. Ko et al. suggested that the number of severe cases was positively associated with the CFR among people aged in their 70s, 80s and 90s, but not in people aged <60 years in Tokyo [33].

The present study also revealed that there were increases in mortality accumulation in males in their 70s and 80s, and in females in their 80s and 90s (Figure S1, see online supplementary material). Infection with the Omicron variant has been reported to be associated with lower risk of hospitalization and severe illness compared with the Delta variant. From the beginning of the outbreak until the Delta strain epidemic, many cases of severe respiratory failure due to viral pneumonia were caused by typical novel coronavirus infection. During the Omicron strain epidemic, diseases other than pneumonia were the primary cause of death, such as worsening of an underlying disease from before hospitalization or the development of another complication during hospitalization. The rate of severe cases has decreased and the proportion of hospitalized elderly patients has increased since the outbreak in the summer of 2021. Furthermore, the rate of use of artificial respiration/nasal high-flow and the rate of prescriptions for steroids were lower among the fatalities in summer 2022 than in 2021. In the case of deaths at home in July and August 2021, approximately 80% of the deaths were among persons aged ≥70 years, similar to the overall trend of deaths over the same period, suggesting that there were many deaths due to factors other than the type of variant of SARS-CoV-2 [34].

### Findings

This study analysed the lag between case onset and death in each phase of the COVID-19 pandemic in Japan. This lag was found to vary considerably between prefectures. In some cases, however, the peak in the number of deaths preceded the peak in the number of infections.

### Limitations

This study has a few limitations. First, the number of actual deaths is not necessarily equivalent to the number of reported deaths, as many cases and deaths due to COVID-19 are under-reported [35]. Furthermore, cases of COVID-19 infection may be under-reported, especially in the first epidemic wave, because of the limited accessibility of hospitals with PCR testing capacity in early 2020 in Japan. When PCR testing is not widely available and proactive contact tracing and containment are not employed, a smaller denominator results in skewing of the CFR calculation, making the CFR higher. Furthermore, asymptomatic COVID-19 cases, patients with mild symptoms and misdiagnosed individuals may not be included in the denominator, leading to underestimation of the total case count and overestimation of the CFR. Second, the time lag between the confirmation of infection and reporting of COVID-19 deaths is not compatible with the biological course of COVID-19, but is consistent with the health management in each prefecture in Japan. For example, a growing number of Japanese people have been reported to die from COVID-19 at home as more infectious variants of SARS-CoV-2 fuel new waves, and hospital resources are stretched to the verge of collapse [36]. Moreover, the time lag is highly variable because patients with COVID-19 may be tested at different timepoints due to different disease presentations as well as regulatory and organizational shortages. Third, the study had an observational design, and may be confounded by age distribution in terms of mortality. However, it was not possible to adjust for age. For example, Russell et al. reported that the CFR on the *Diamond Princess* for individuals aged ≥70 years was 13% (95% CI 5.2–26), which suggests strong effects of age and comorbidities on the risk of mortality [8].

## Conclusions

This study analysed the time lag between COVID-19 infection and death in the seven waves of the COVID-19 pandemic in Japan. CFR was estimated for COVID-19 cases in Japan. The peak in the number of deaths preceded the peak in the number of infections in some cases, possibly biased by age distributions in the observed prefectures and by local differences concerning when new cases and deaths are reported. Assessment of the severity of COVID-19 is crucial to determine the appropriateness of mitigation strategies and to plan healthcare needs at prefectural level in Japan as epidemics unfold. The analyses presented in this study will be extended to other countries, and updated regularly during the global COVID-19 outbreak.

## Conflict of interest statement

DN is an employee of Giliado, Japan. The other authors declare that they have no known competing financial interests or personal relationships that could have appeared to influence the work reported in this paper.

## Appendix Supplementary materials

Image, application 1

Image, application 2
