# Supplementary material for: Case fatality rate considering the lag time from the onset of COVID-19 infection to related death from 2020 to 2022 in Japan
Source: IJID Reg. 2023 May 1;8:36–48. doi: 10.1016/j.ijregi.2023.04.013 (PMC10149354; doi:10.1016/j.ijregi.2023.04.013)
Supplement: Supplementary file 1 [file mmc1.docx]

**Supplement　Figures**

Cumulative mortality number (n)

Year,month

Supplement Figure 1 (a) Cumulative mortality number, Male, all Japan.

Reference <https://covid19.mhlw.go.jp/> https://www.stat.go.jp/data/nihon/02.htm

Cumulative mortality number (n)

Year,month

Supplement Figure 1 (b) Cumulative mortality number, Female, all Japan.

Reference <https://covid19.mhlw.go.jp/> <https://www.stat.go.jp/data/nihon/02.htm>

**Supplement Figure 2 Standardized infectious and mortality and CCF for lag time**

1. **Hokkaido block**

**
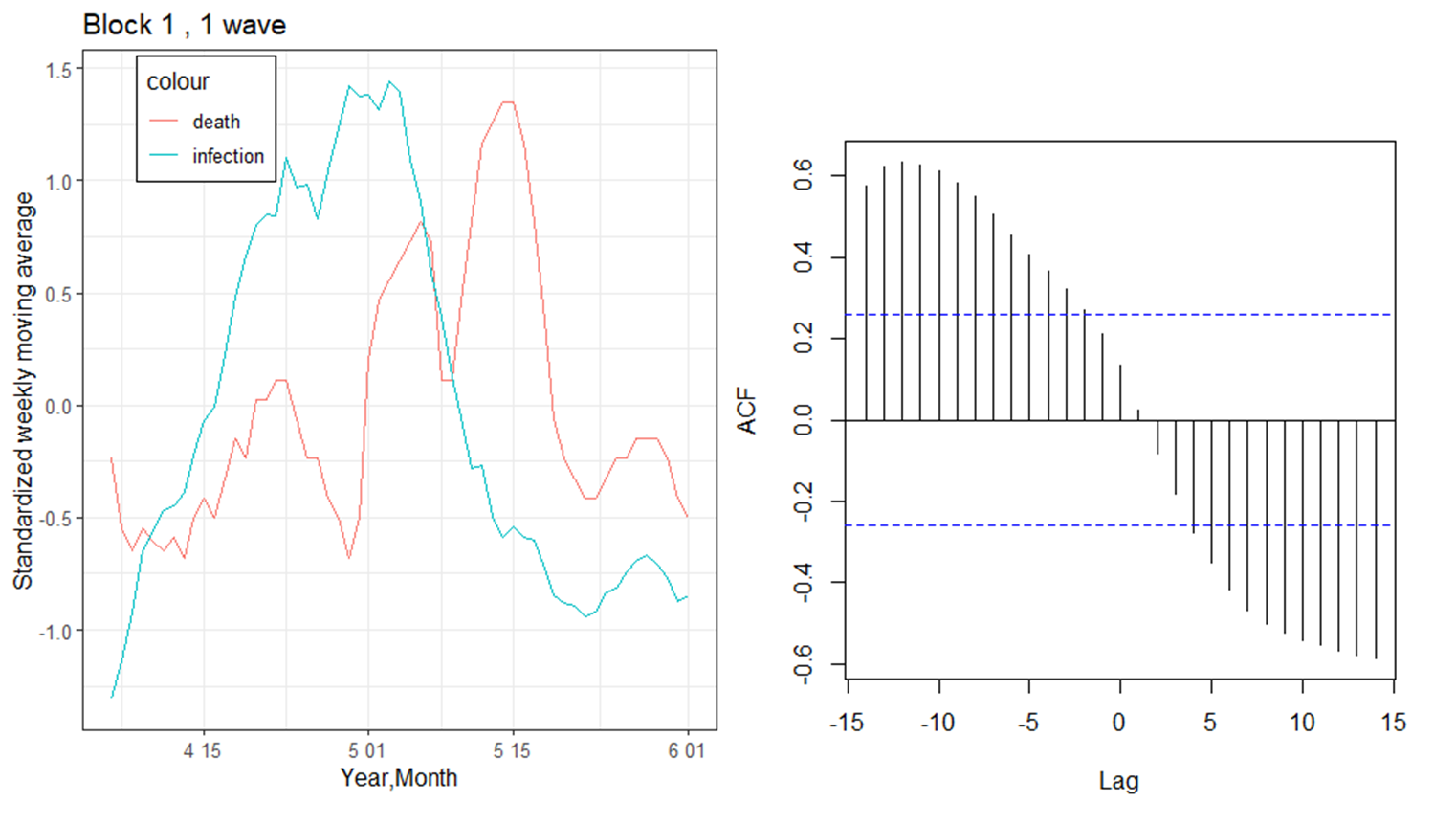
**

**
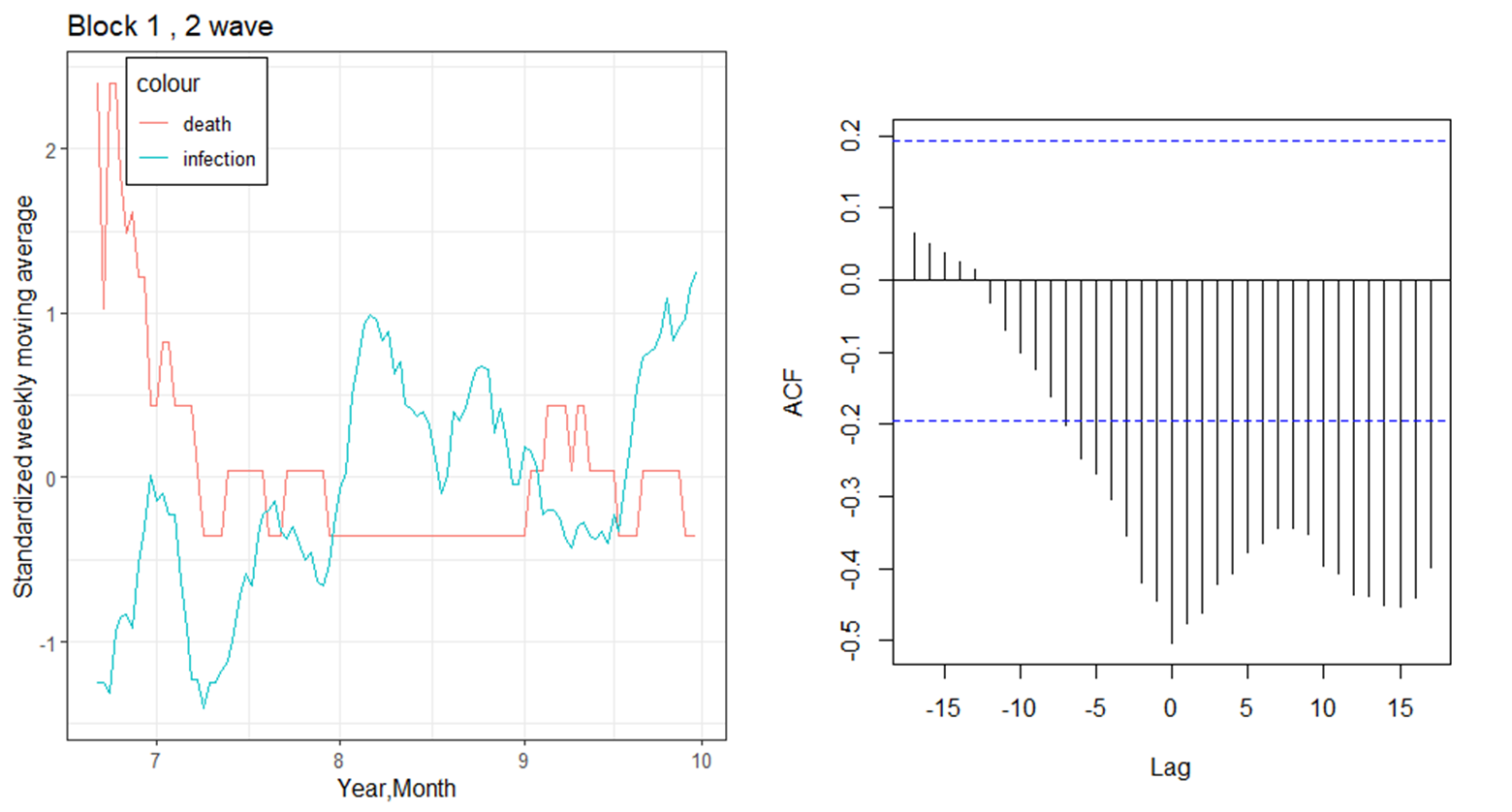
**

**
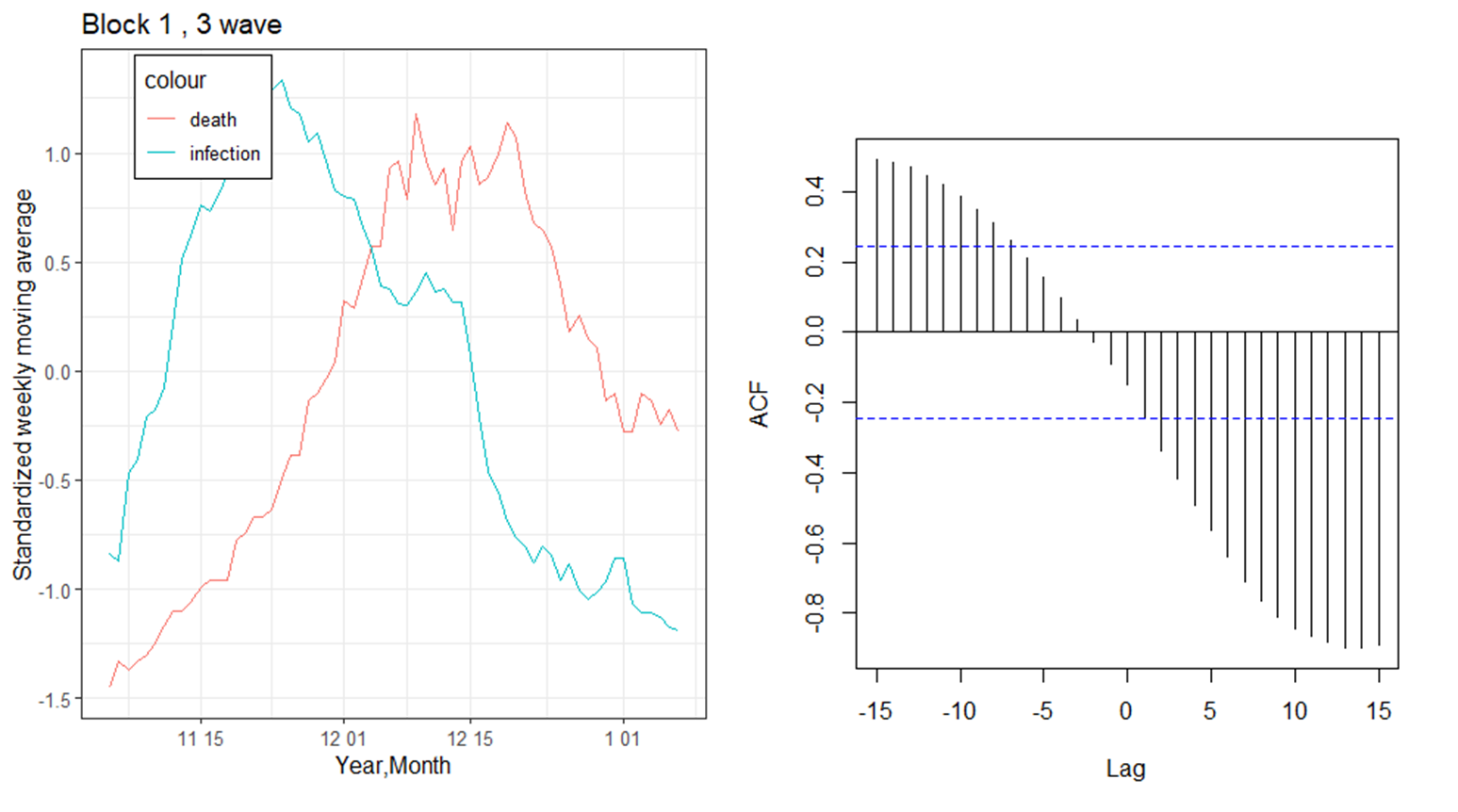
**

**
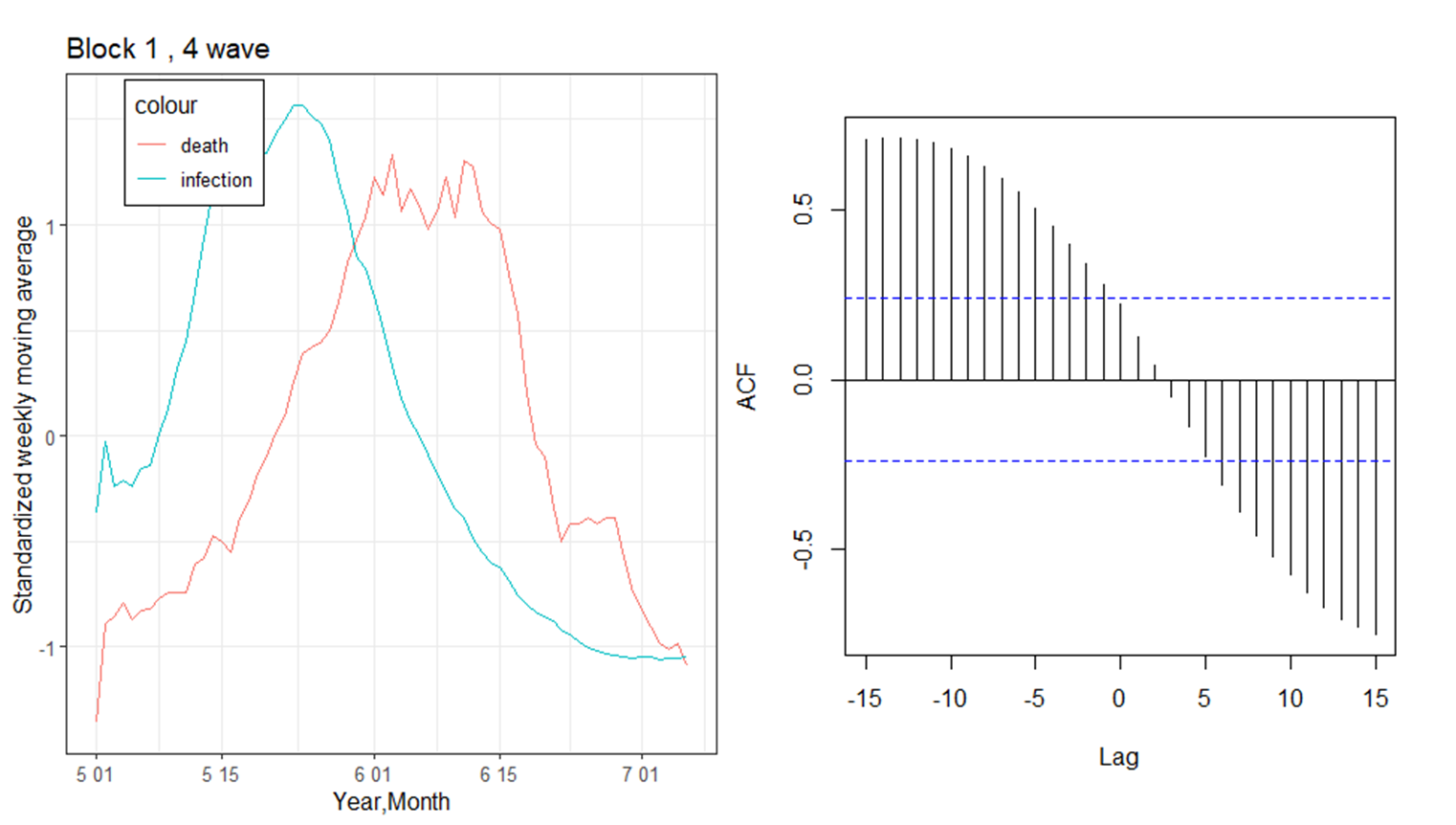
**

**
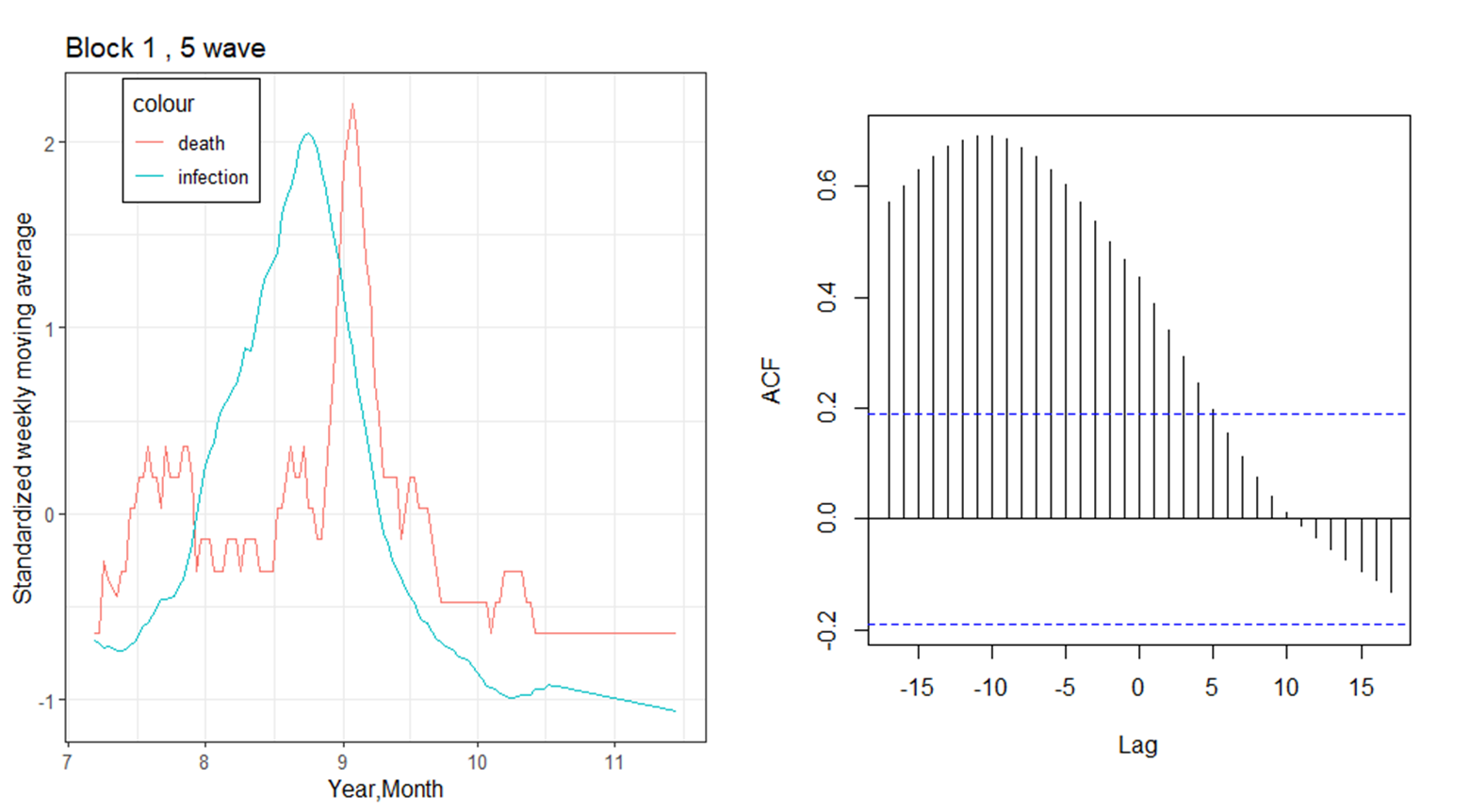
**


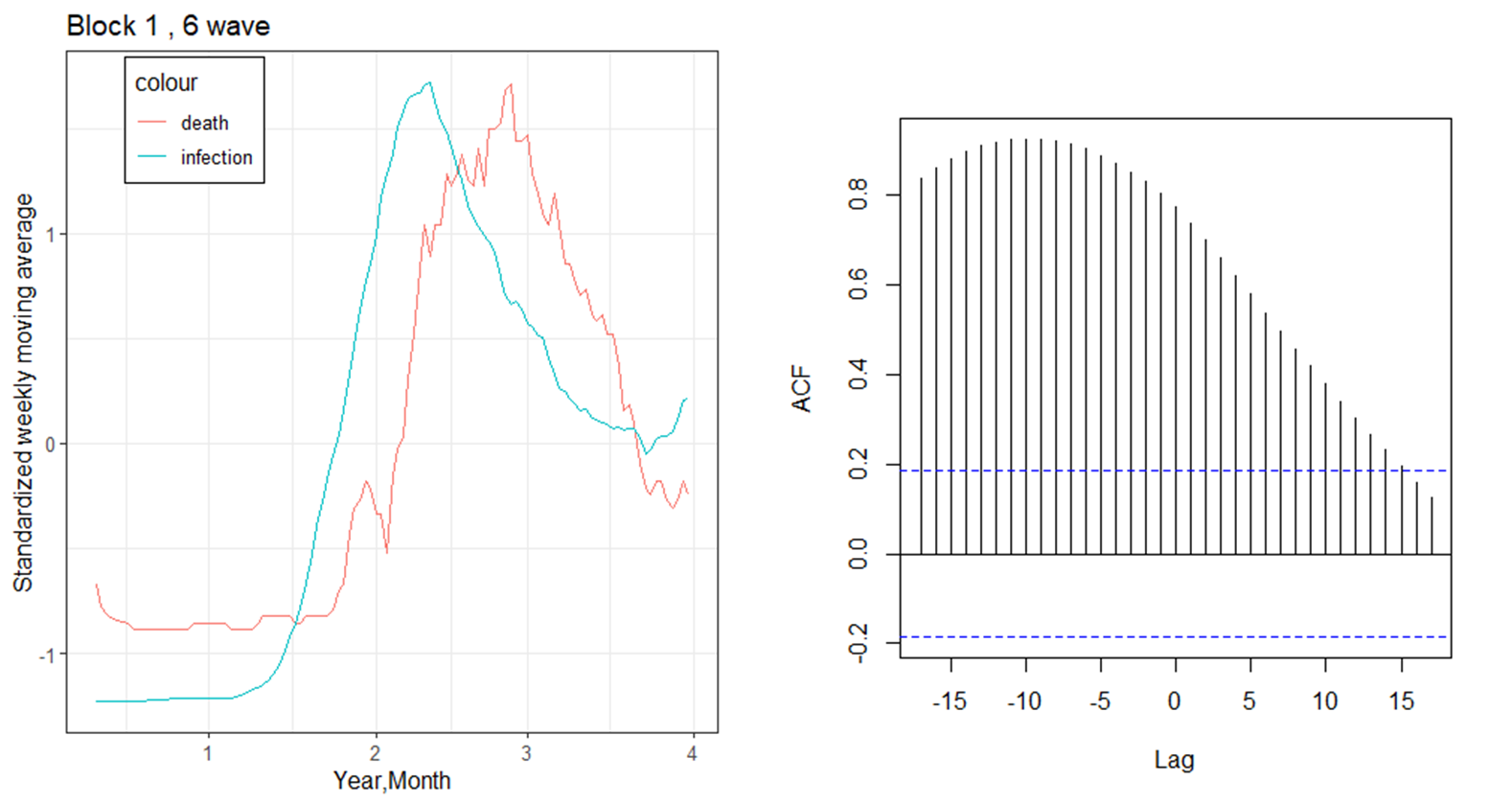


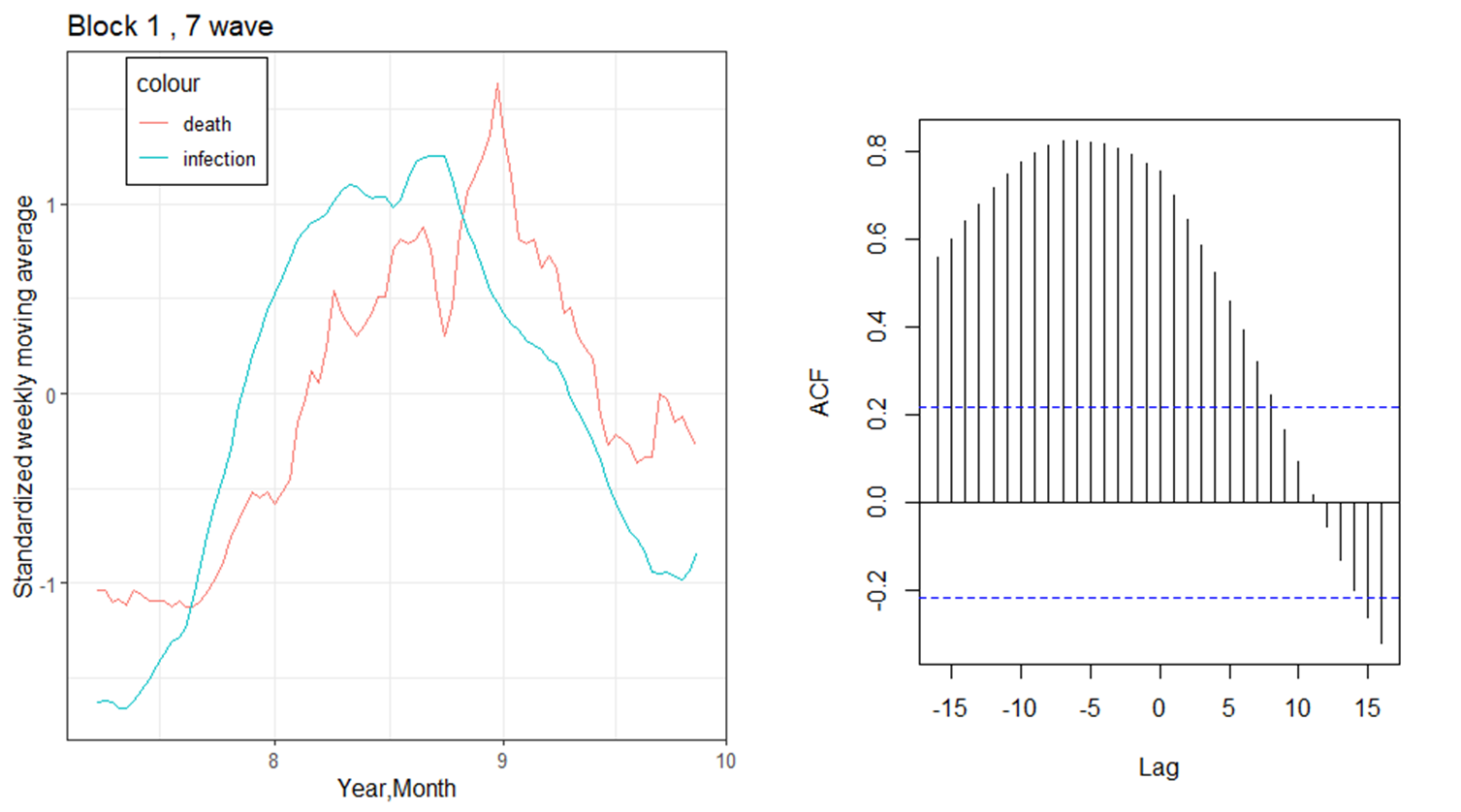


1. **Tohoku block**


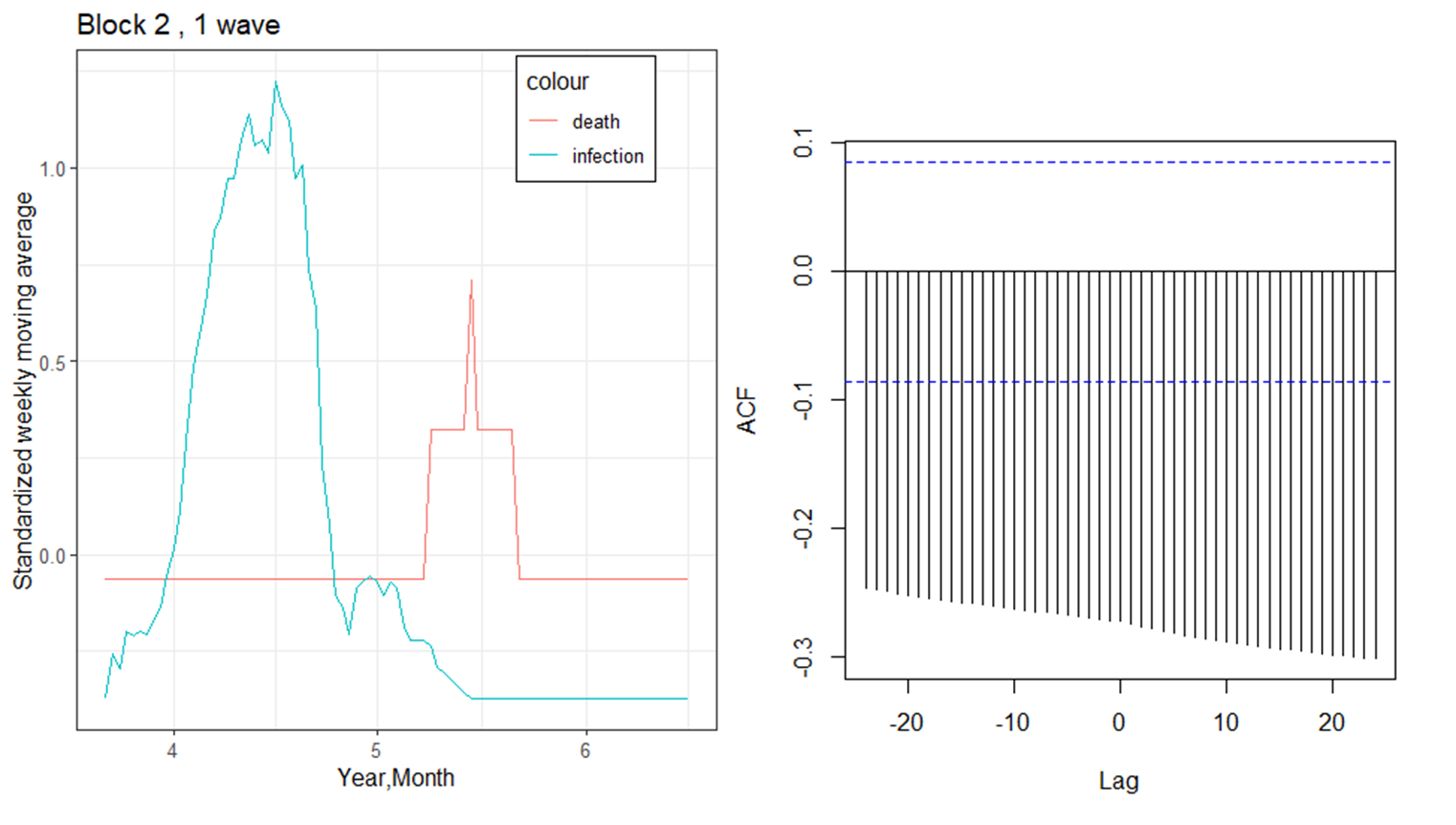


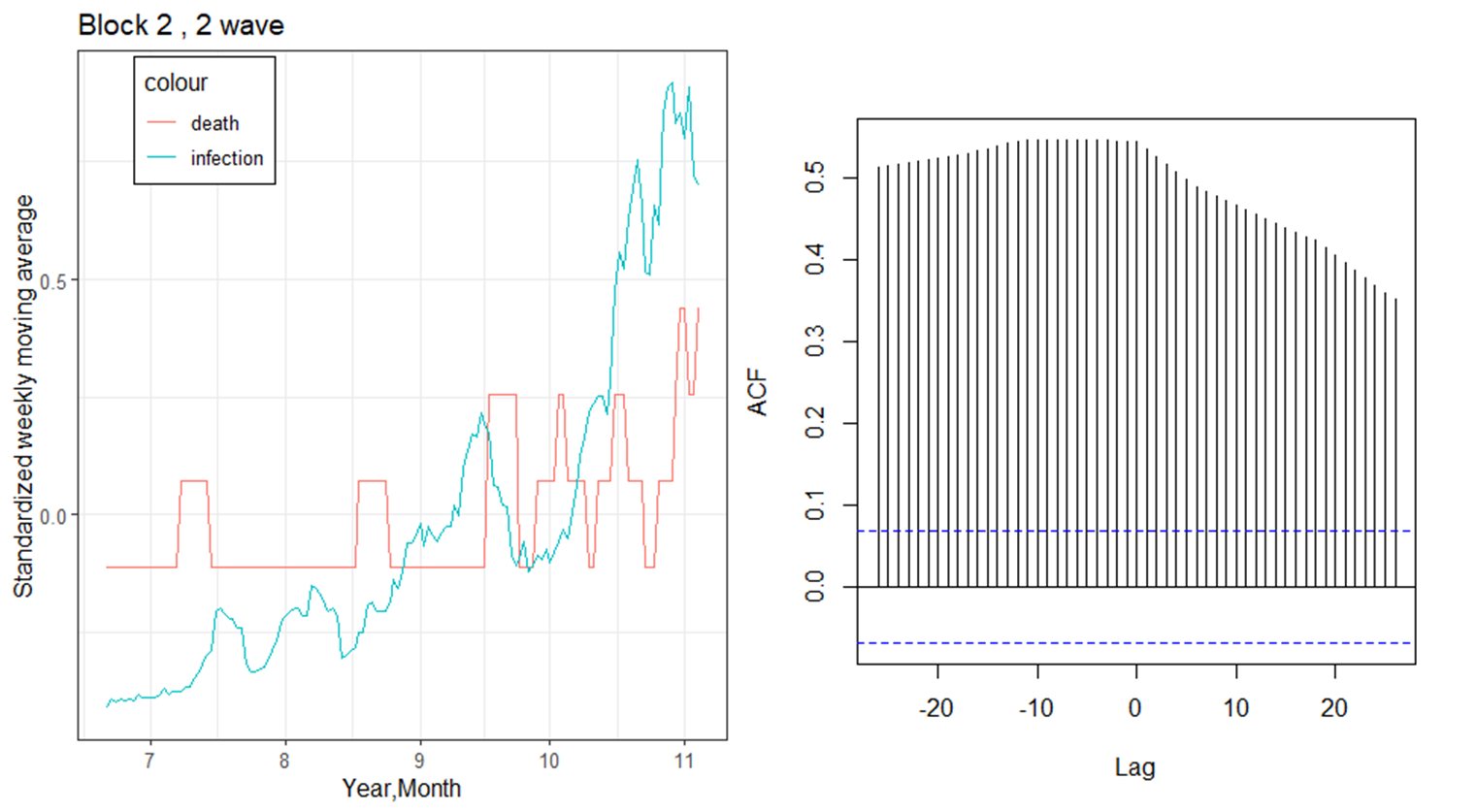


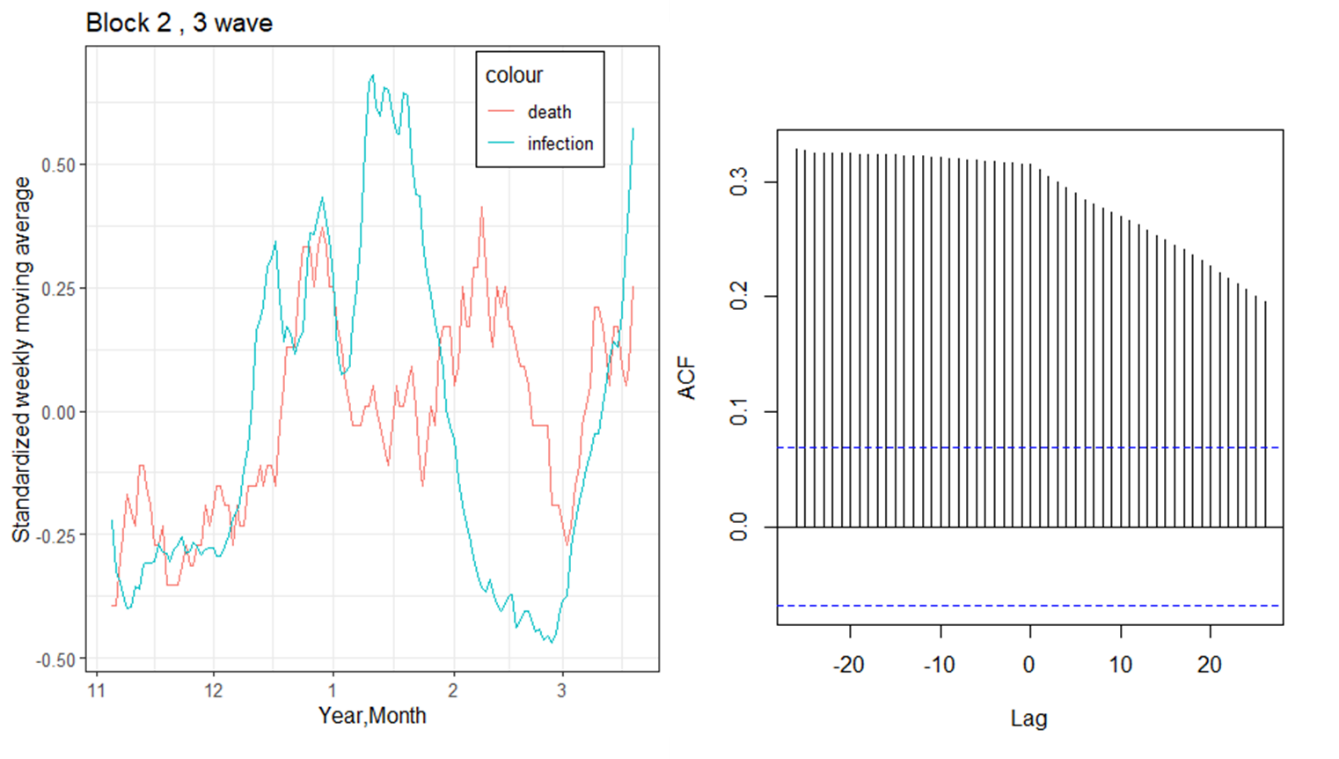


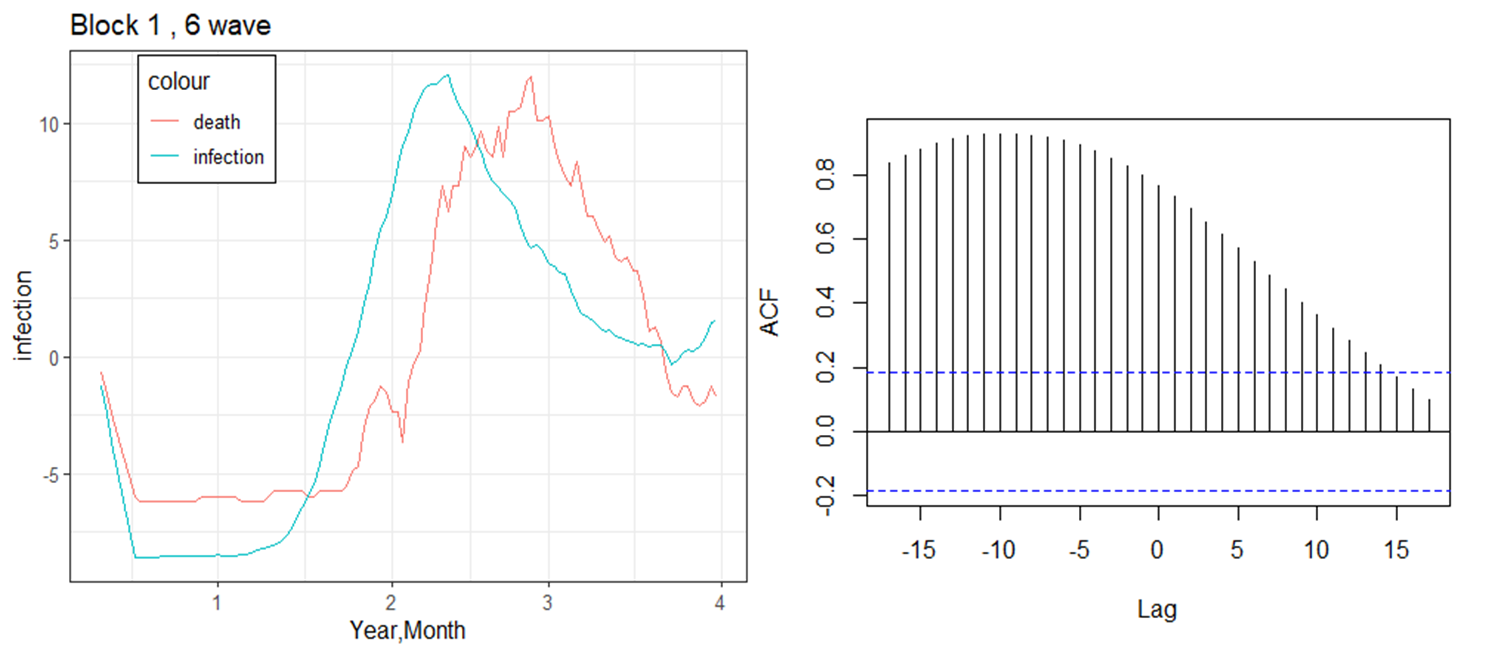


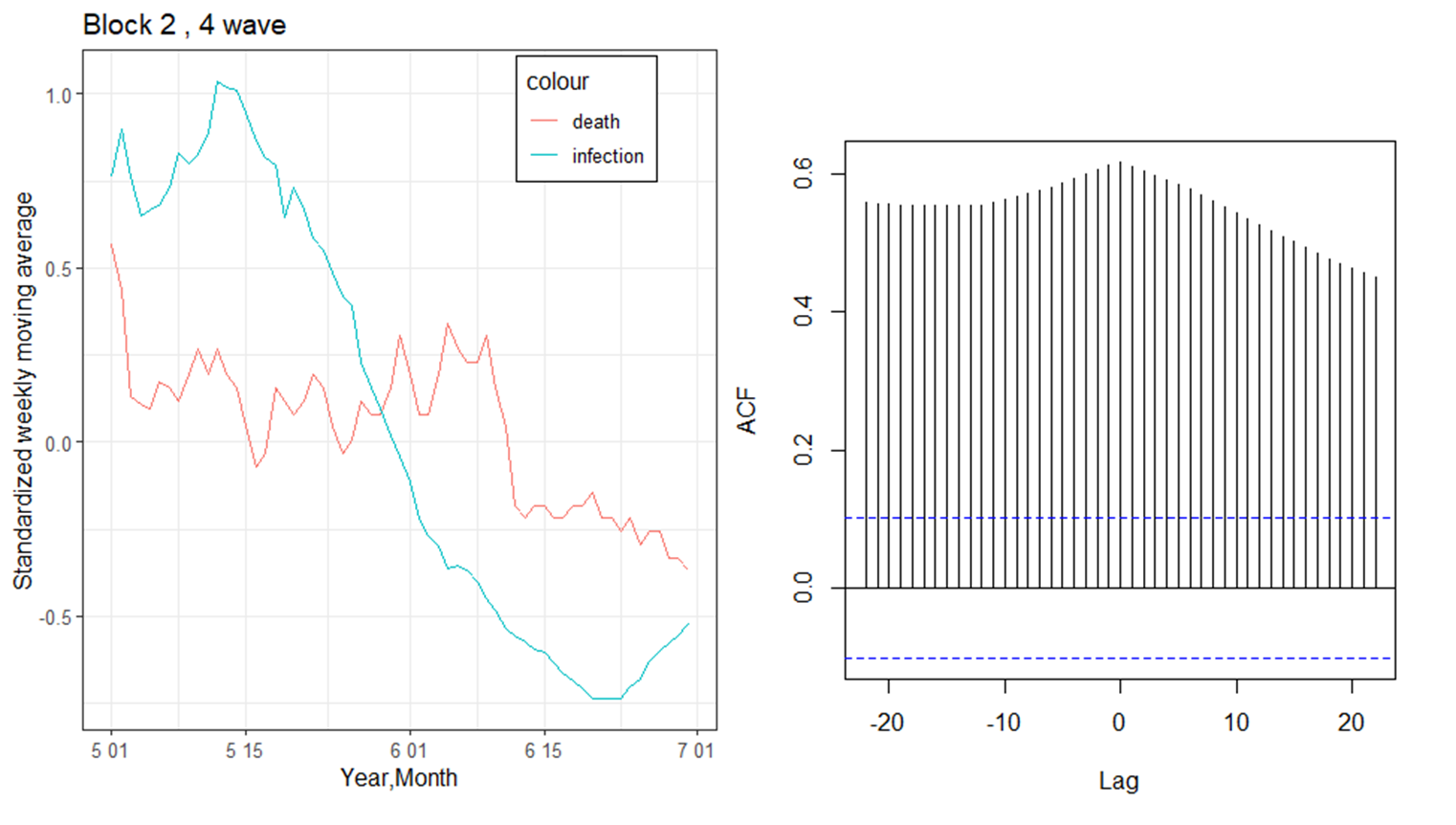


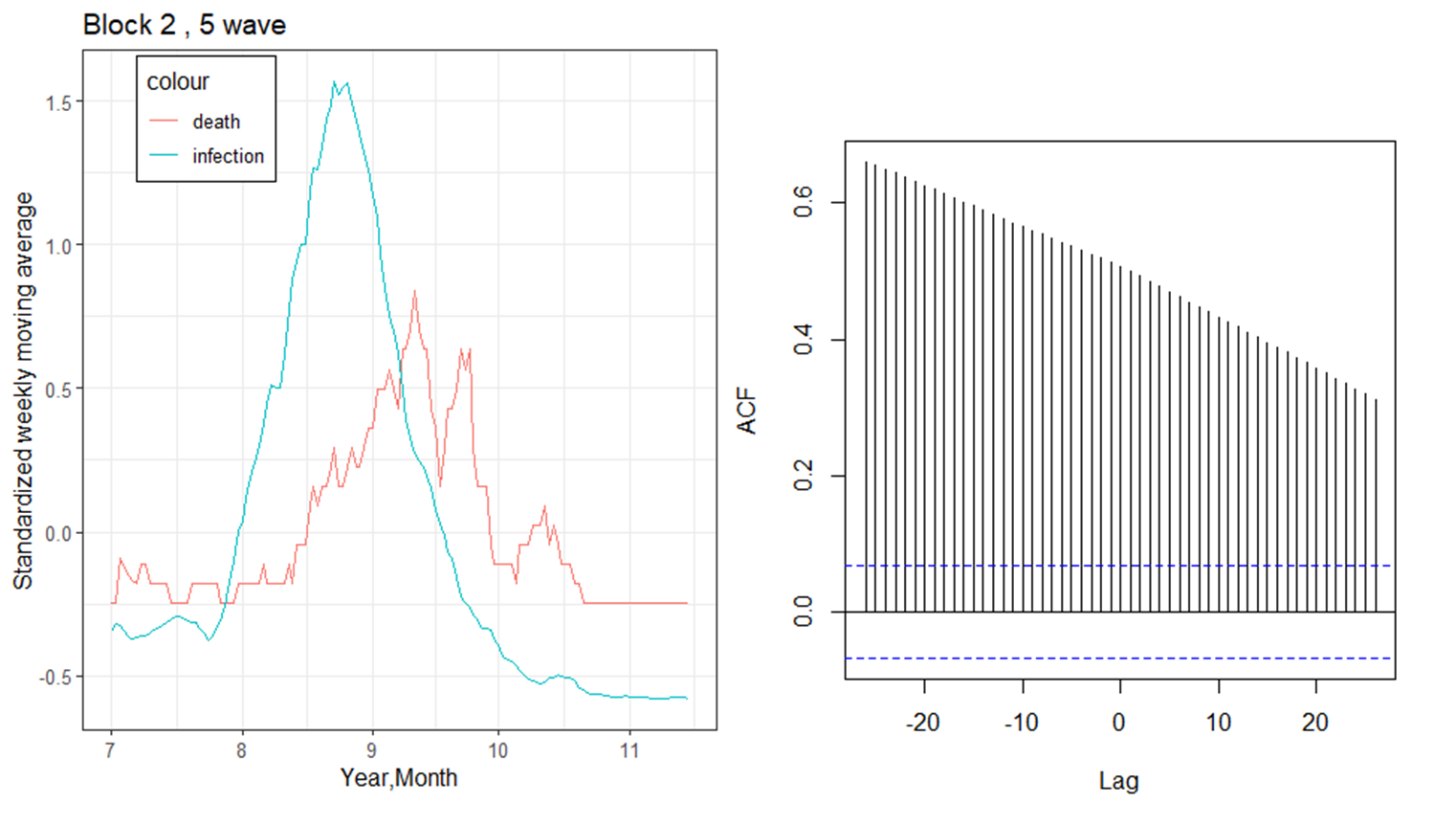


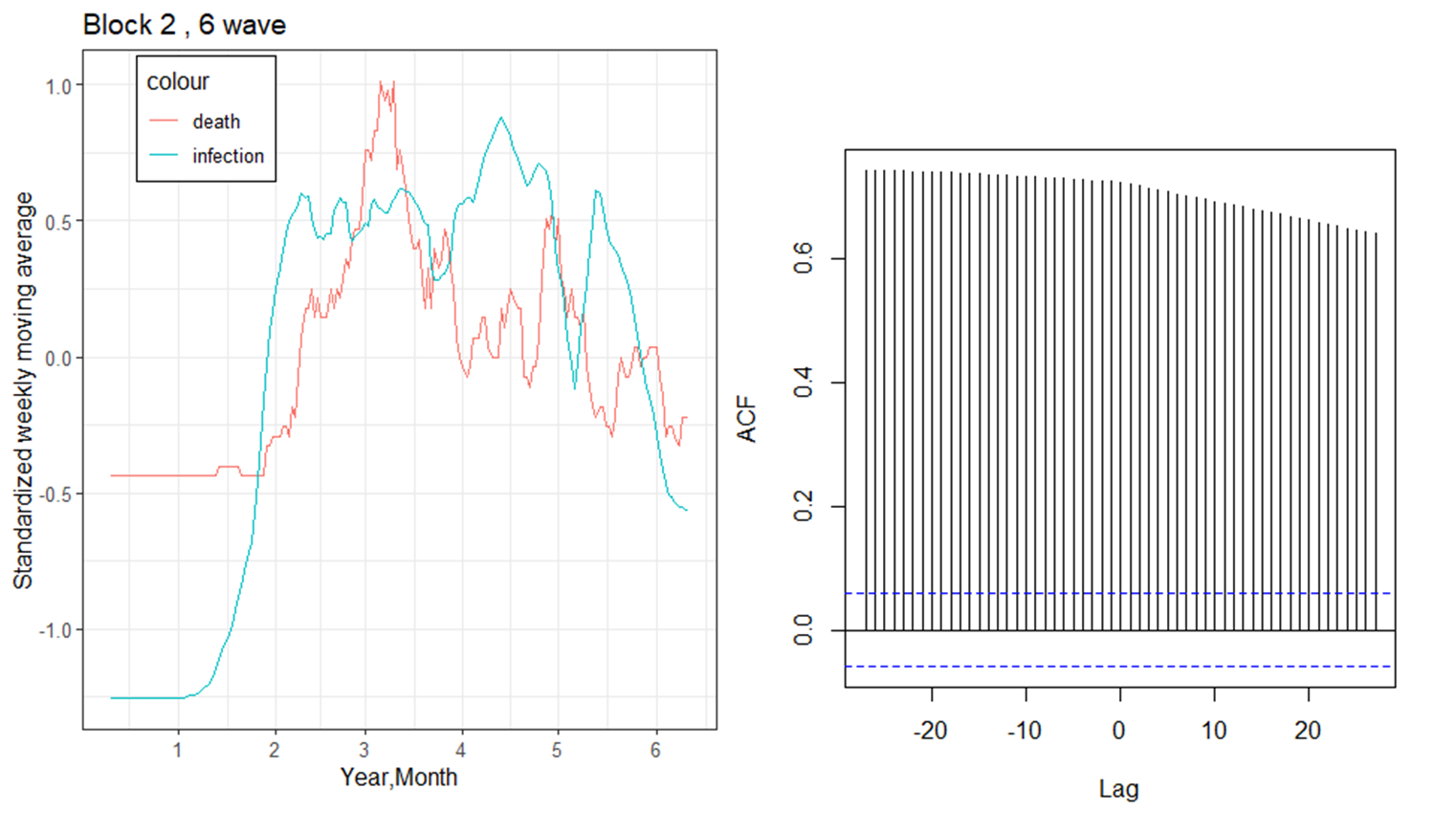


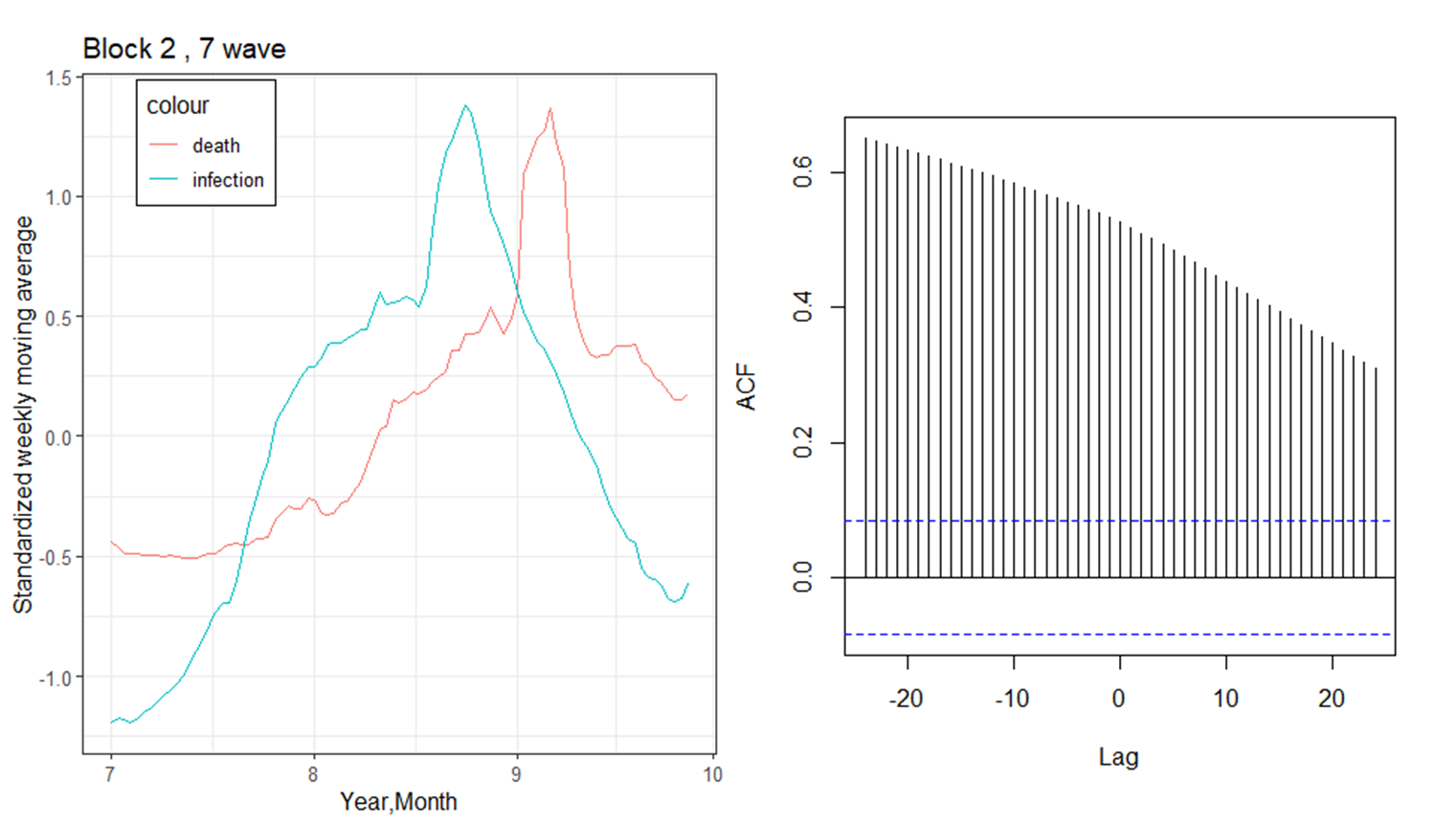


**(c)Kanto block**


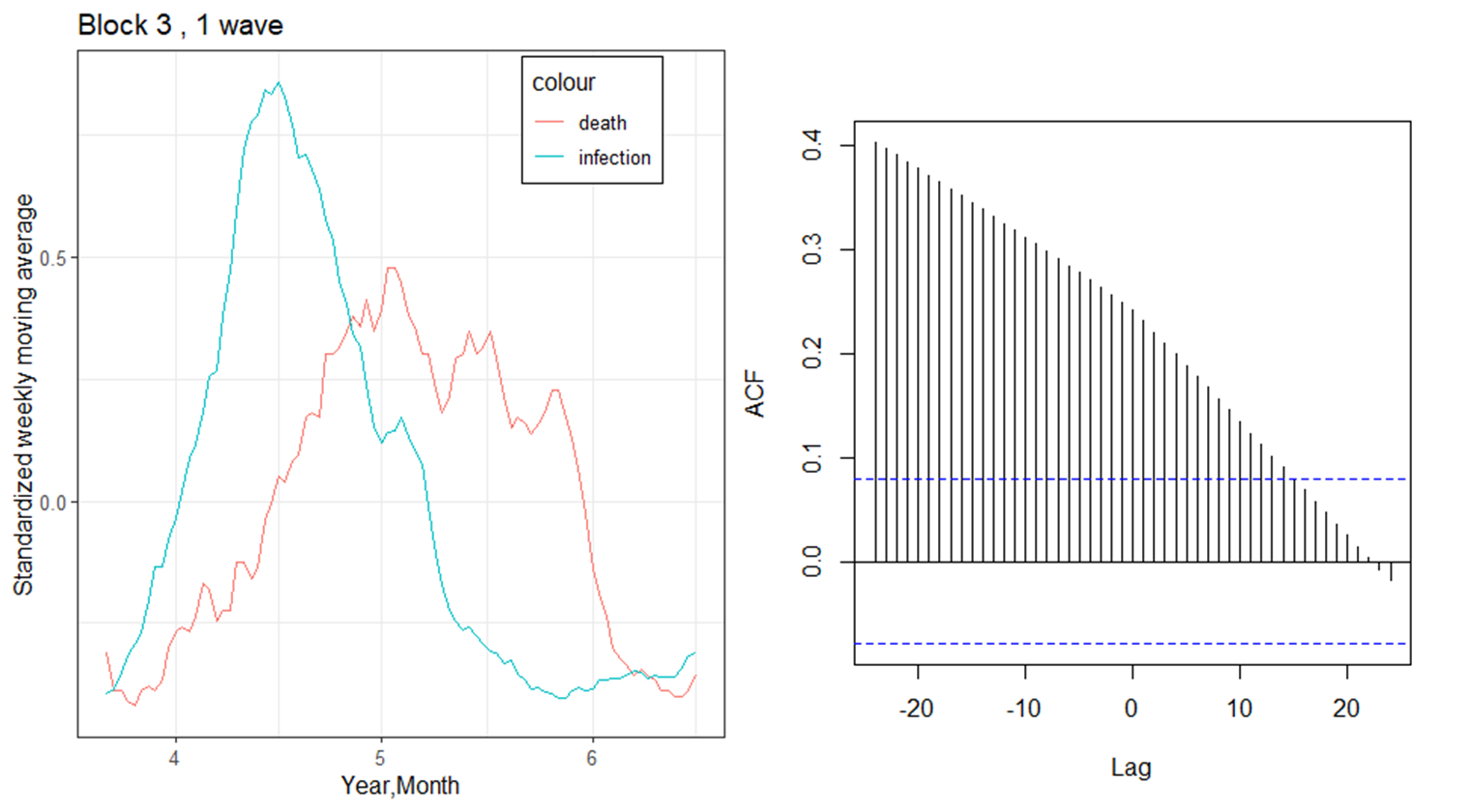


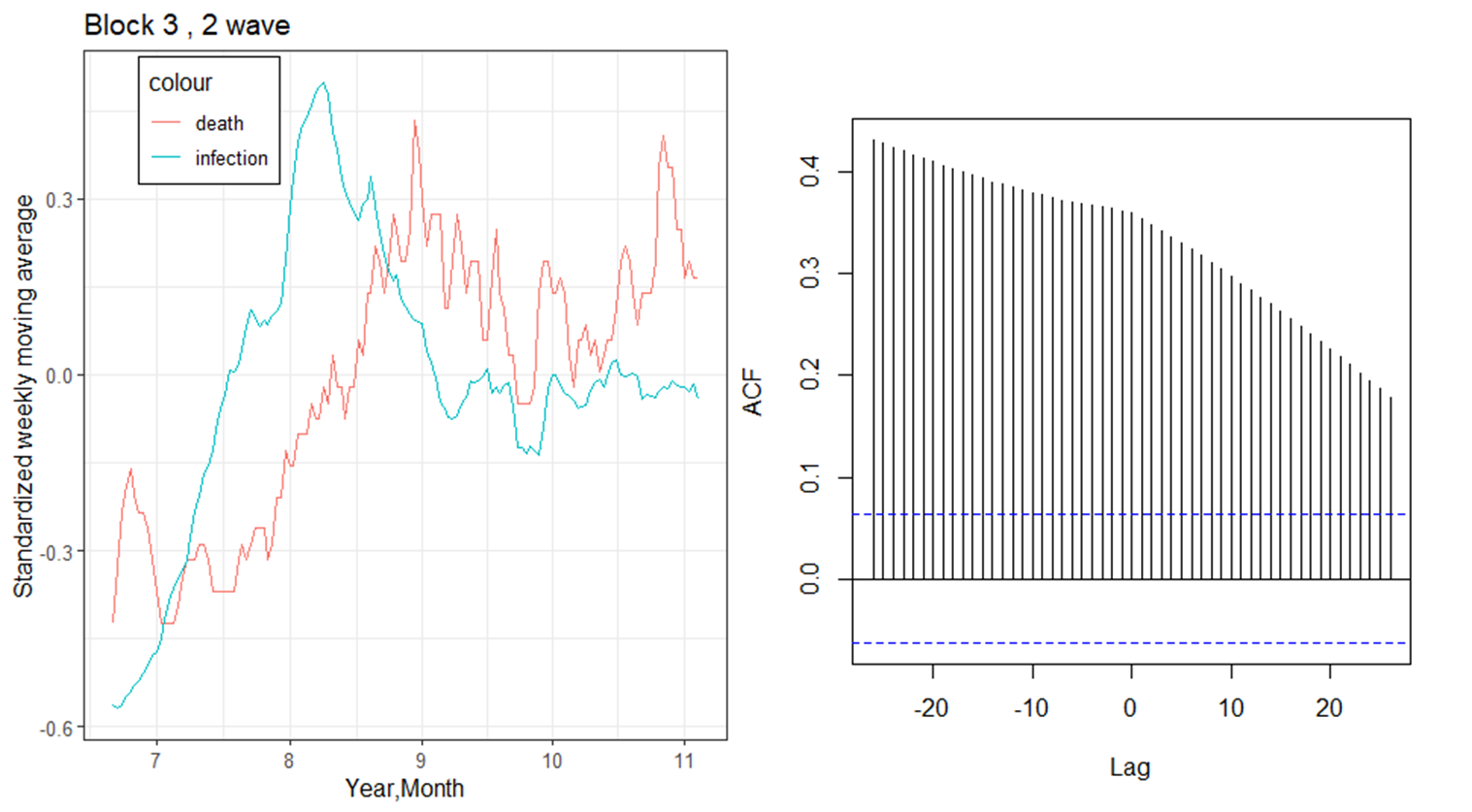


**
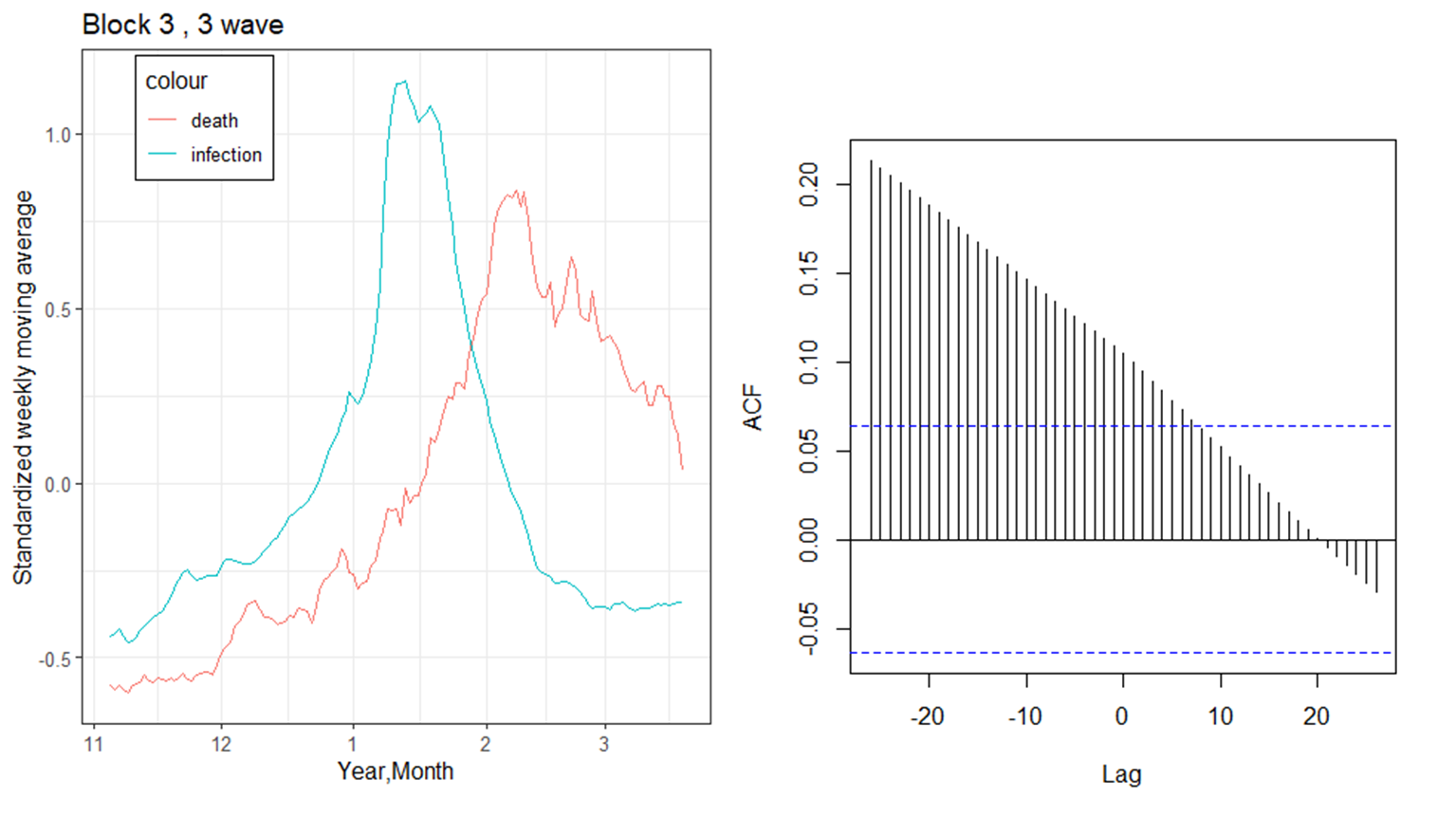
**

**
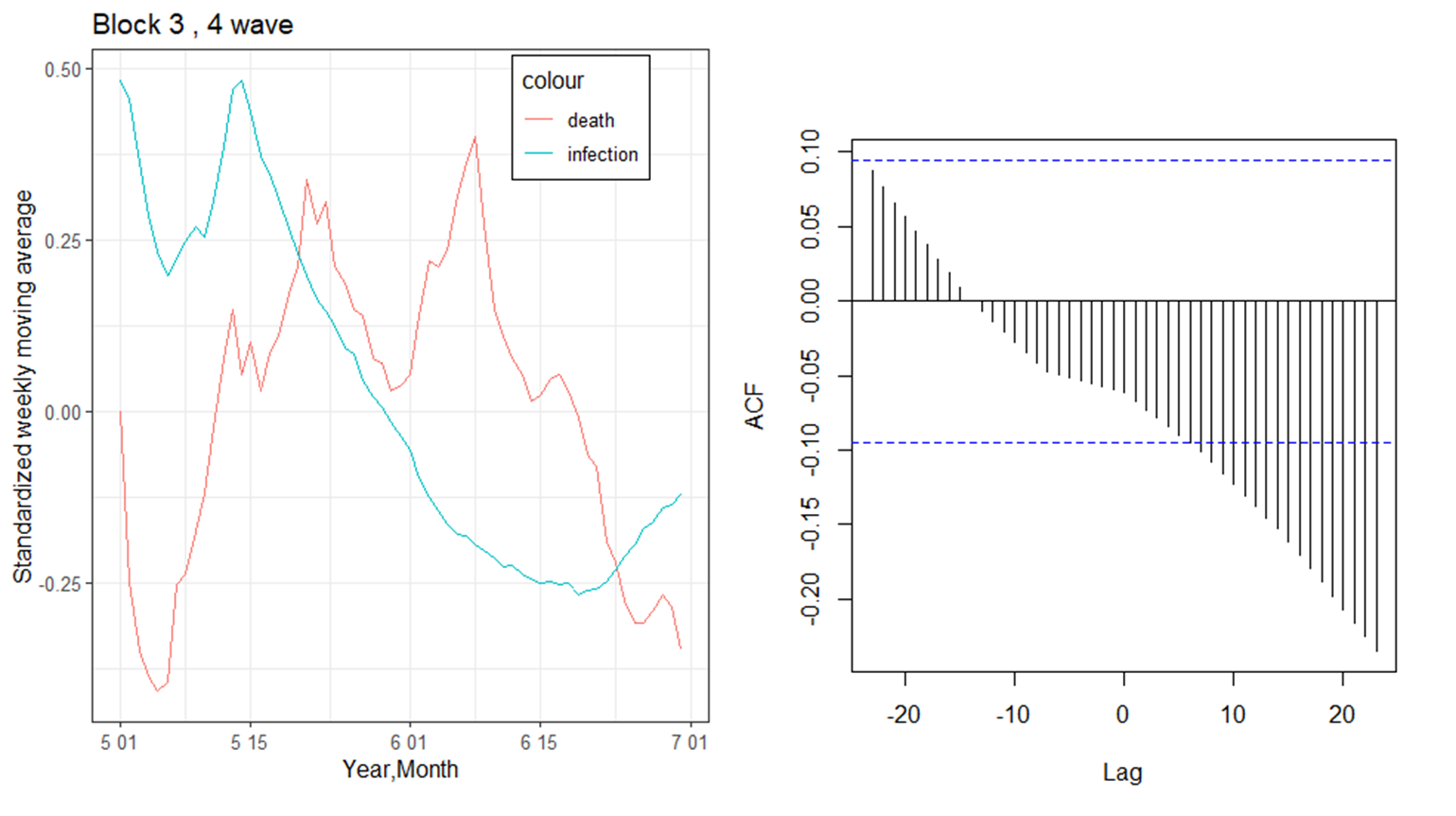
**

**
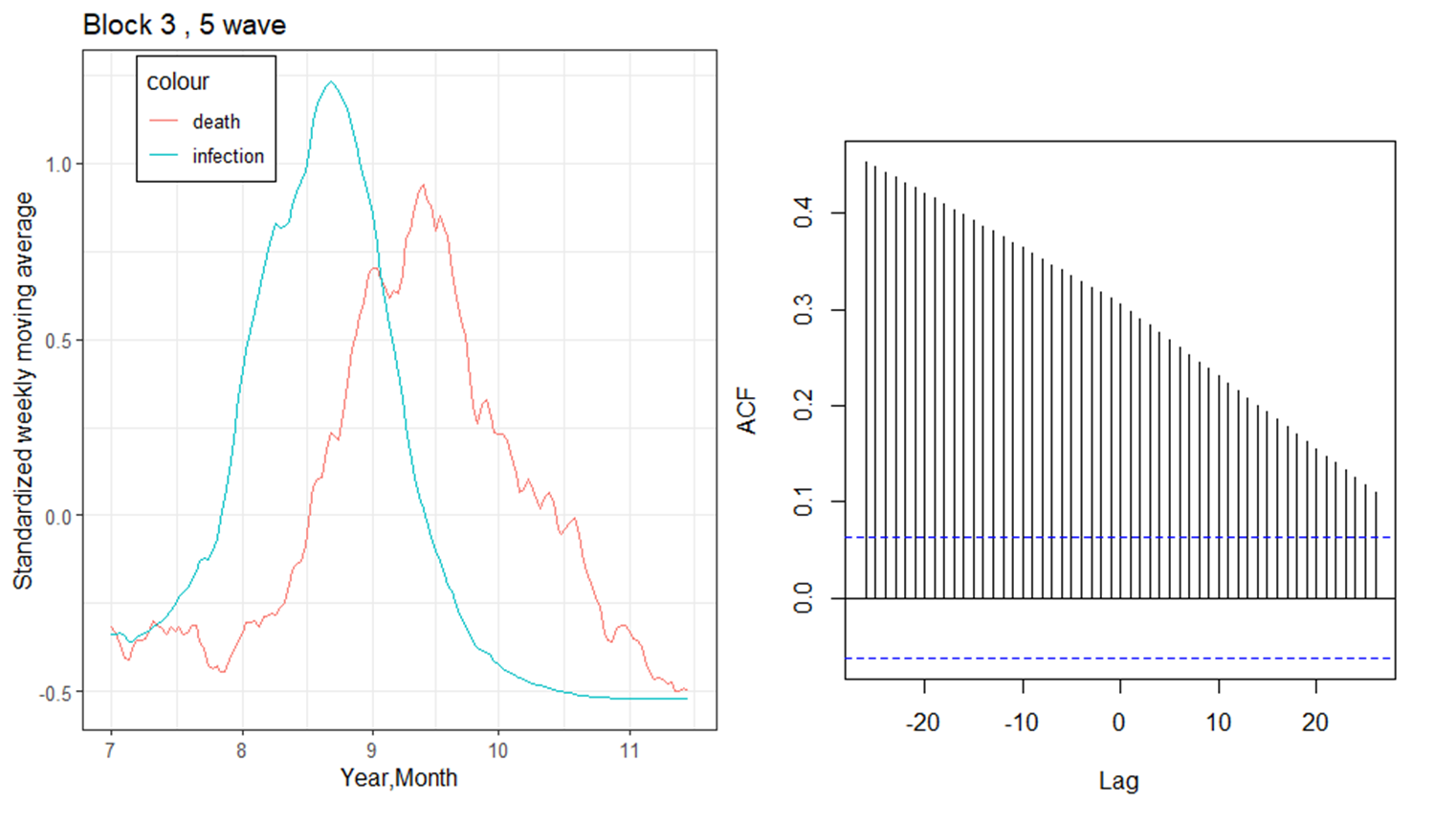
**

**
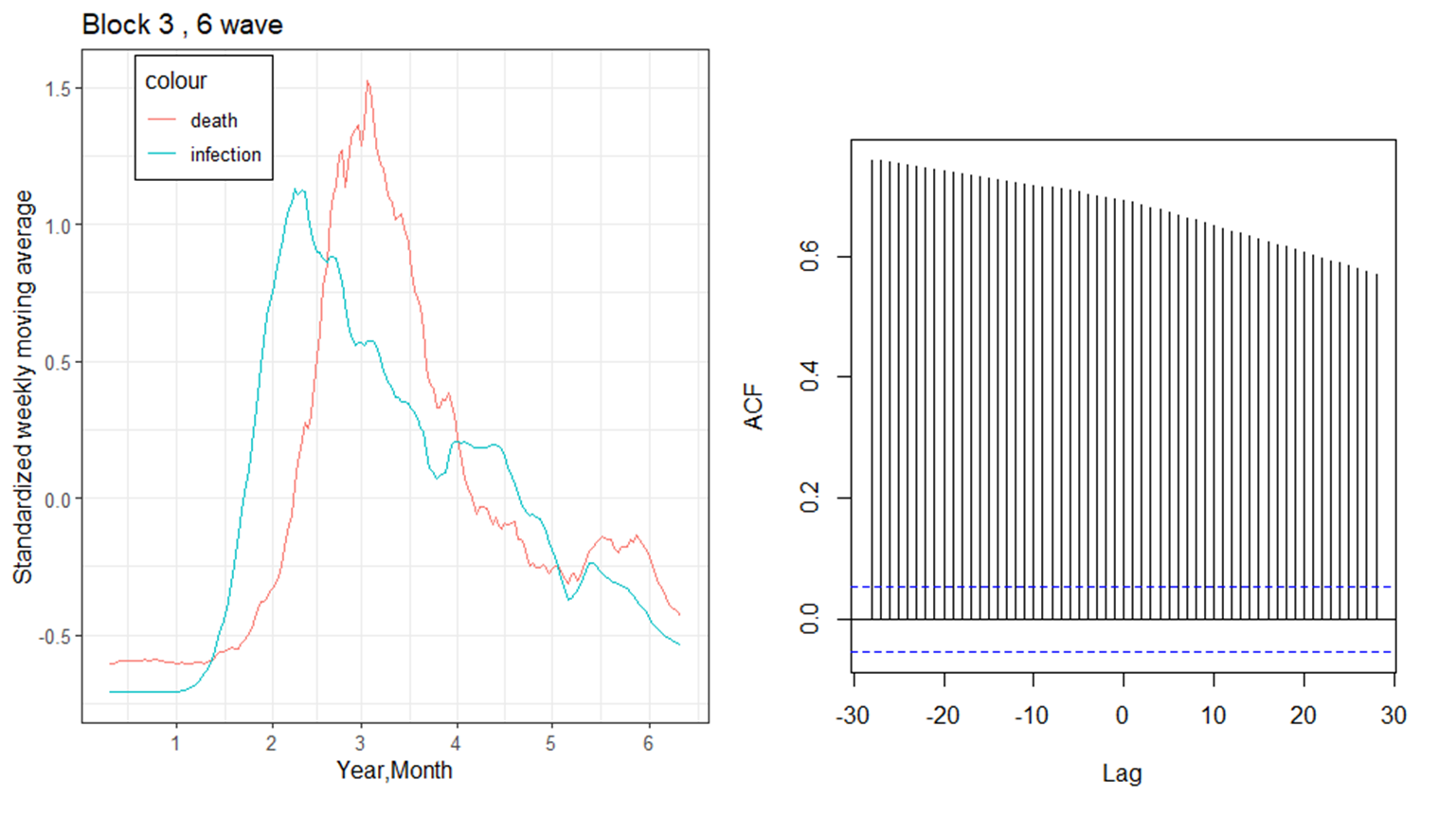
**

**
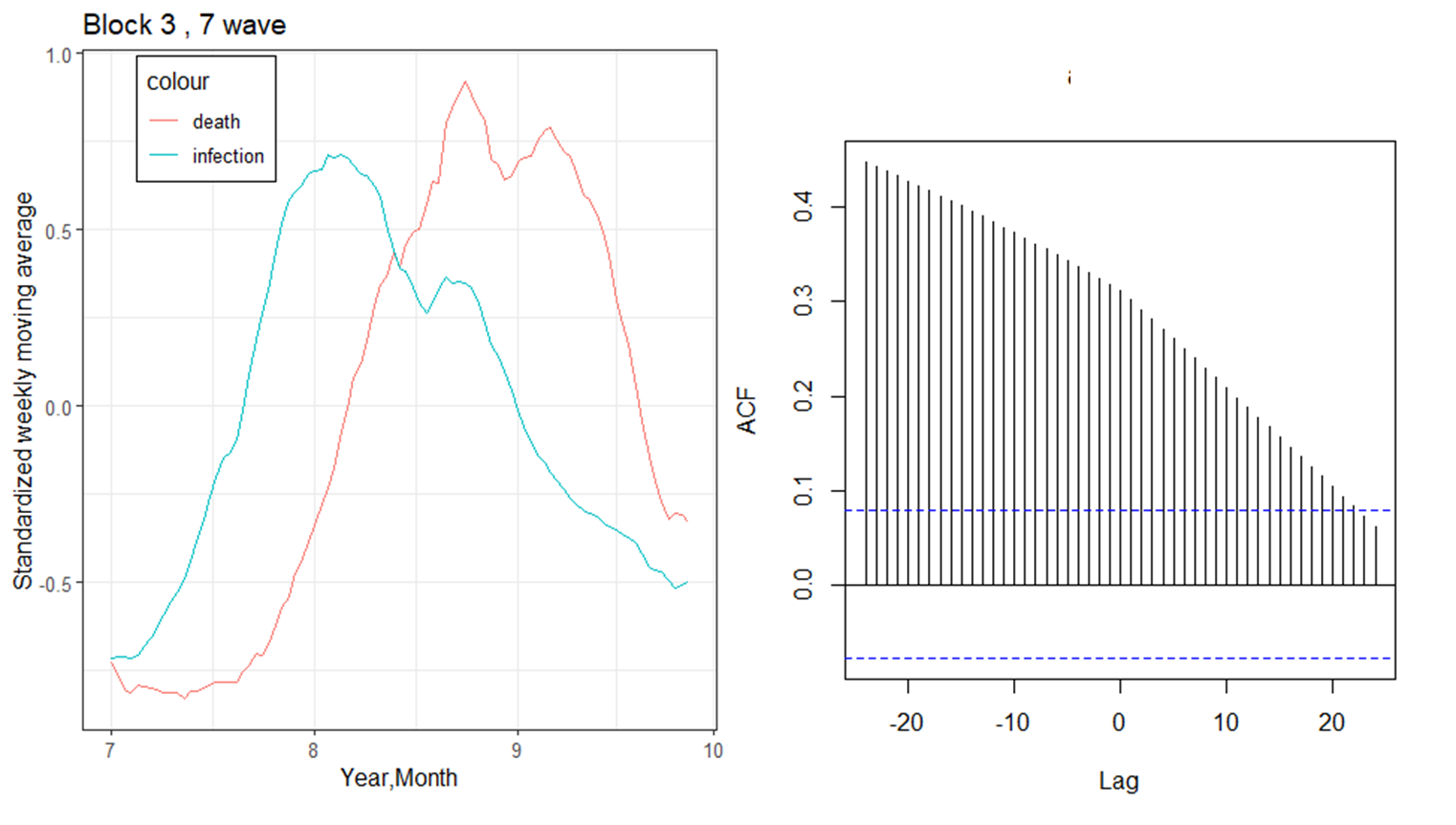
**

**(d)Chubo block**


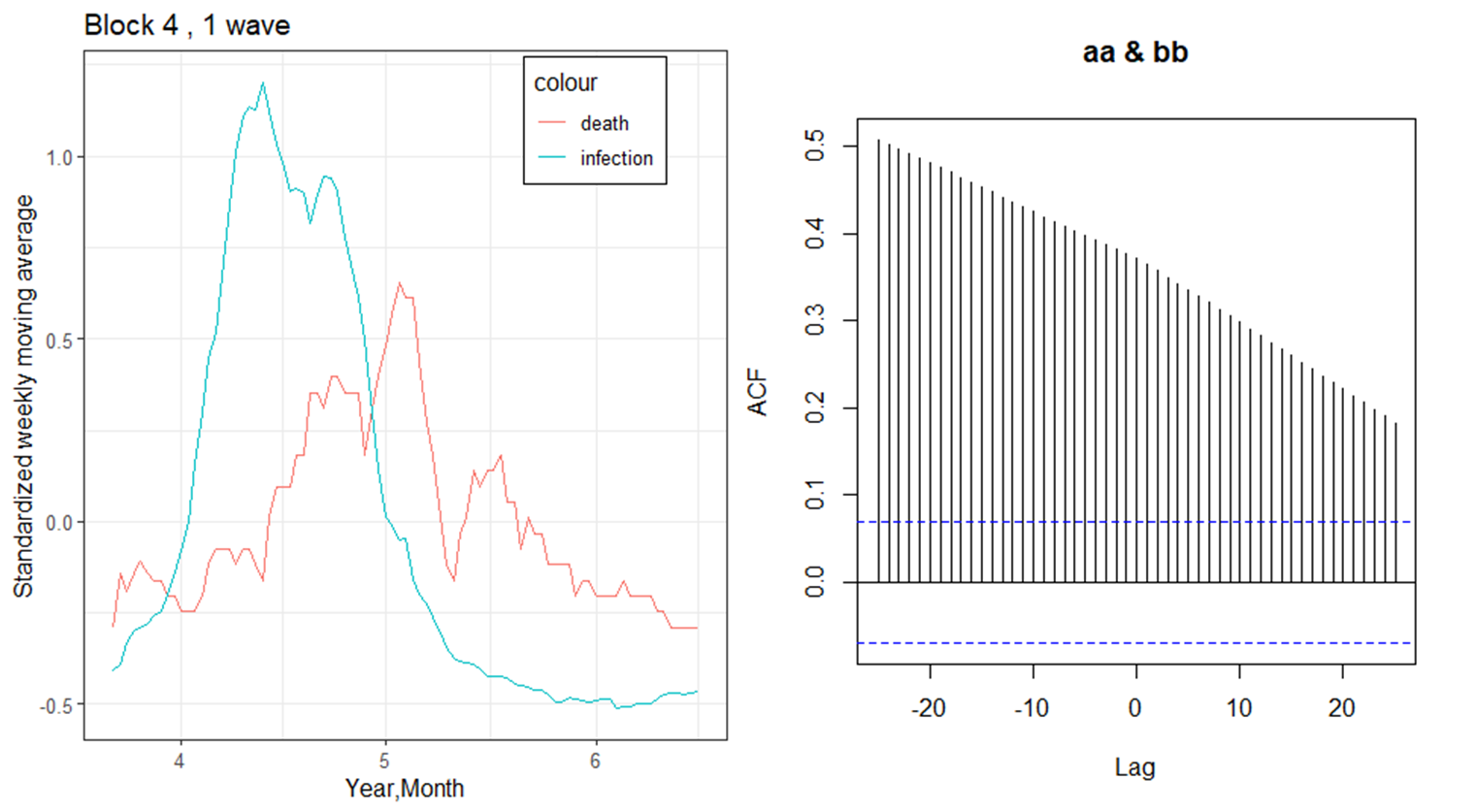


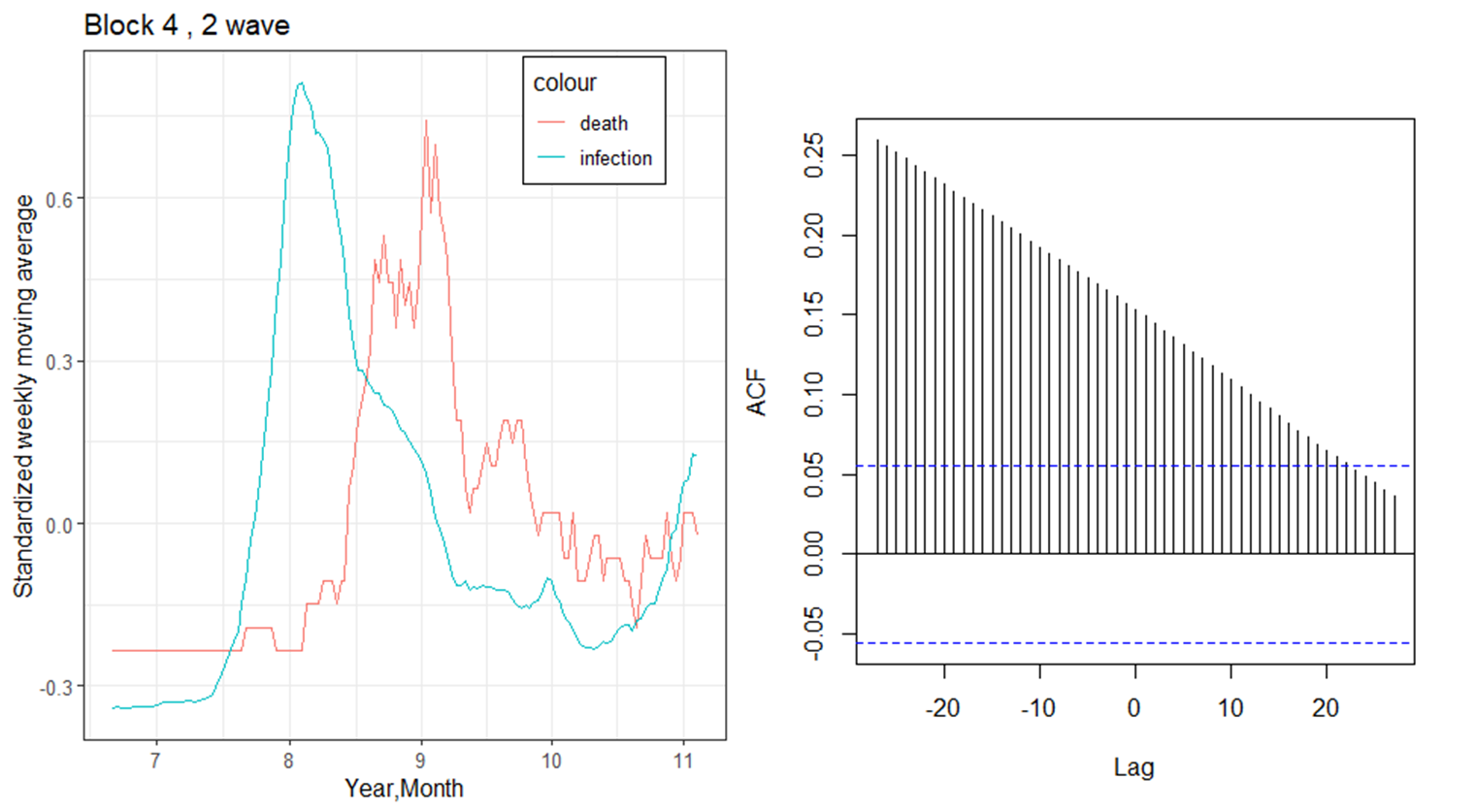


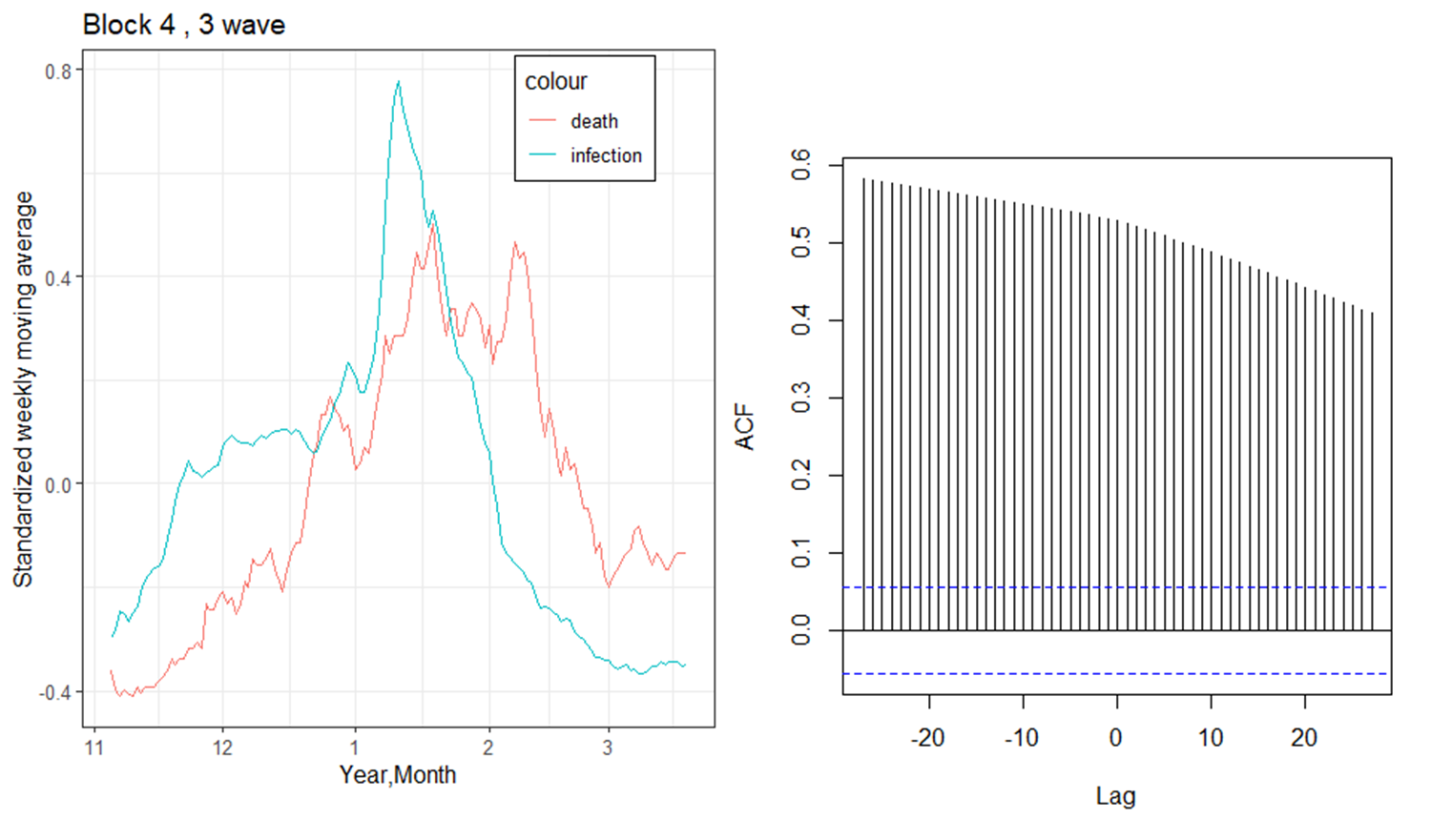


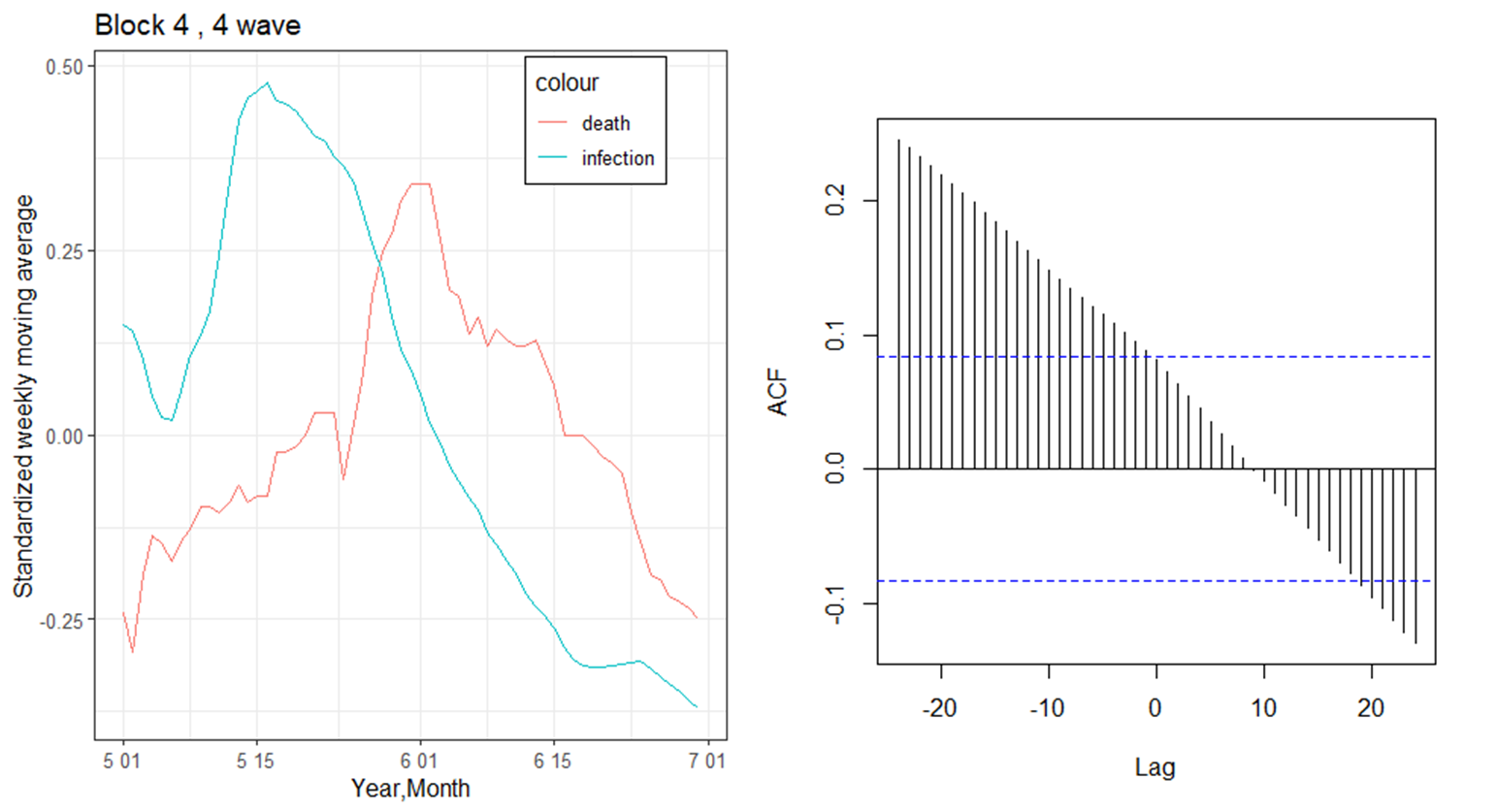


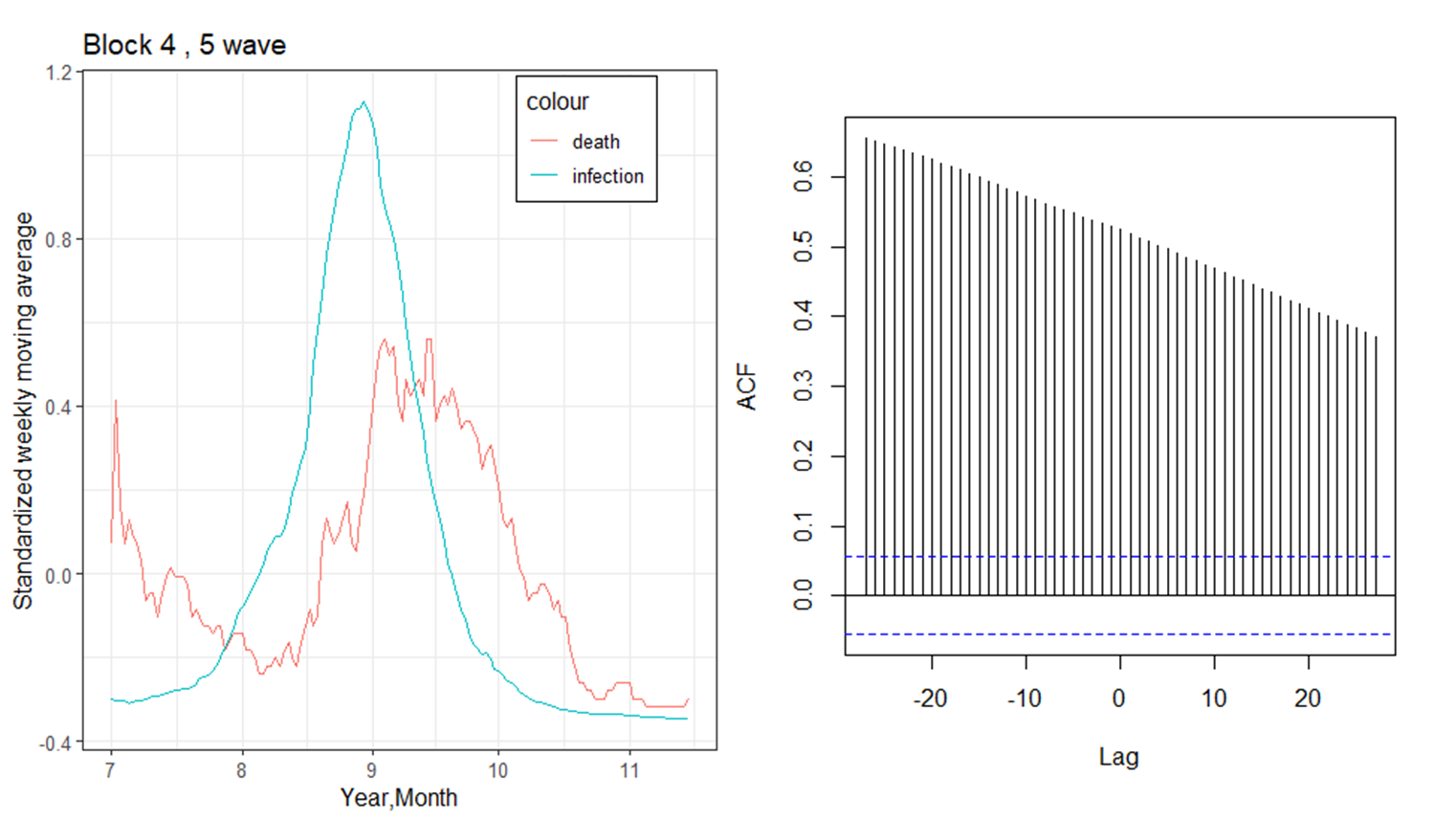


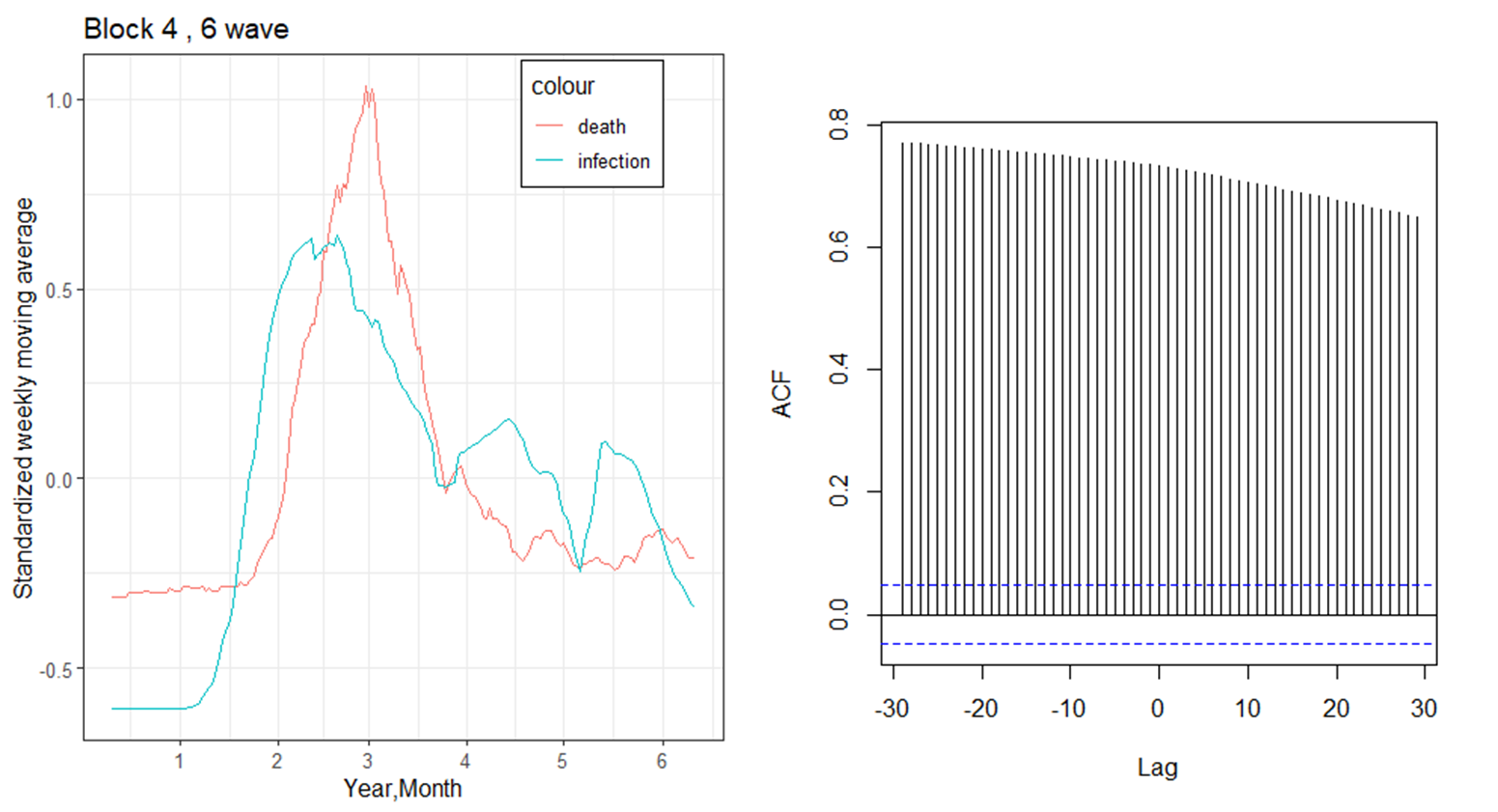


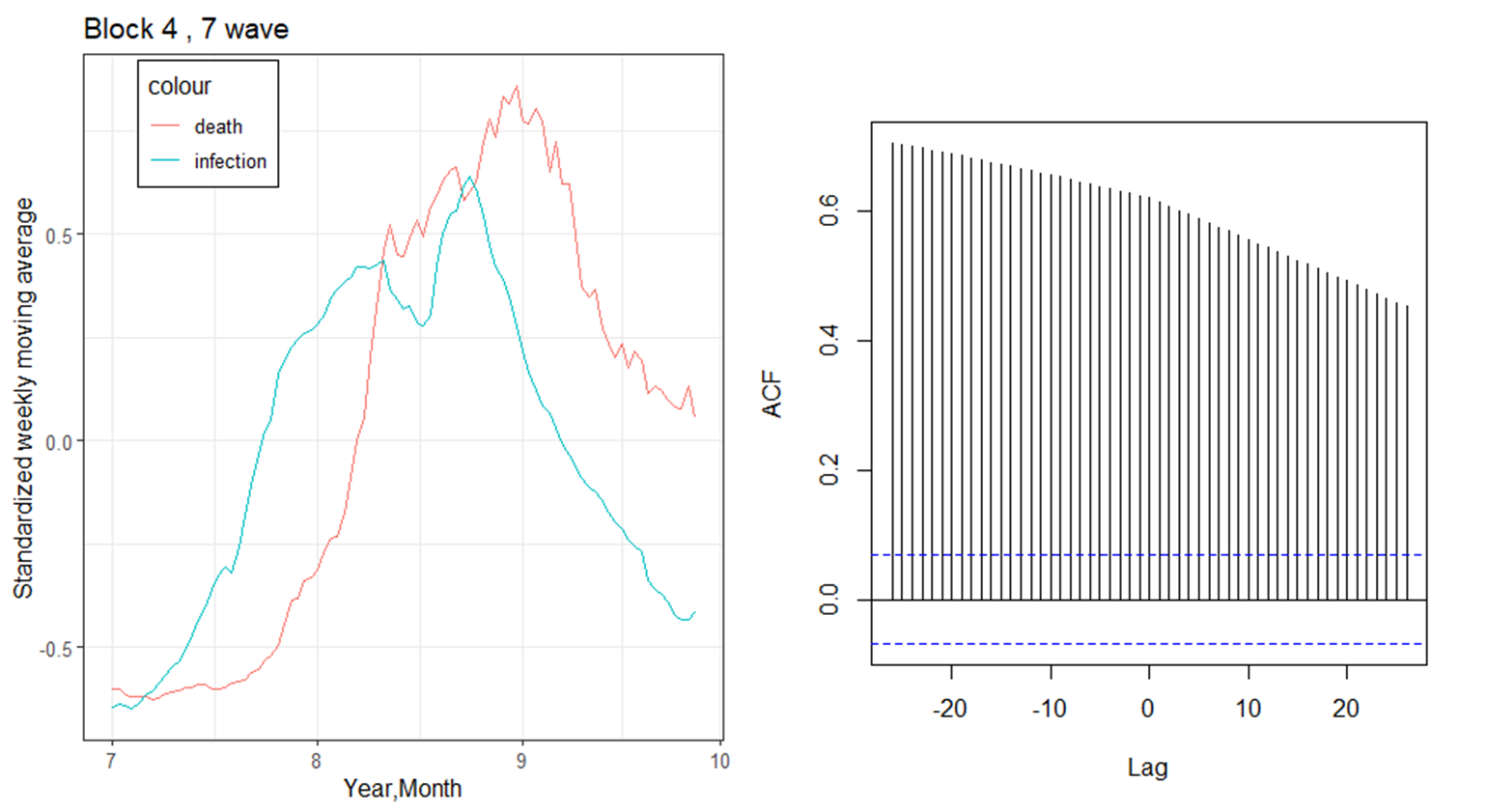


**(e) Kinki block**

**
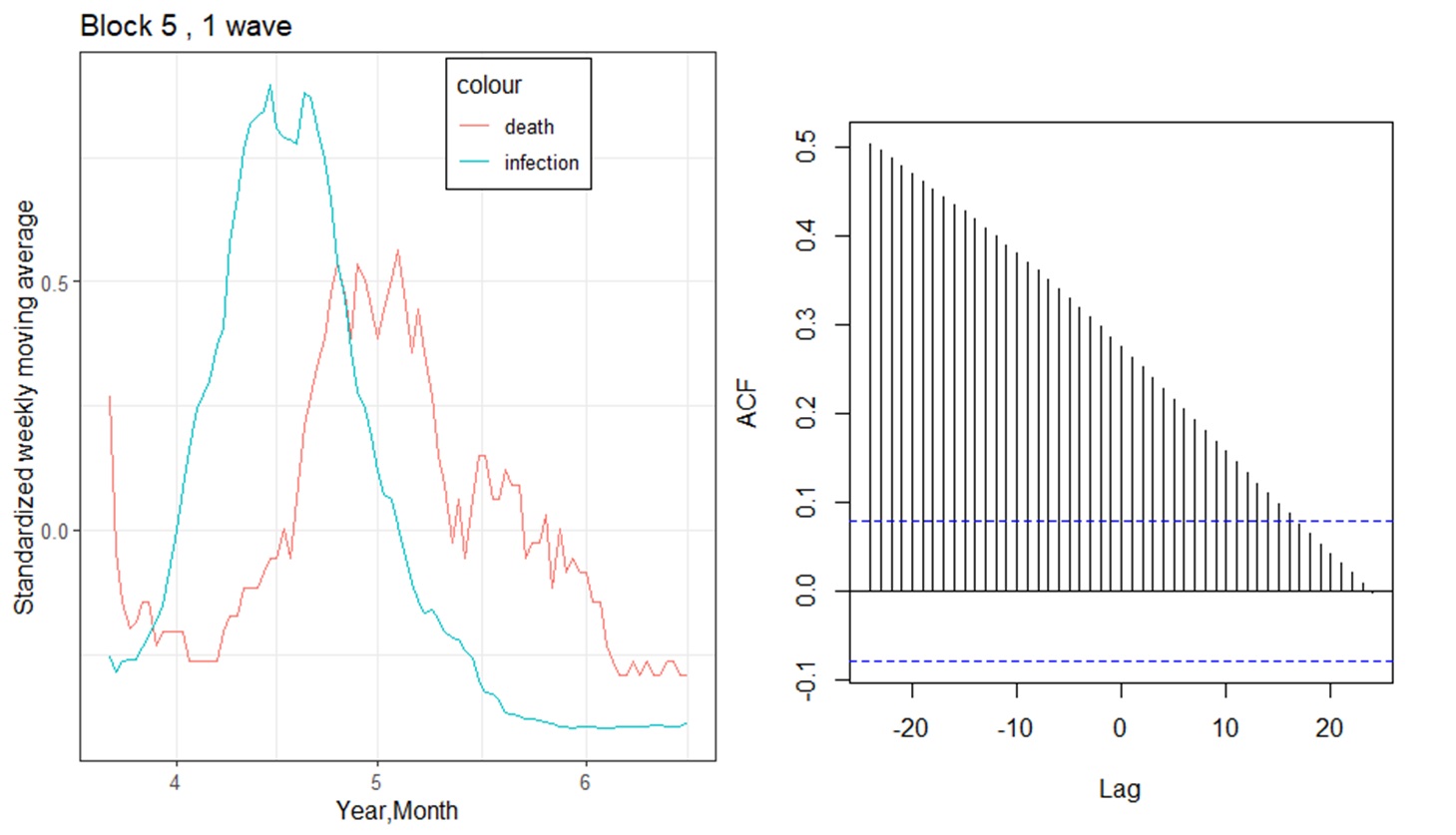
**

**
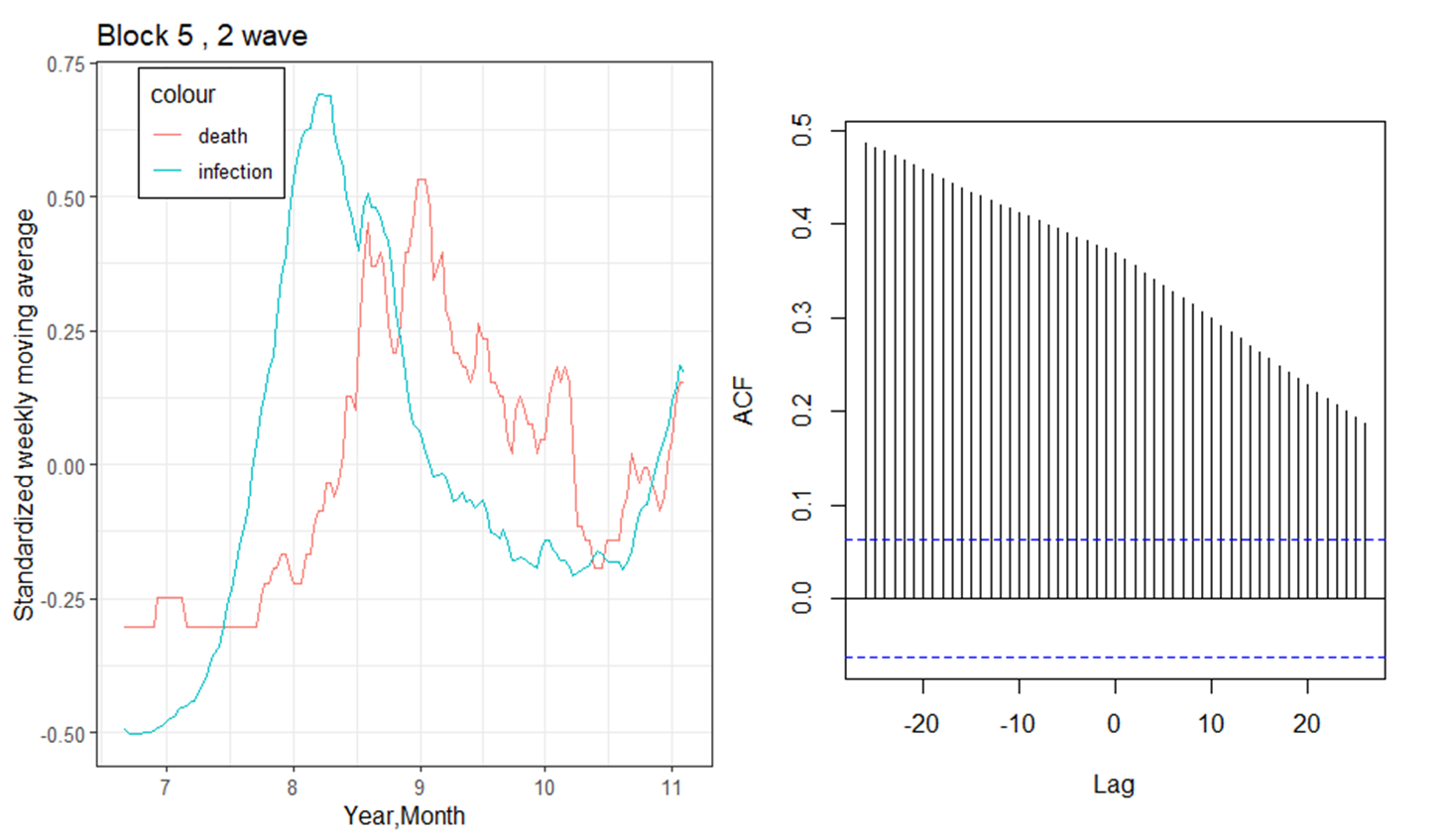
**

**
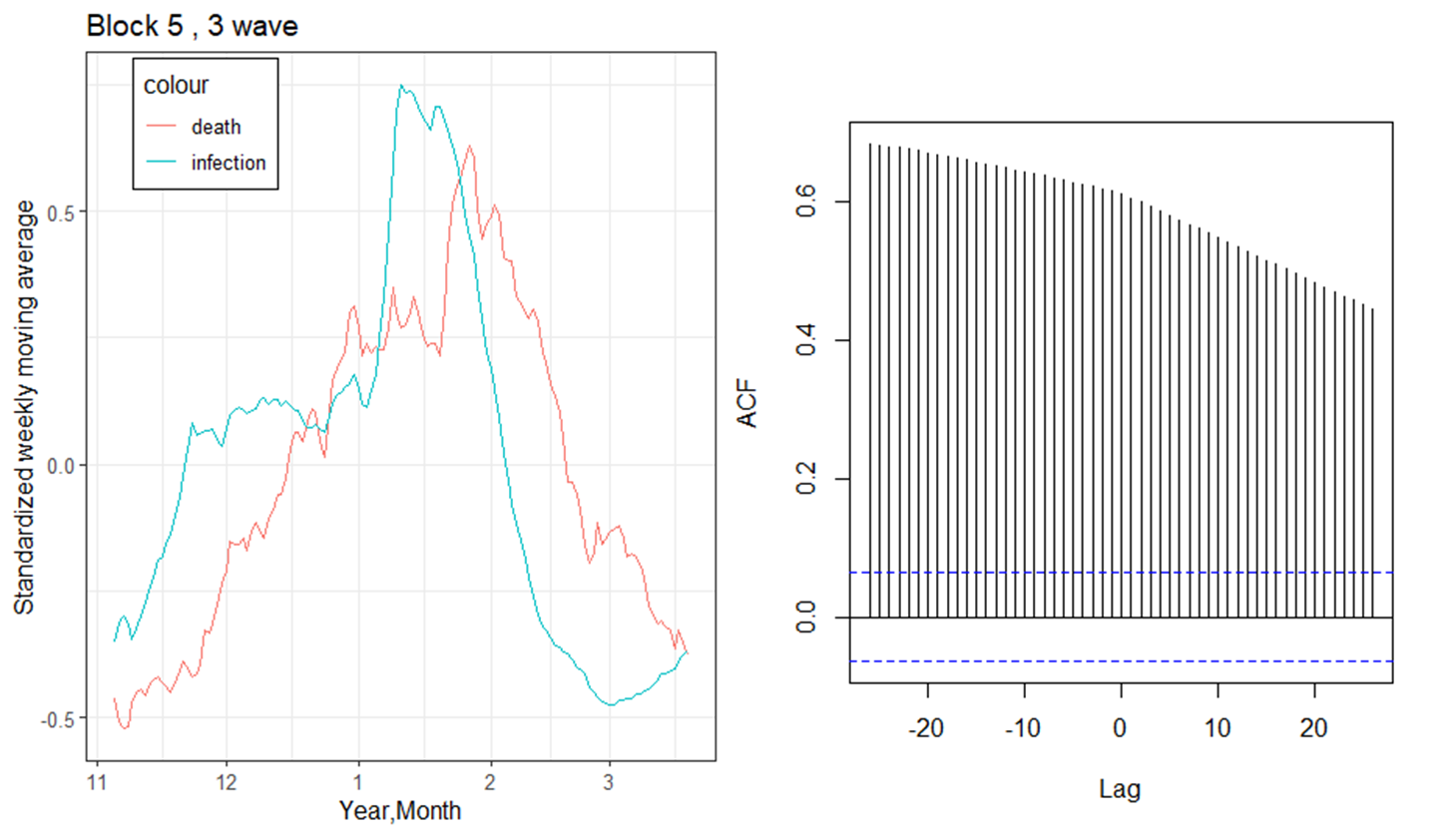
**

**
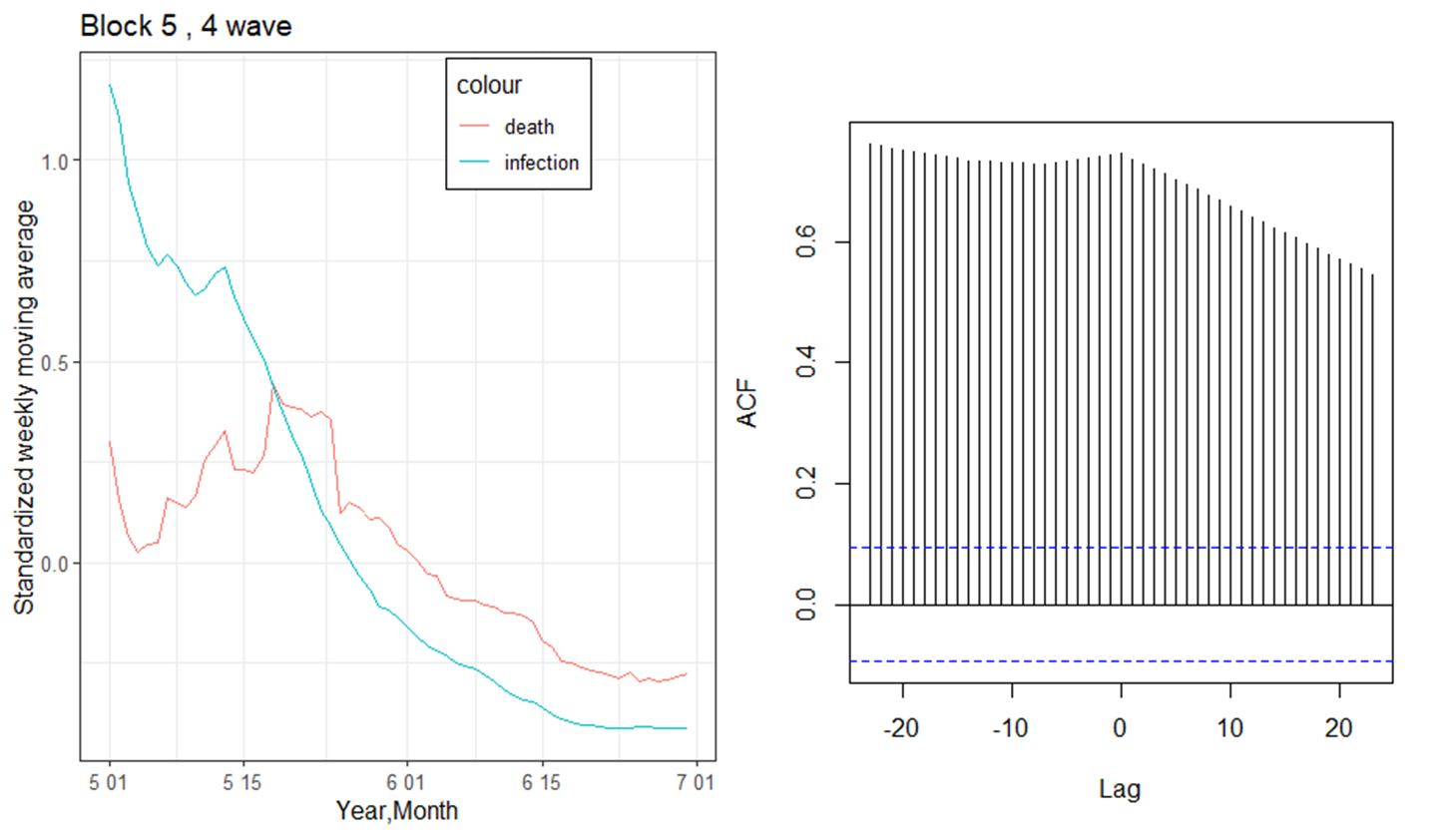
**

**
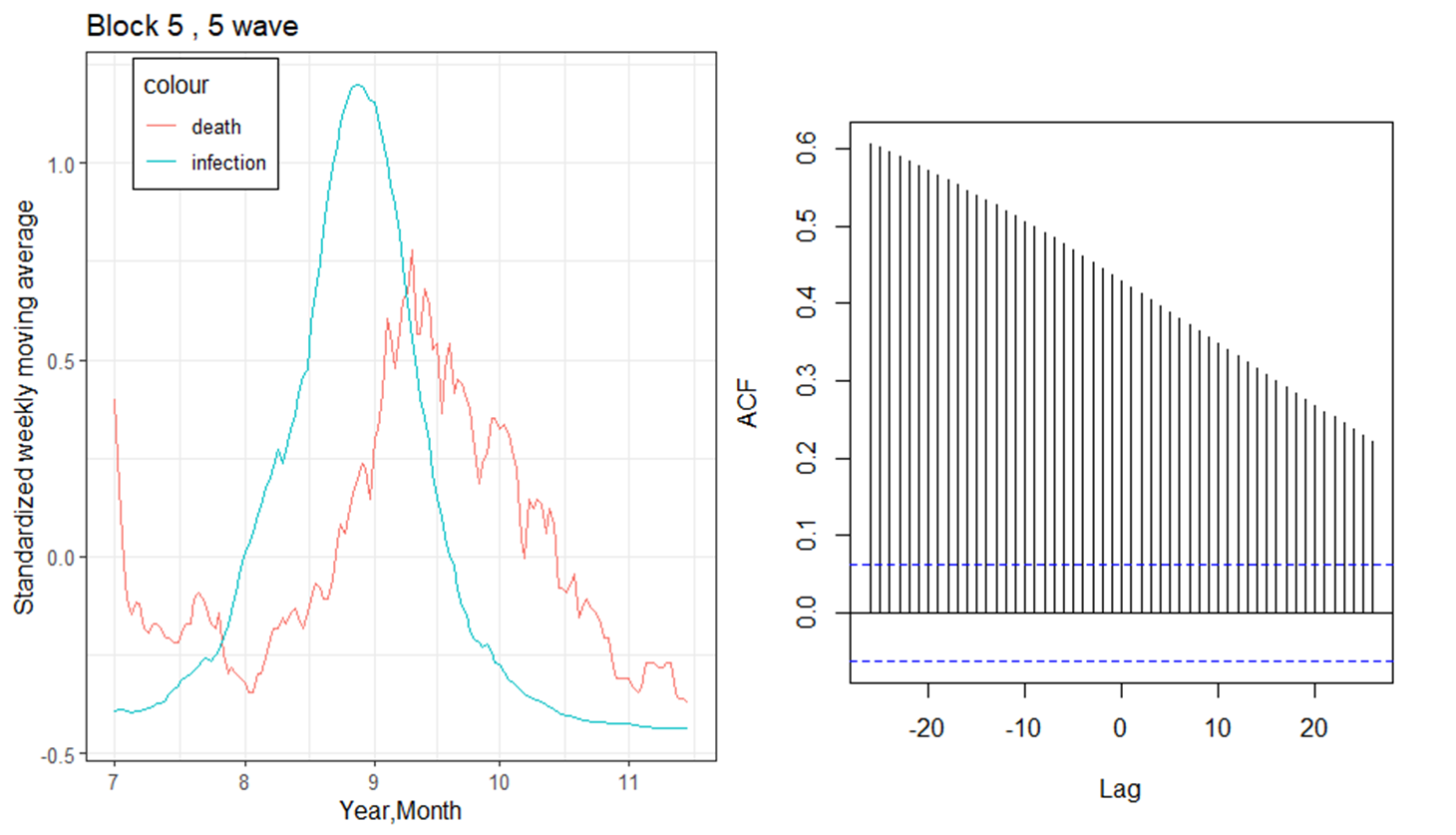
**

**
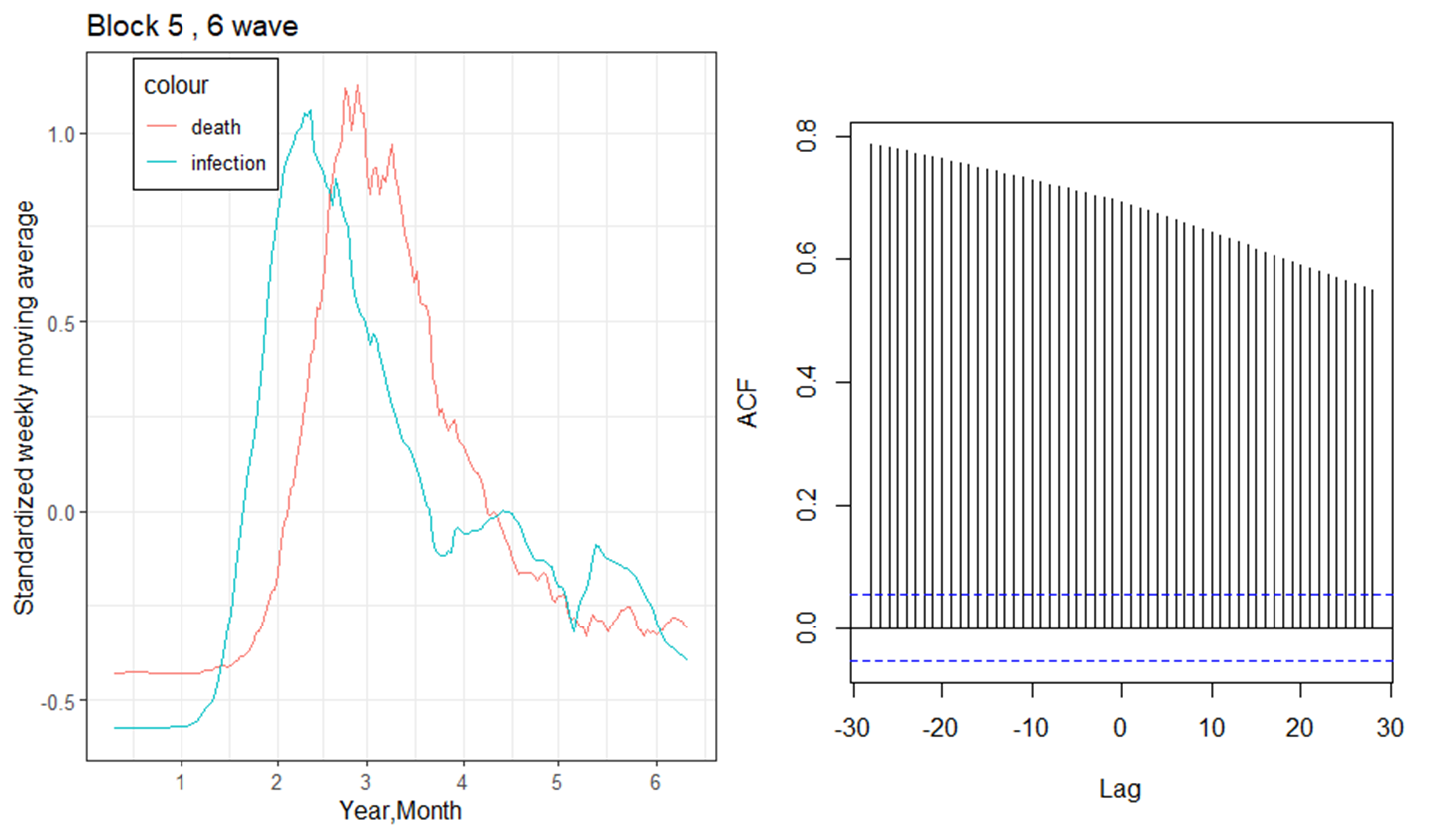
**

**
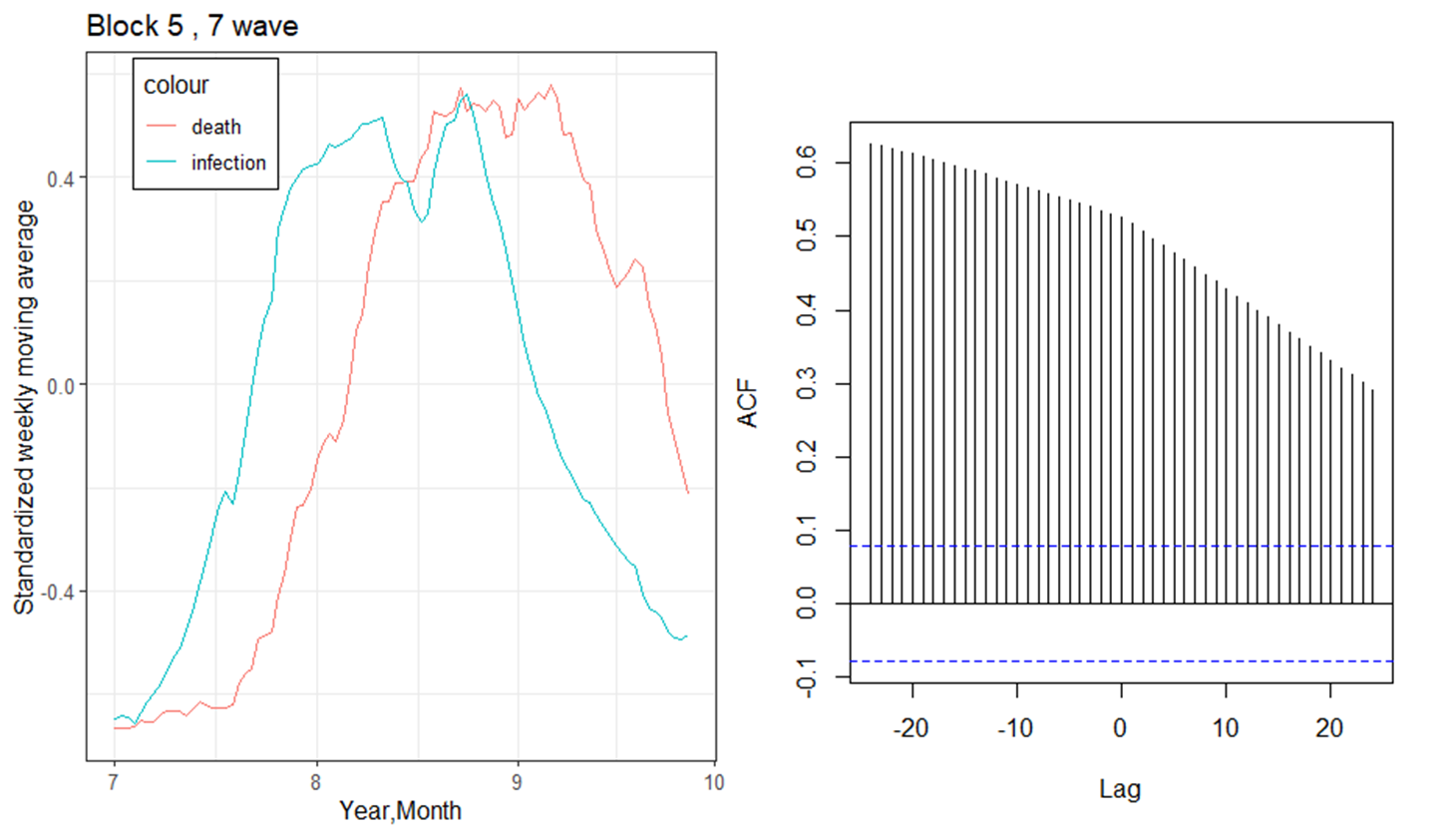
**

**(f) Chugoku block**


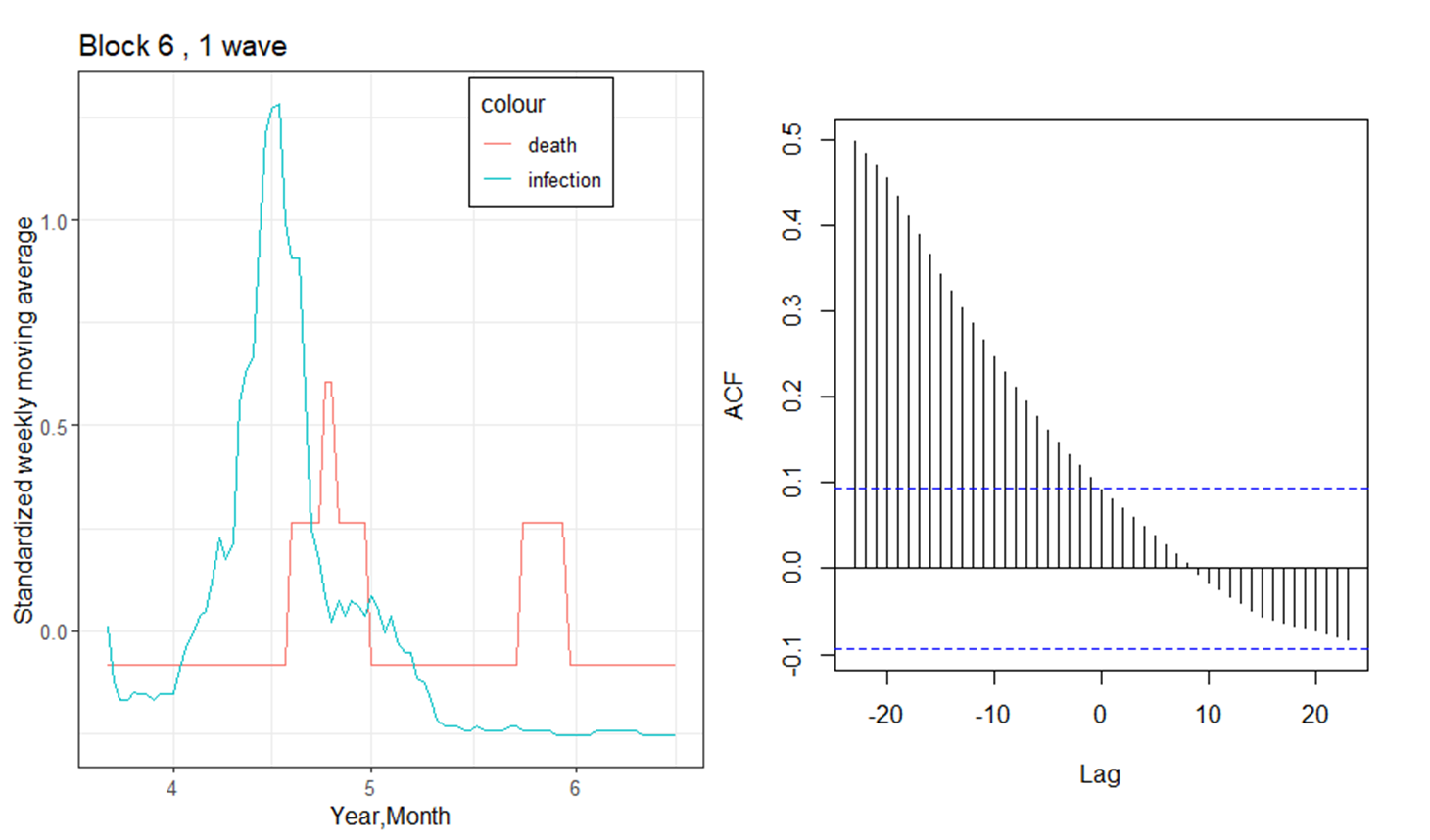


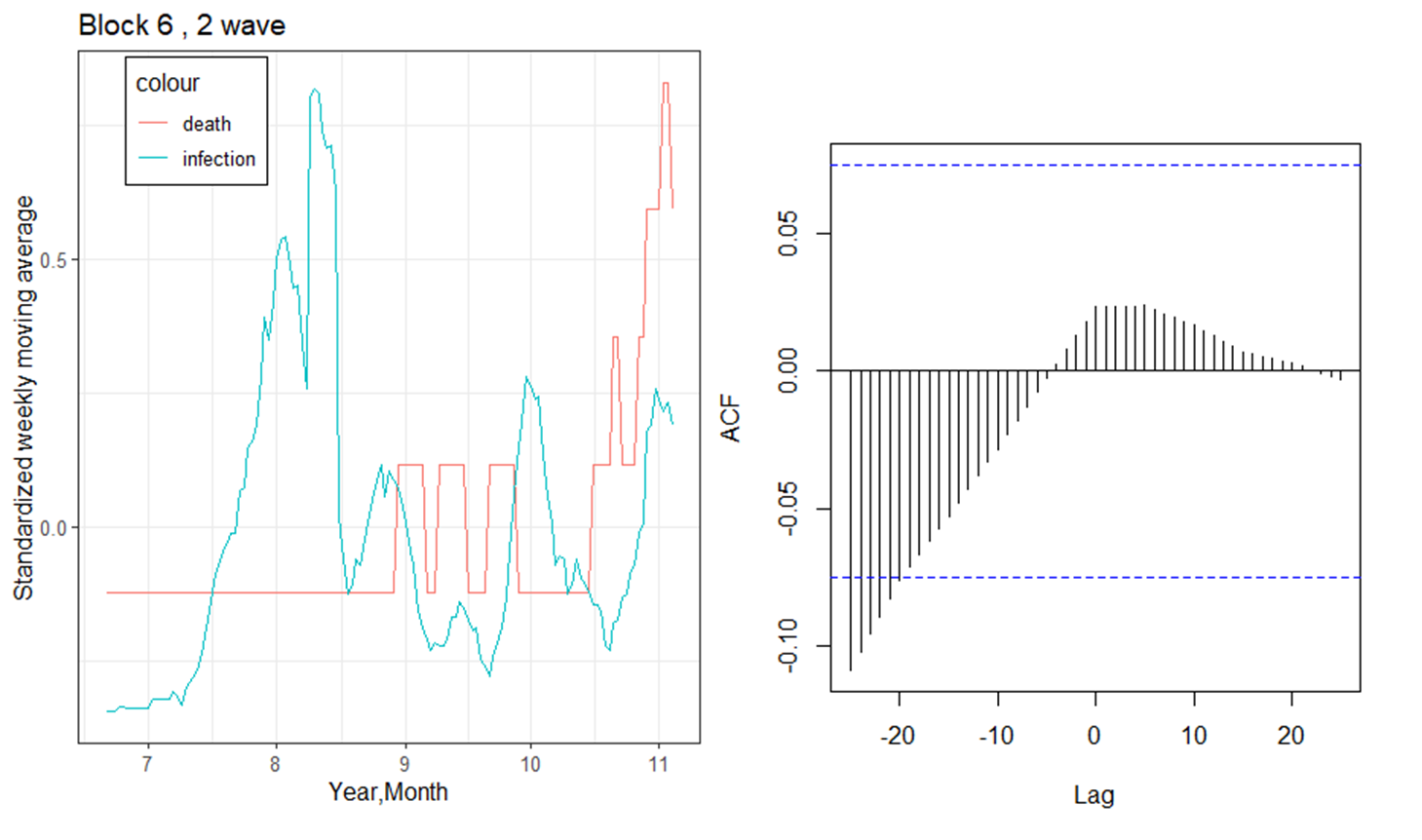


**
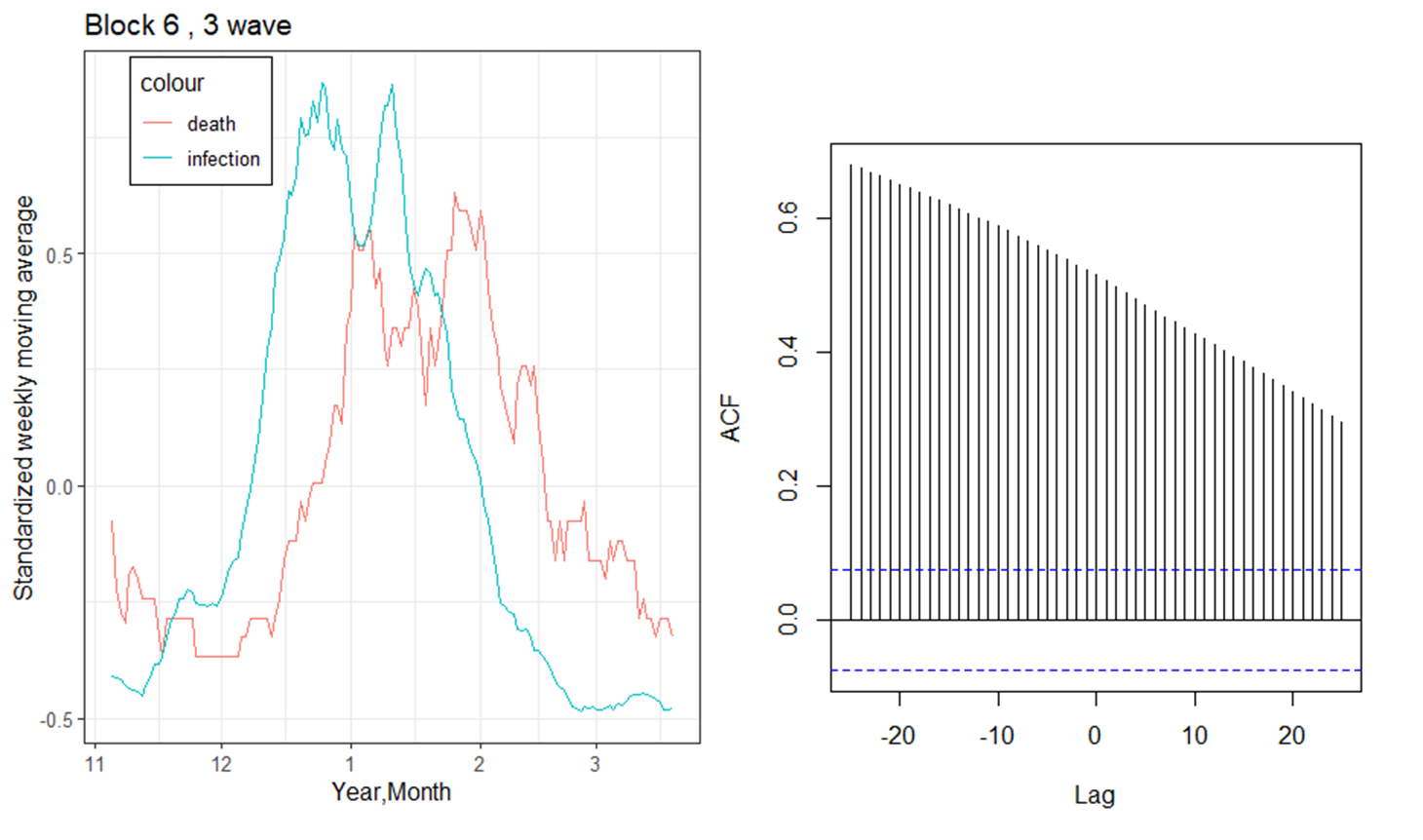
**

**
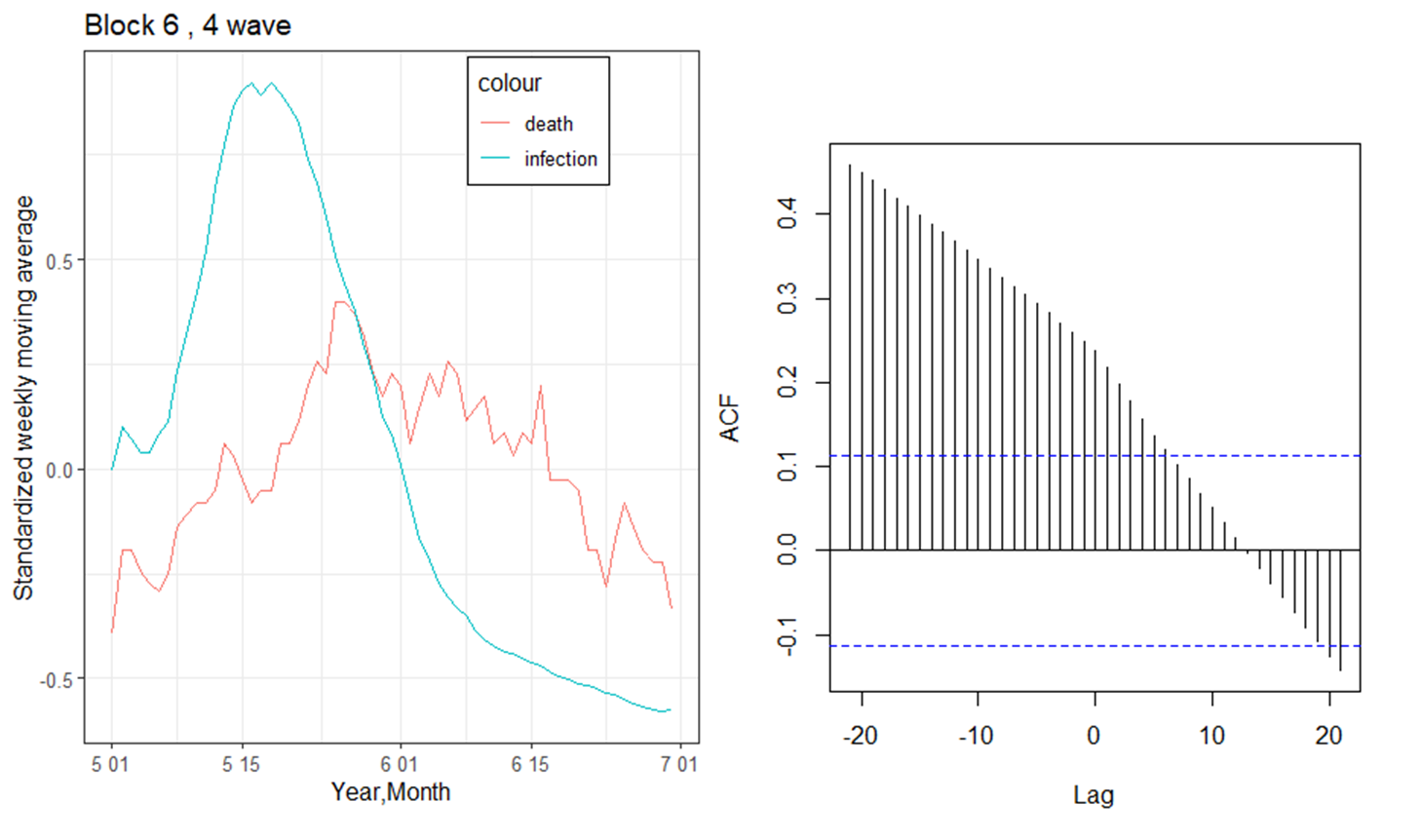
**

**
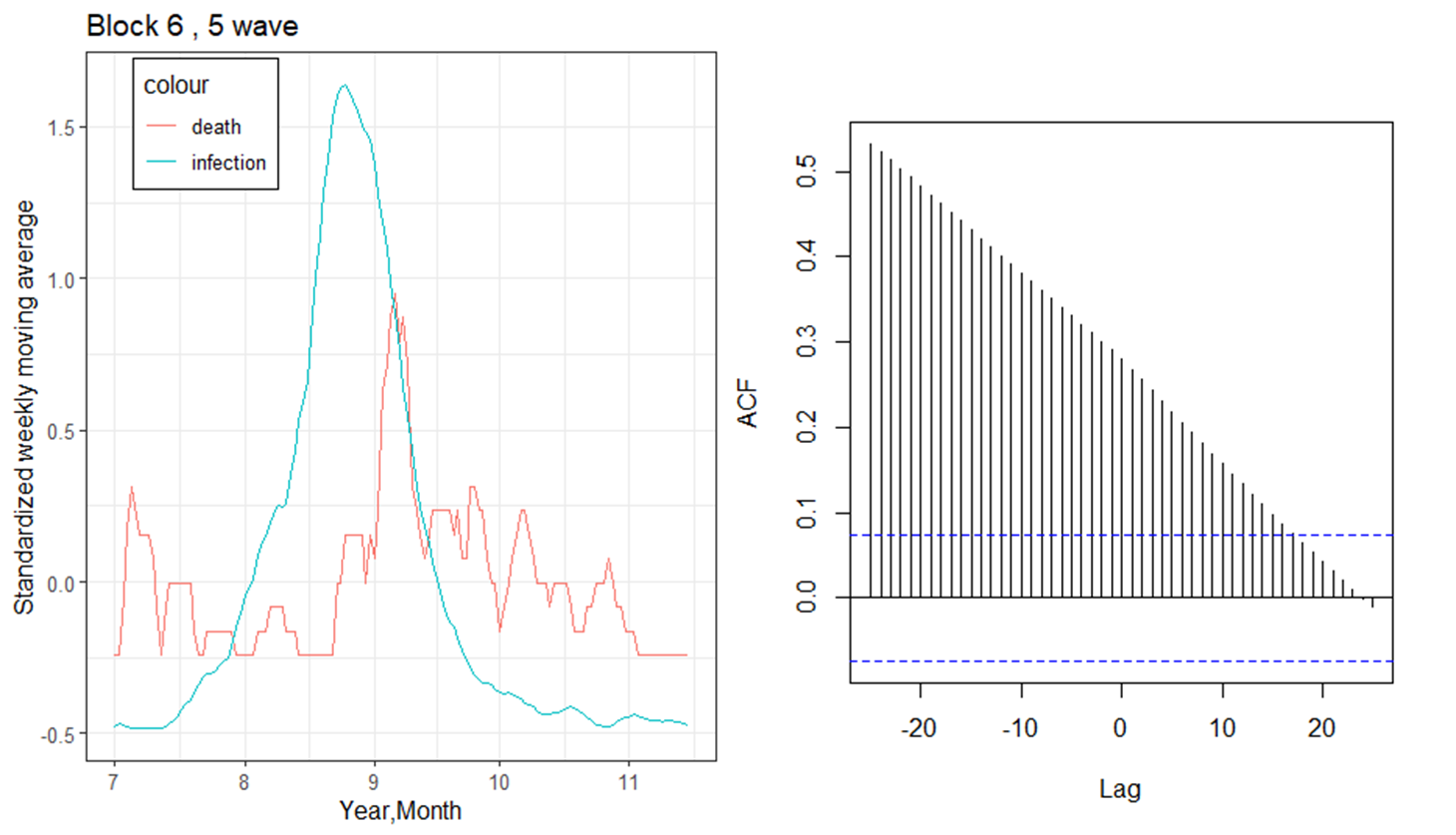
**

**
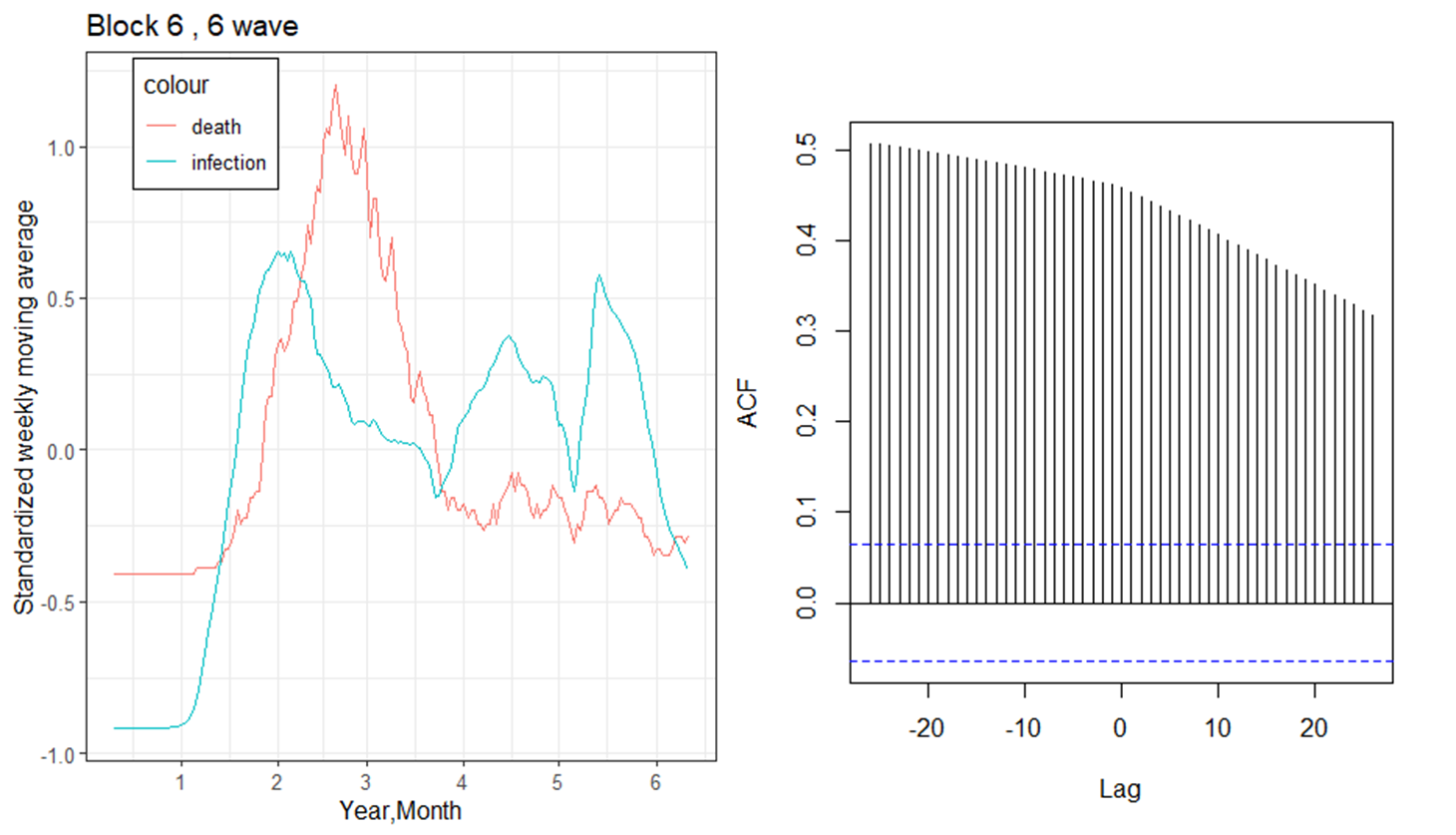
**

**
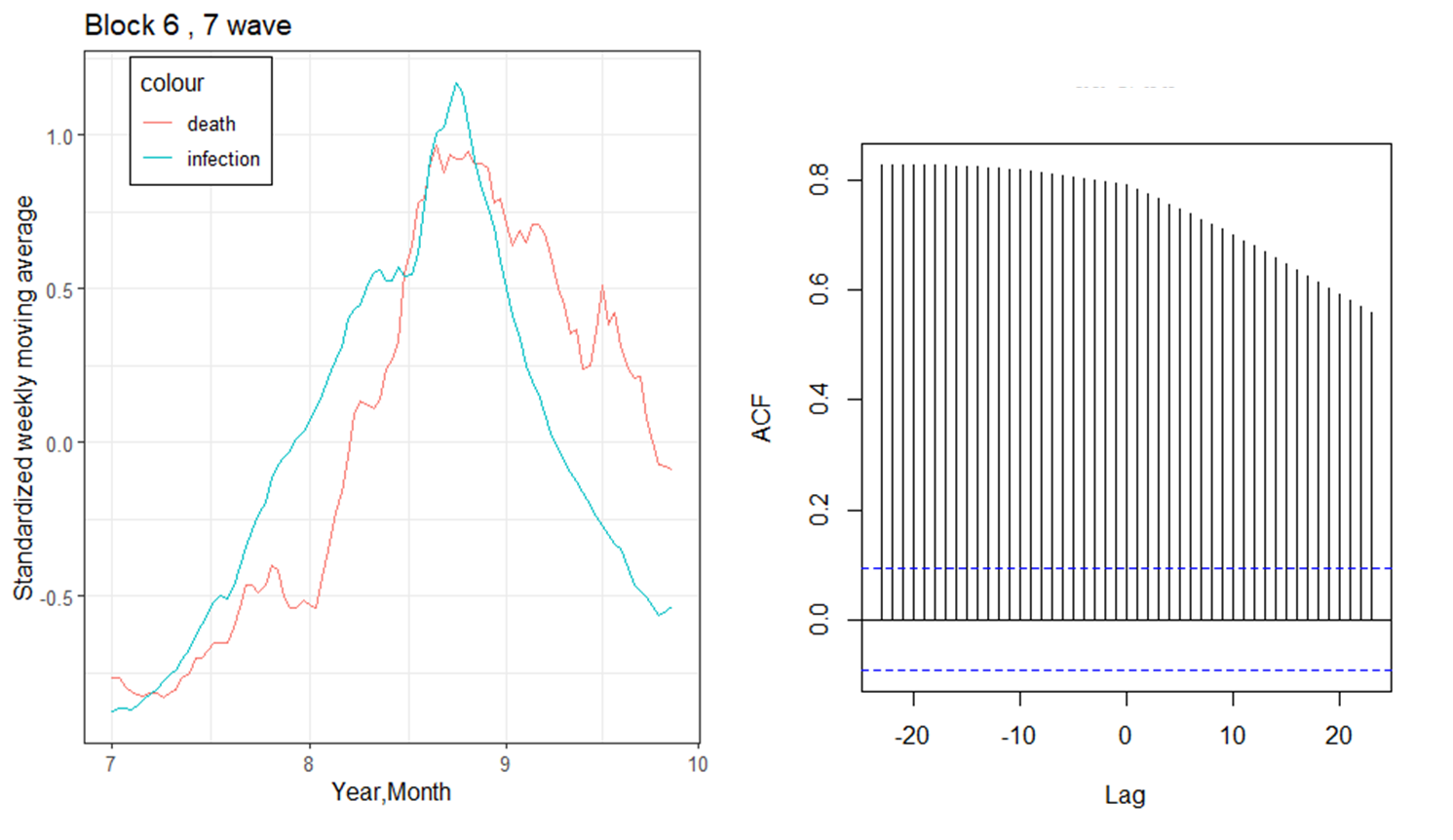
**

**(g) Shikoku block**


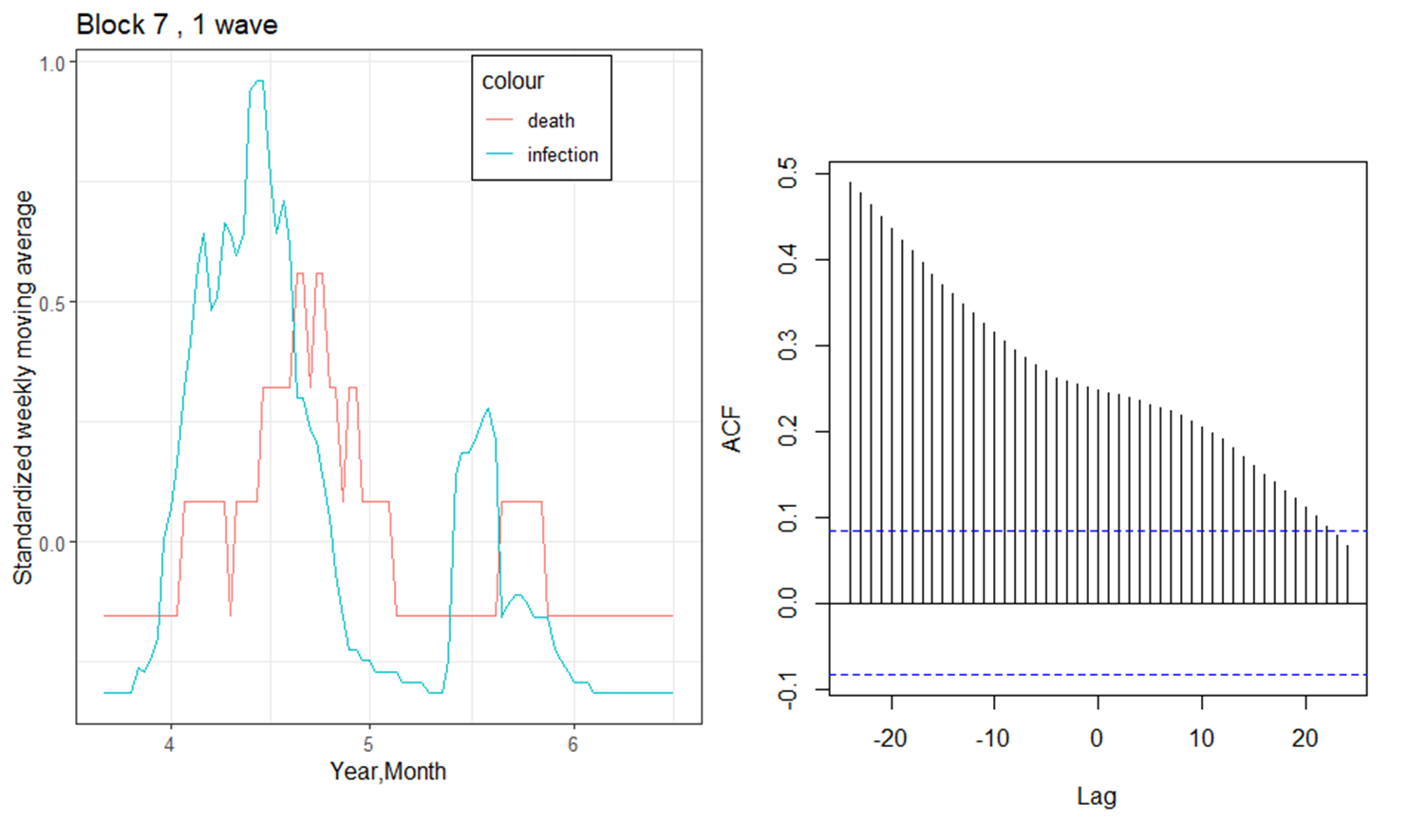


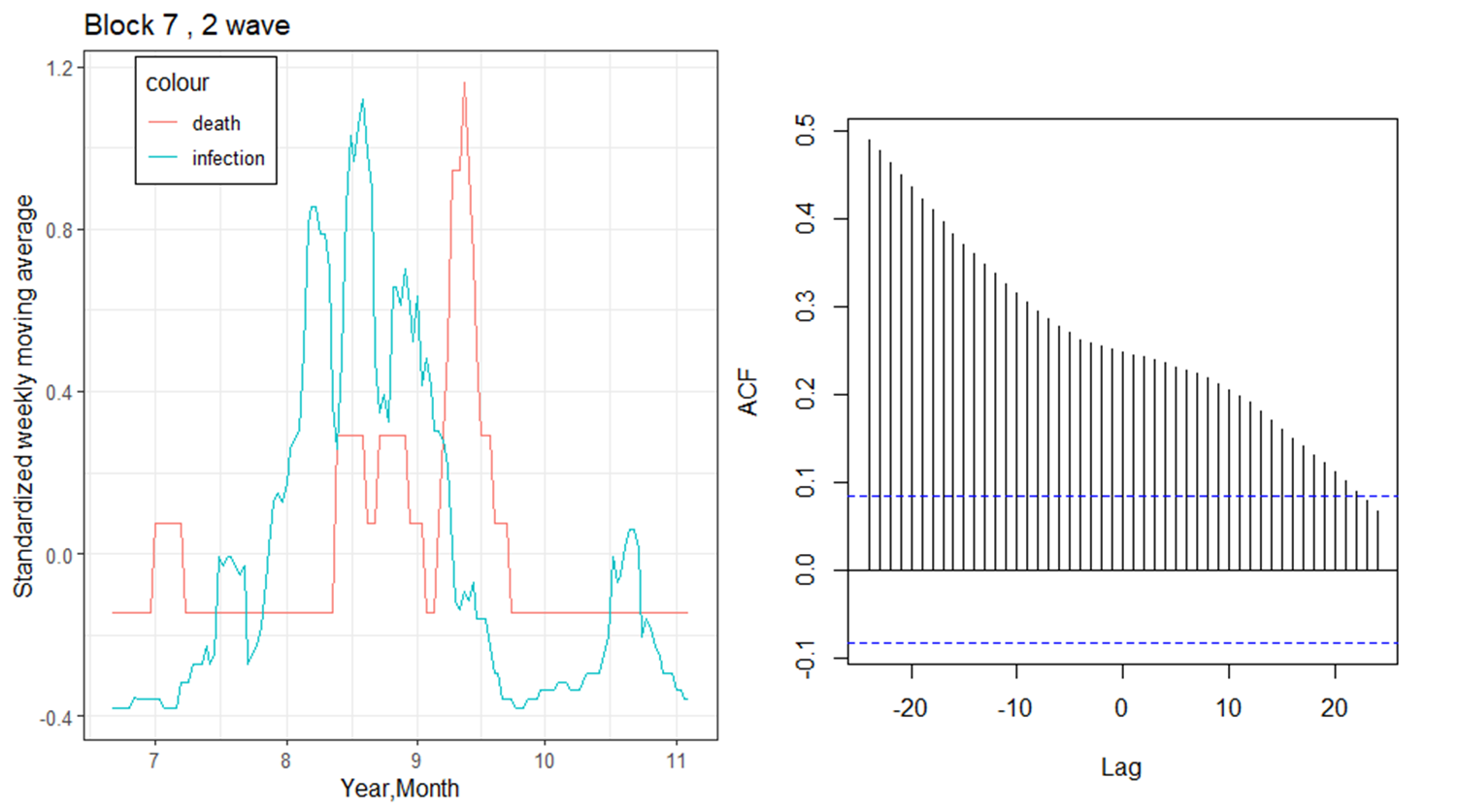


**
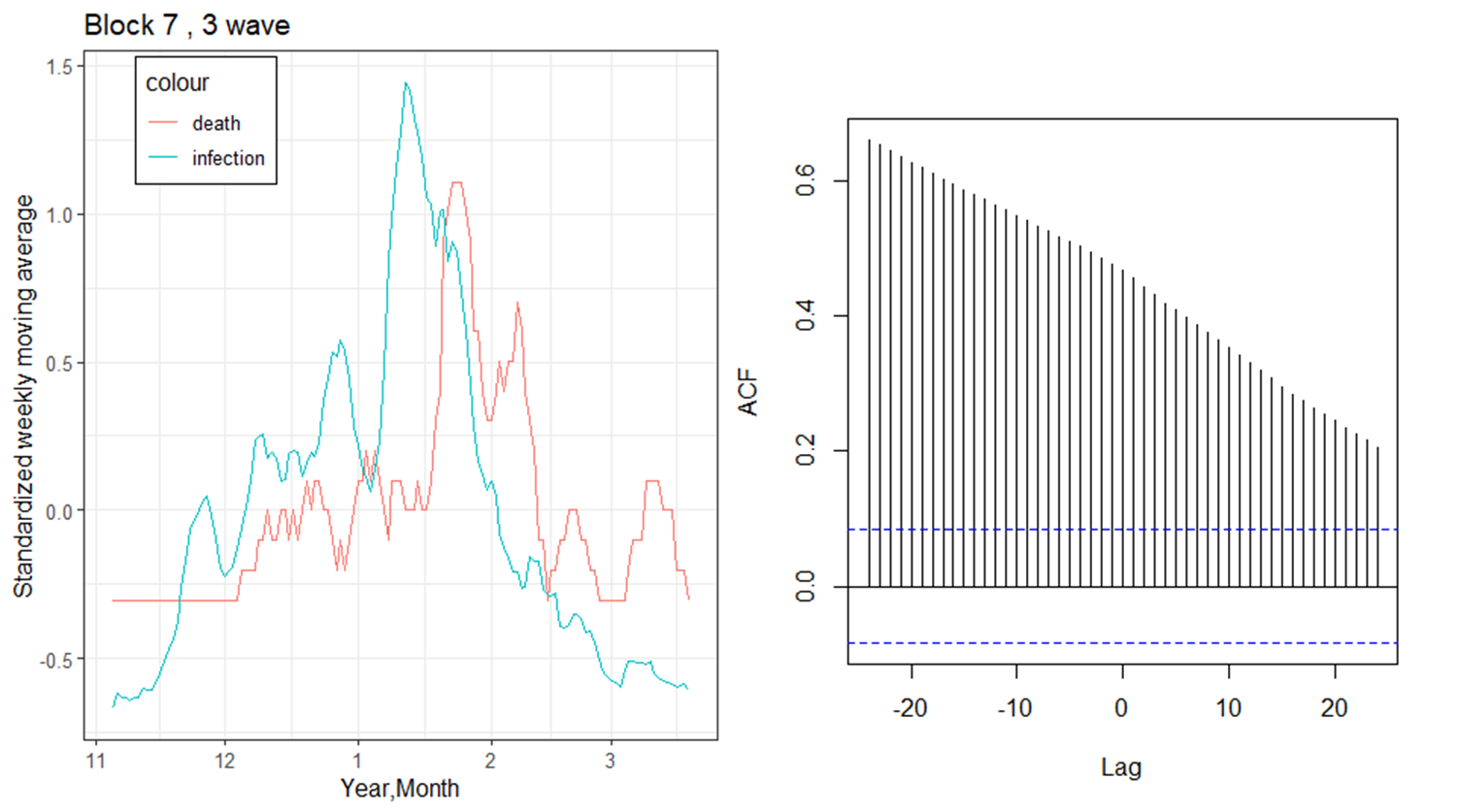
**

**
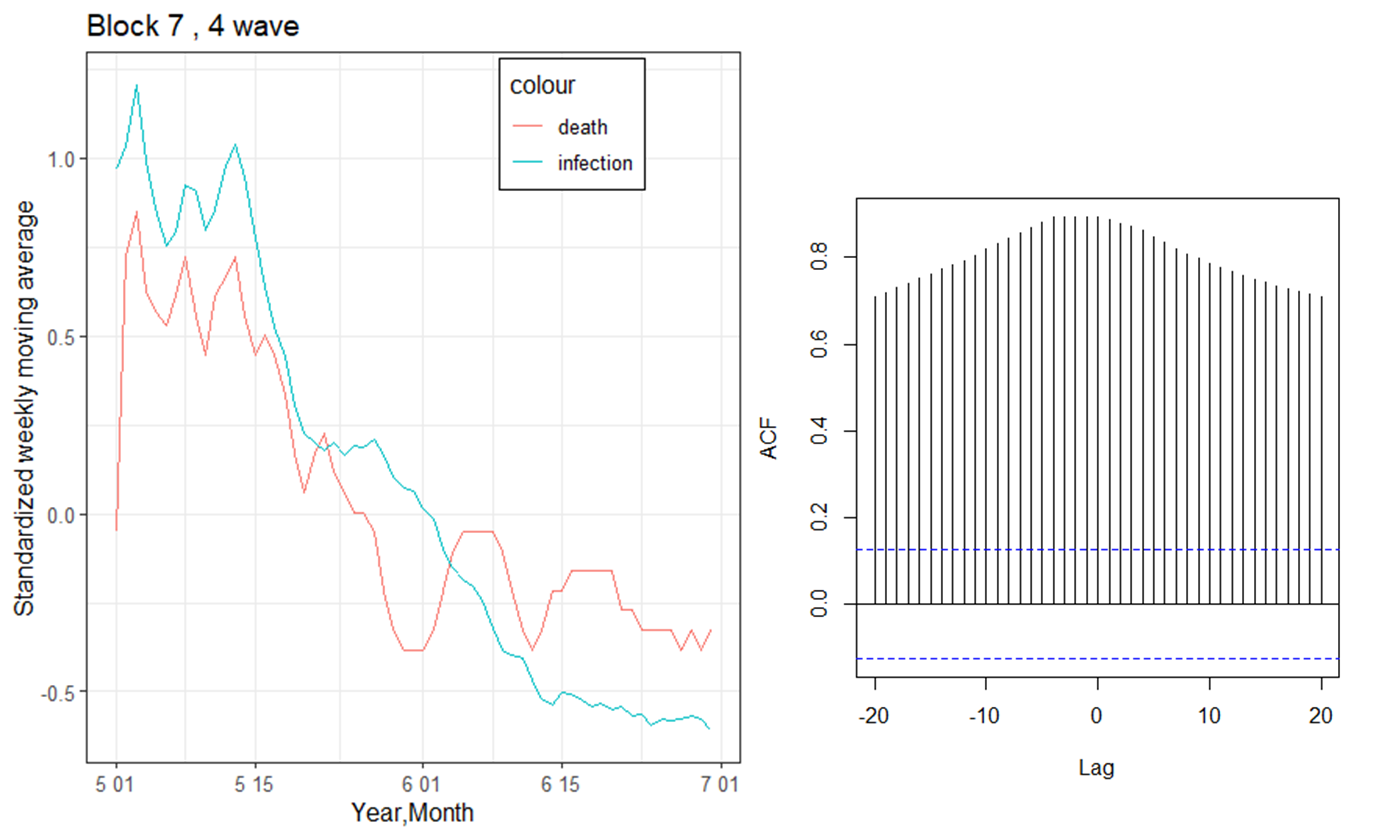
**

**
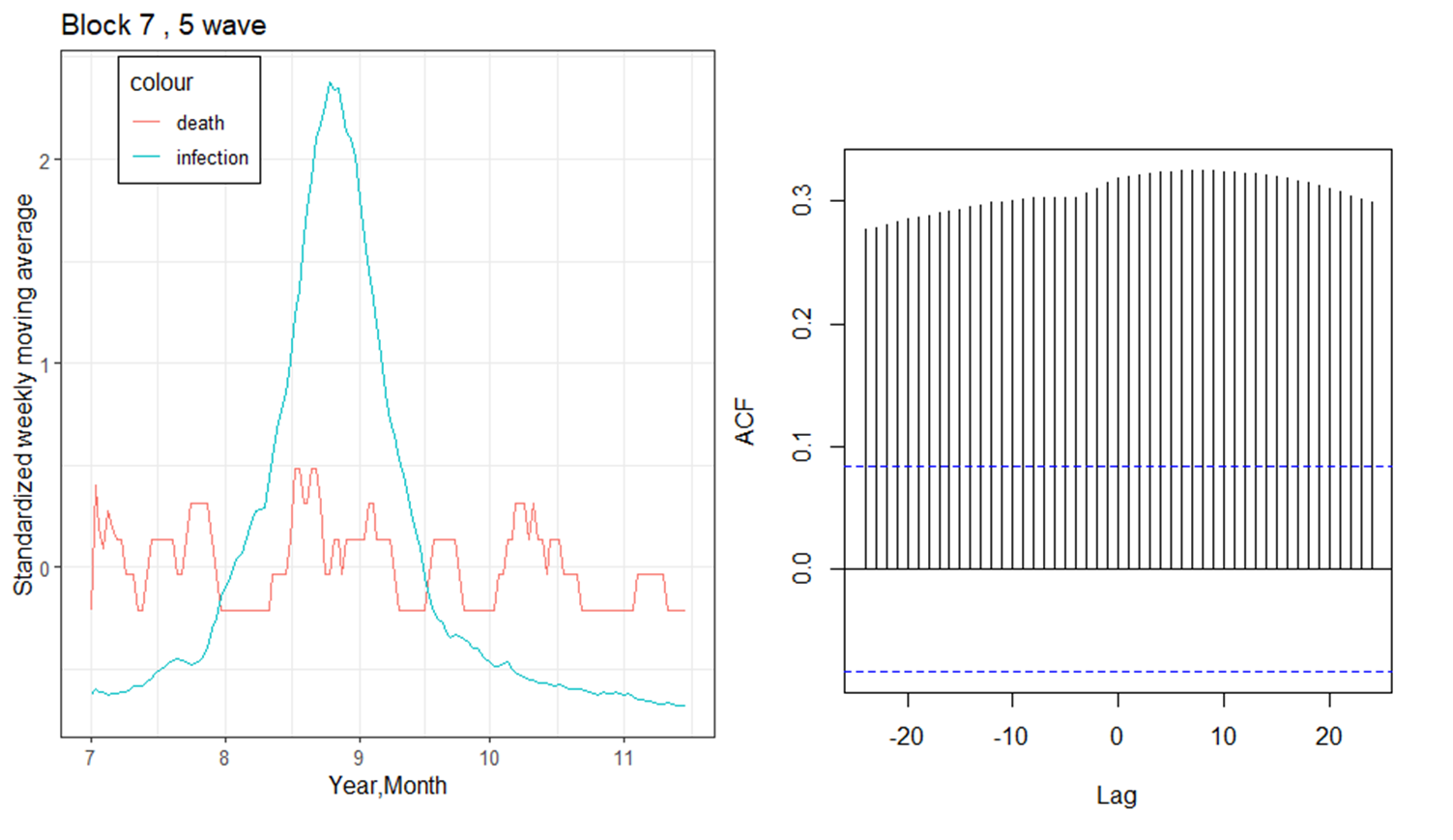
**

**
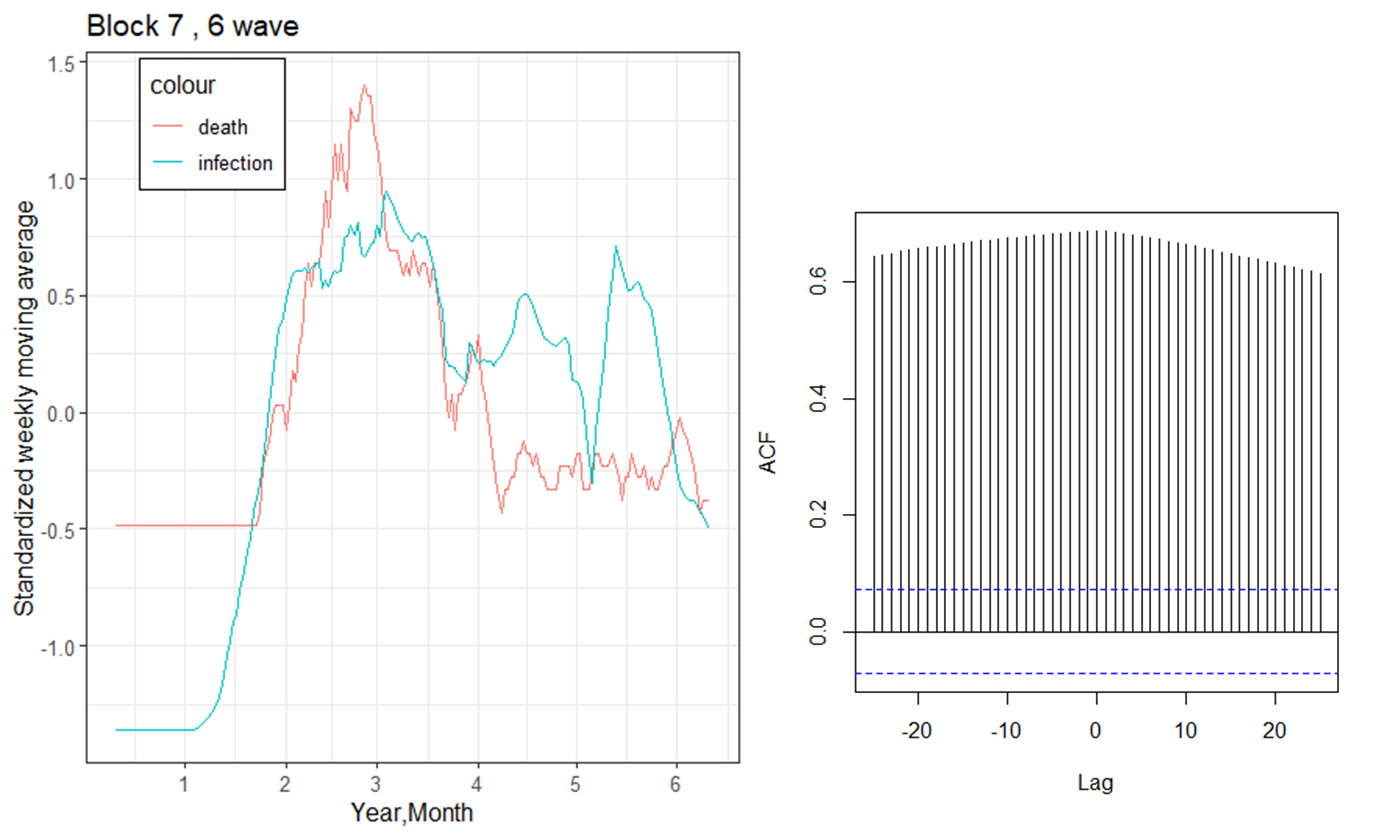
**

**
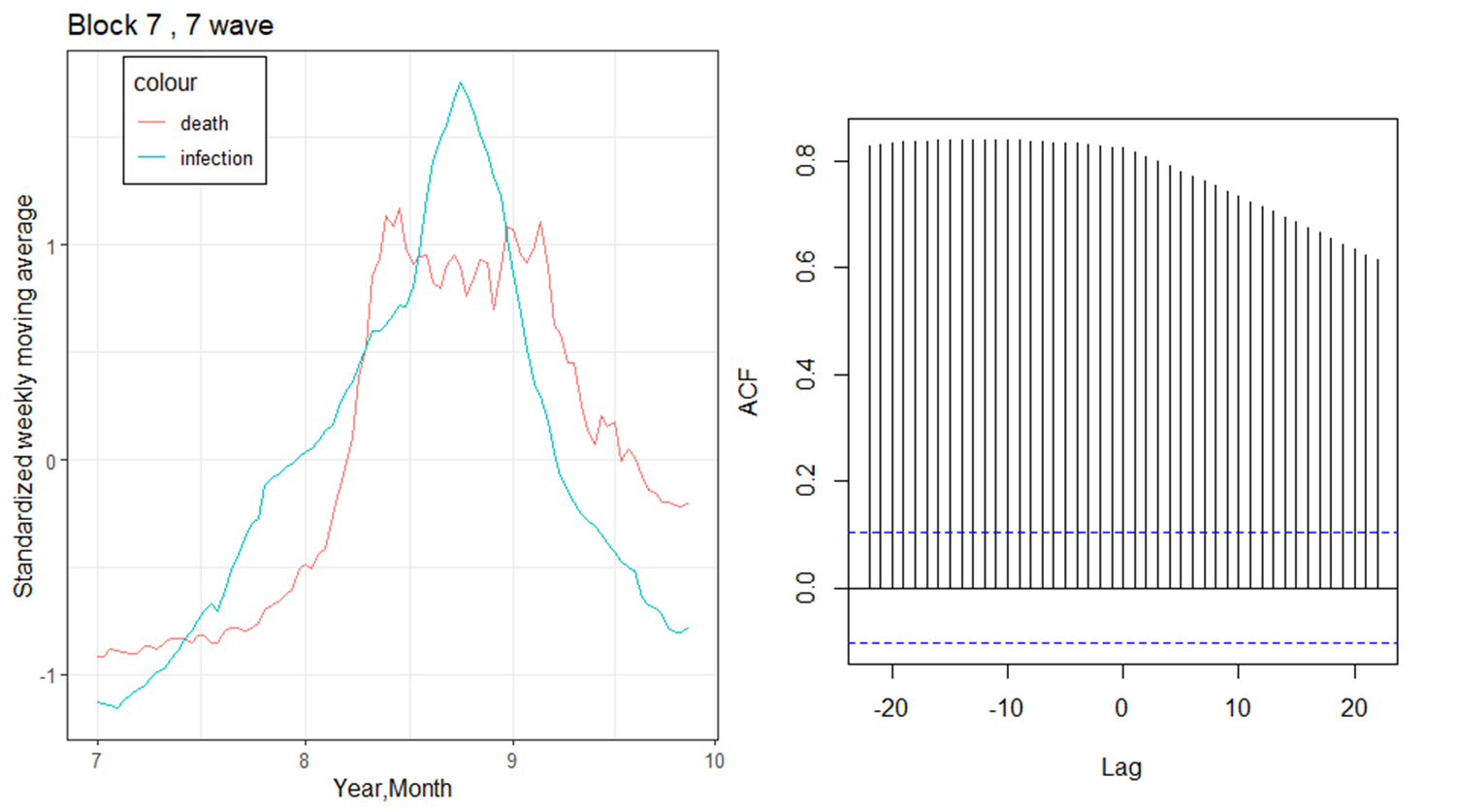
**

**（h）Kyushu**

**
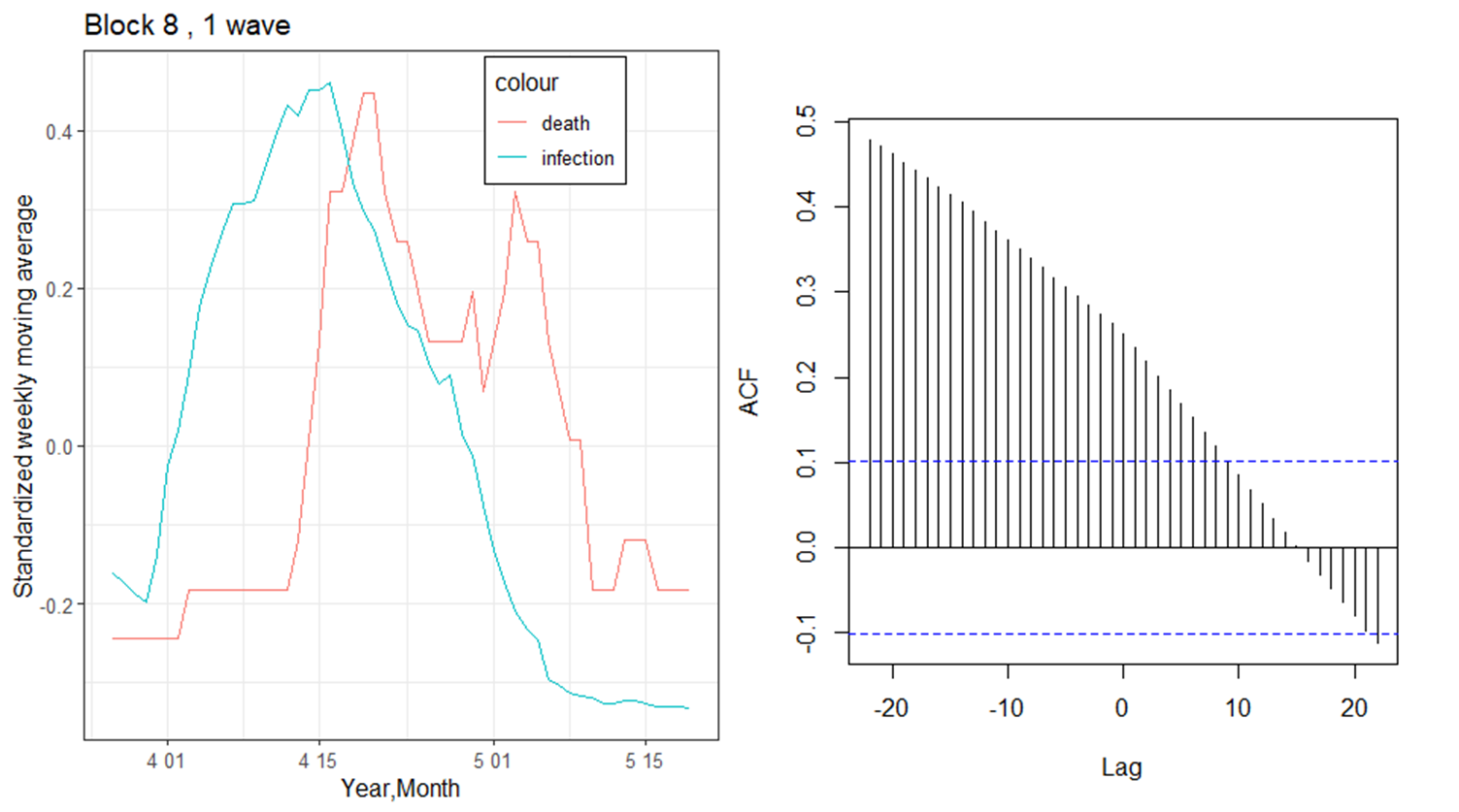
**

**
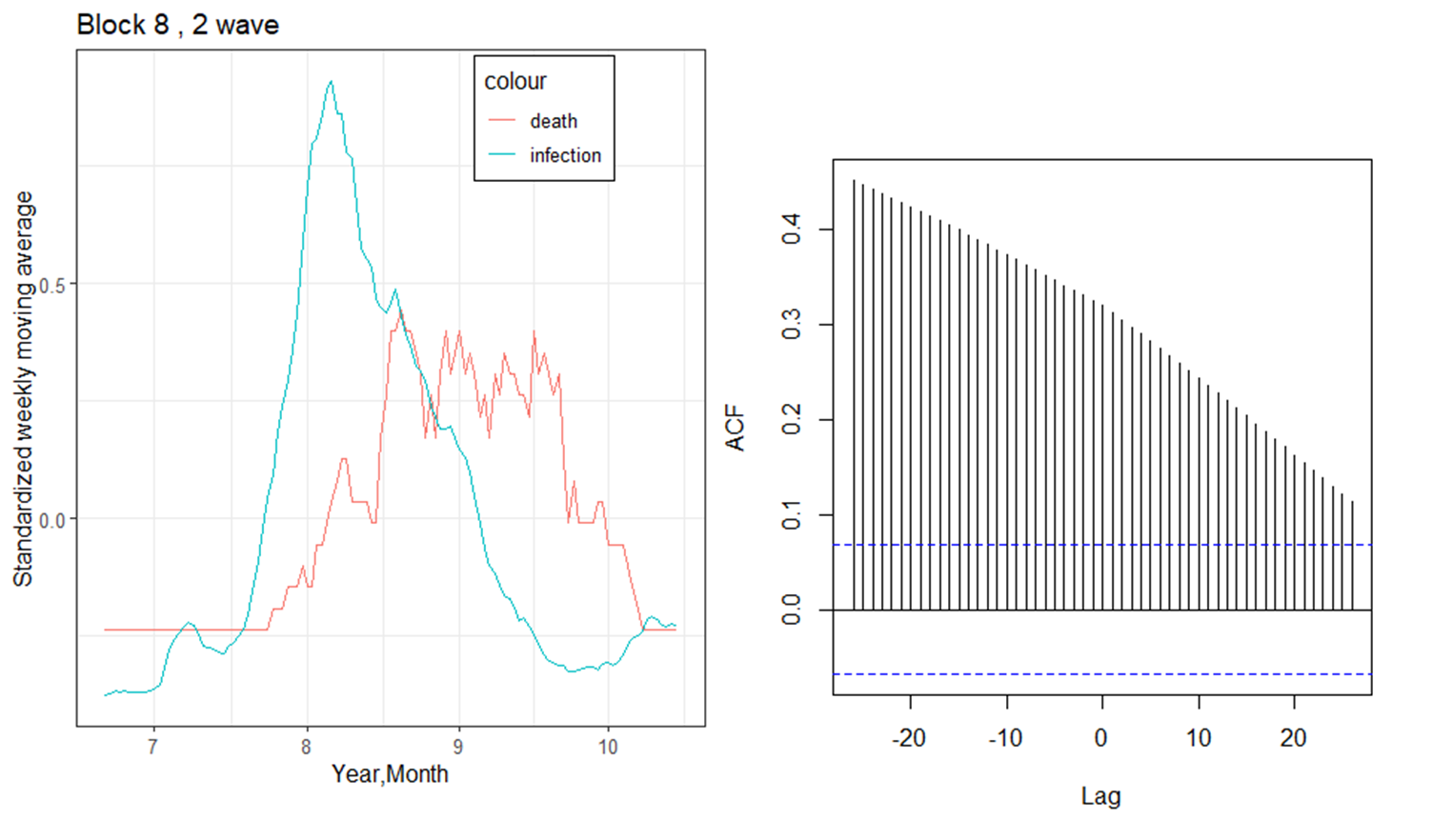
**

**
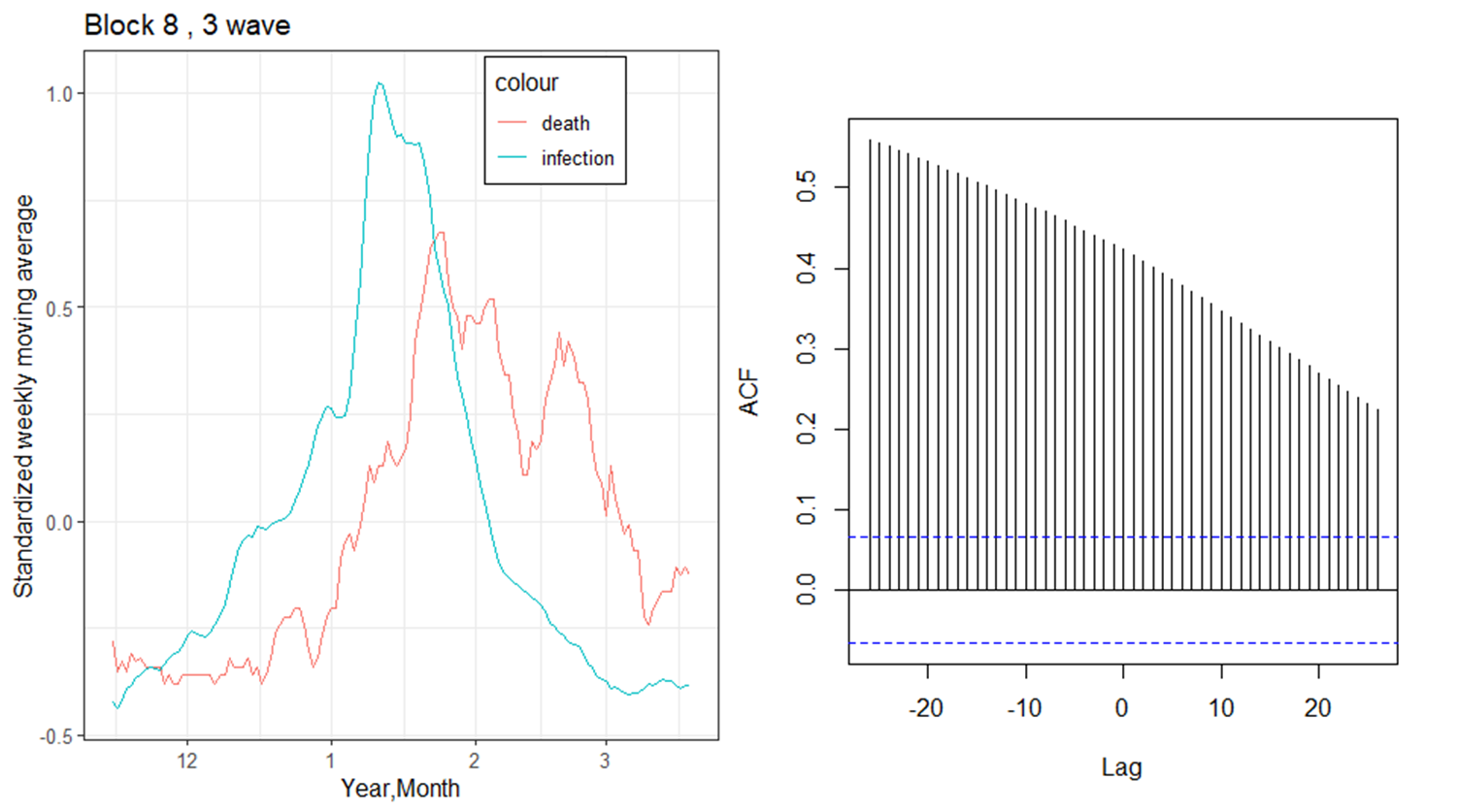
**

**
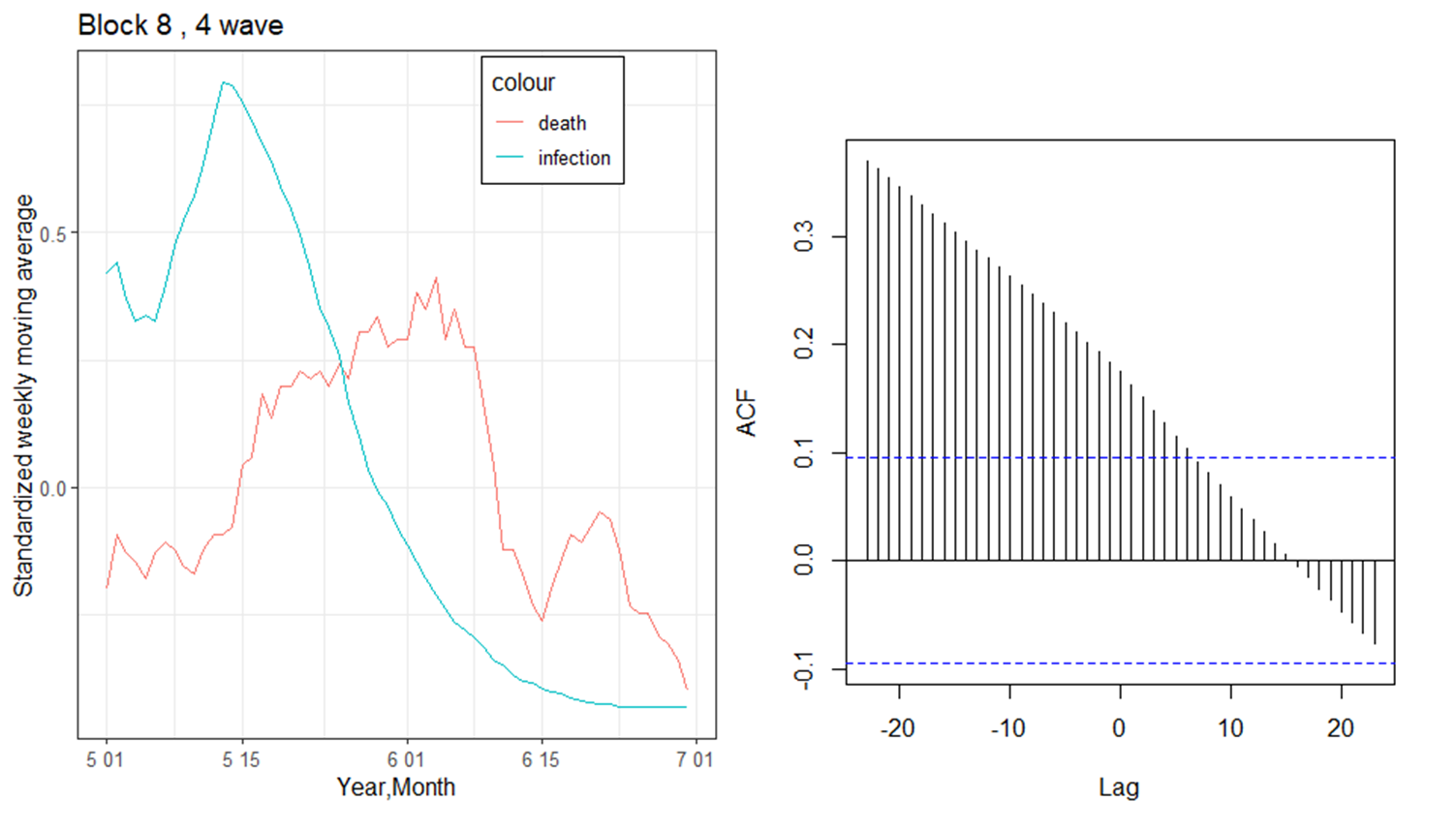
**

**
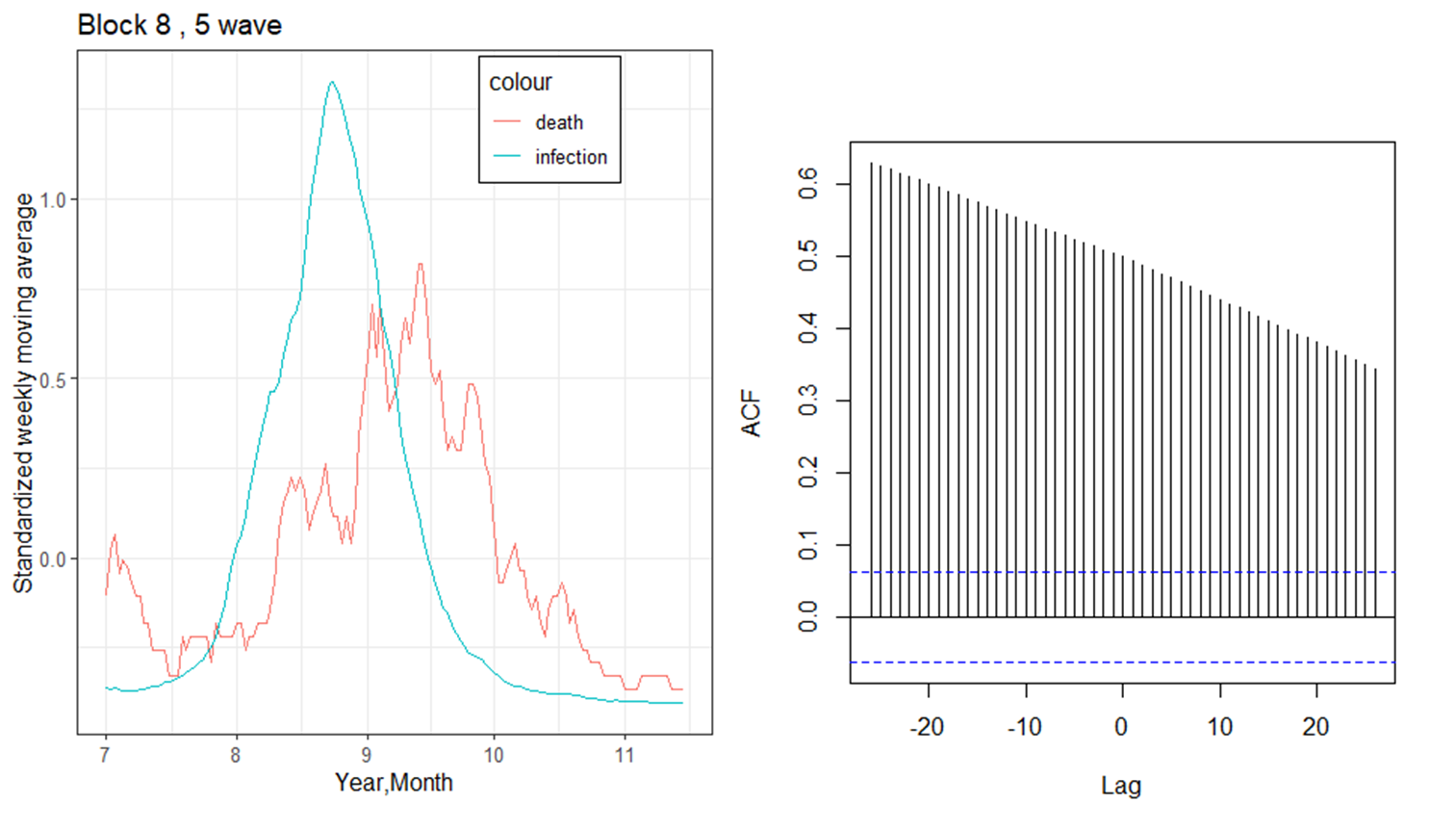
**

**
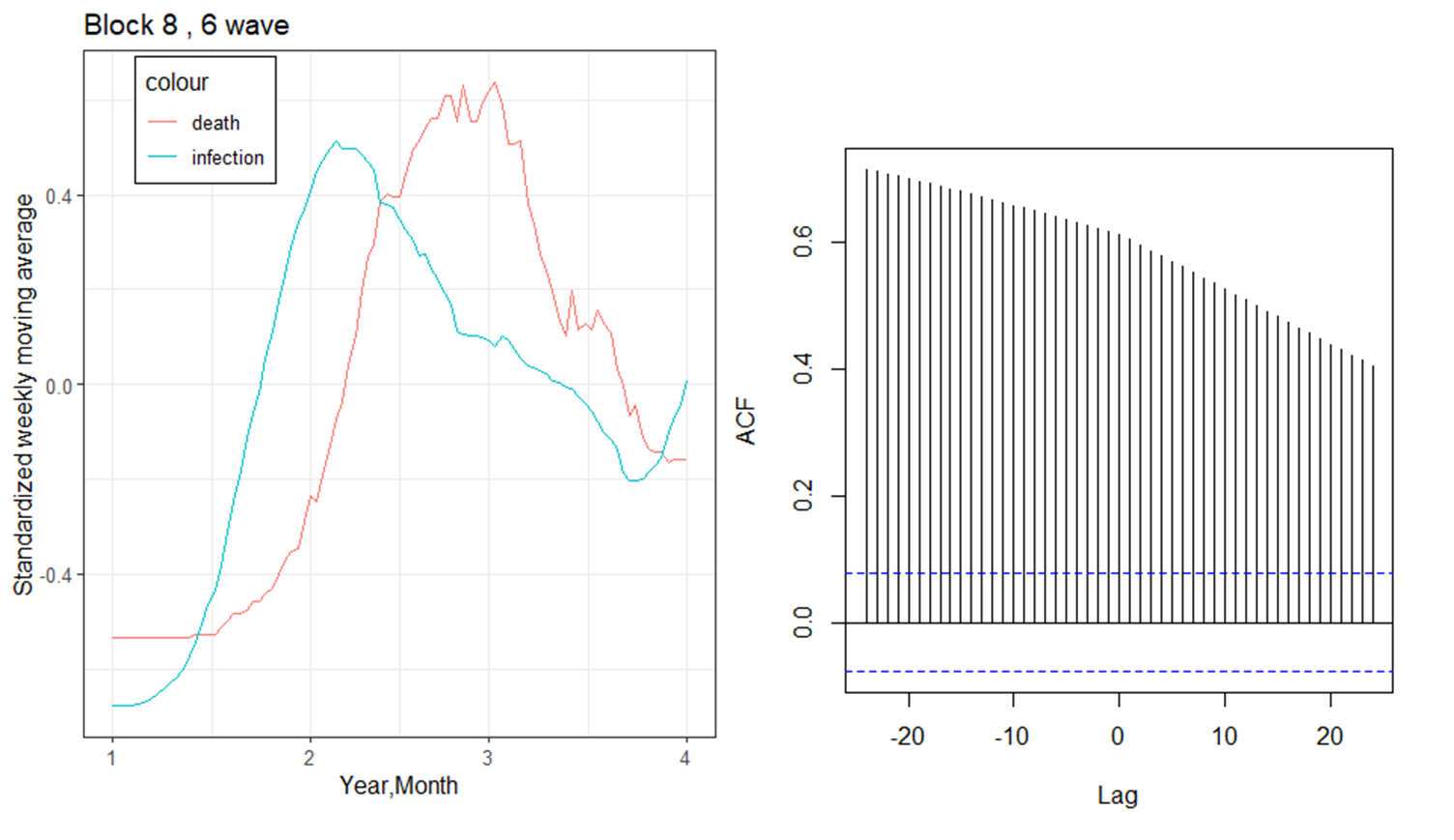
**

**
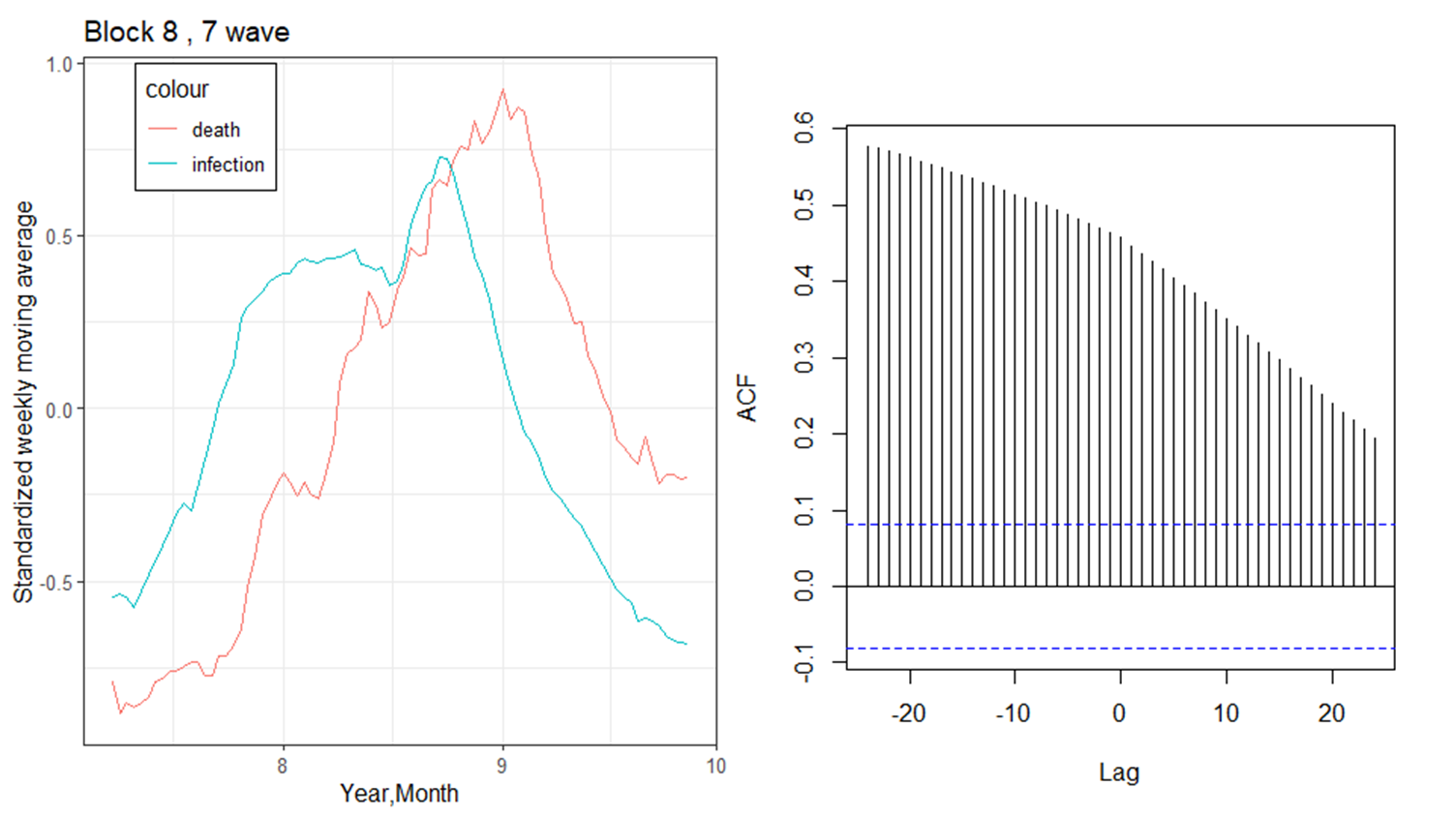
**

**(h) Okinawa block**

**
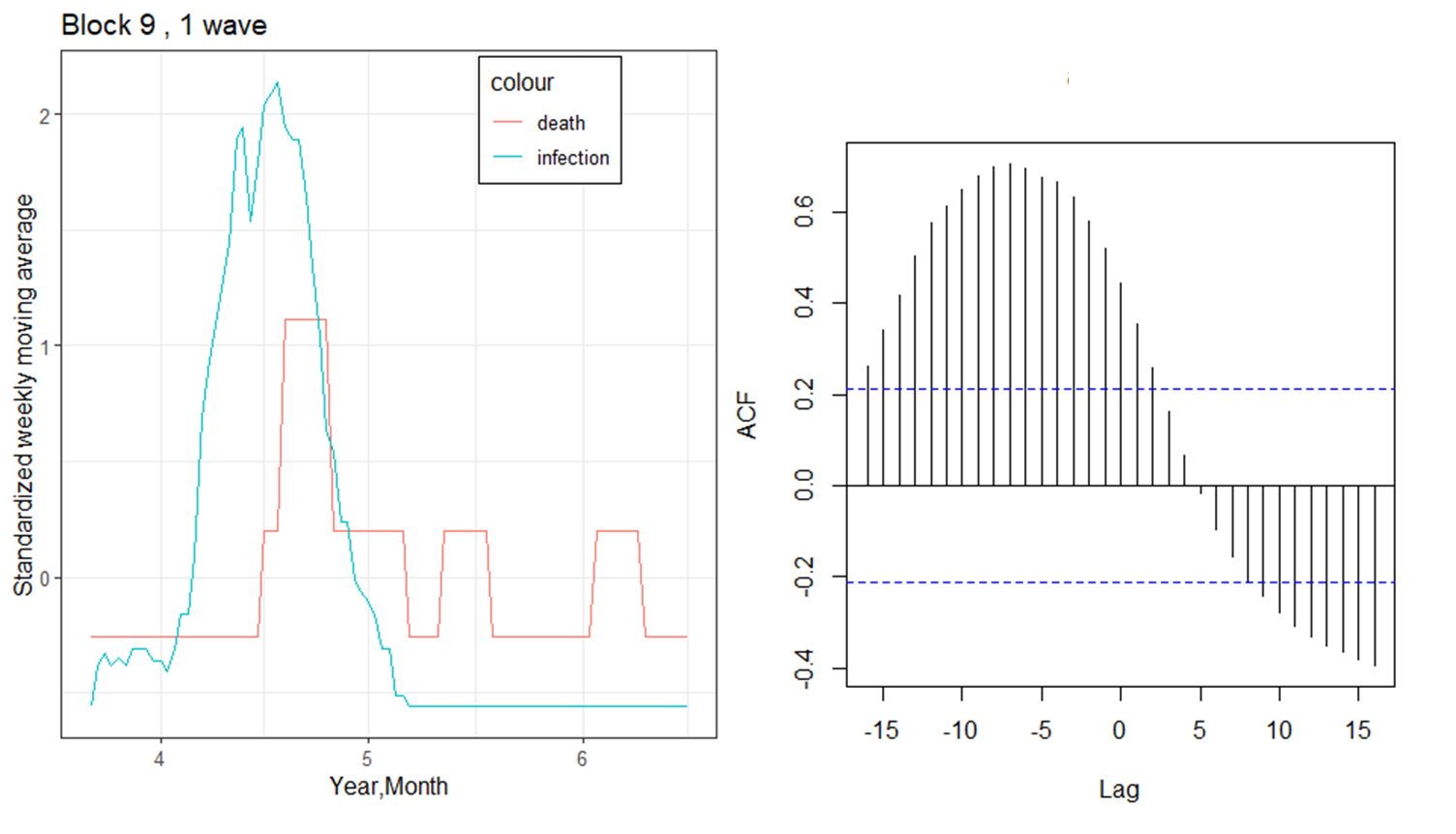
**

**
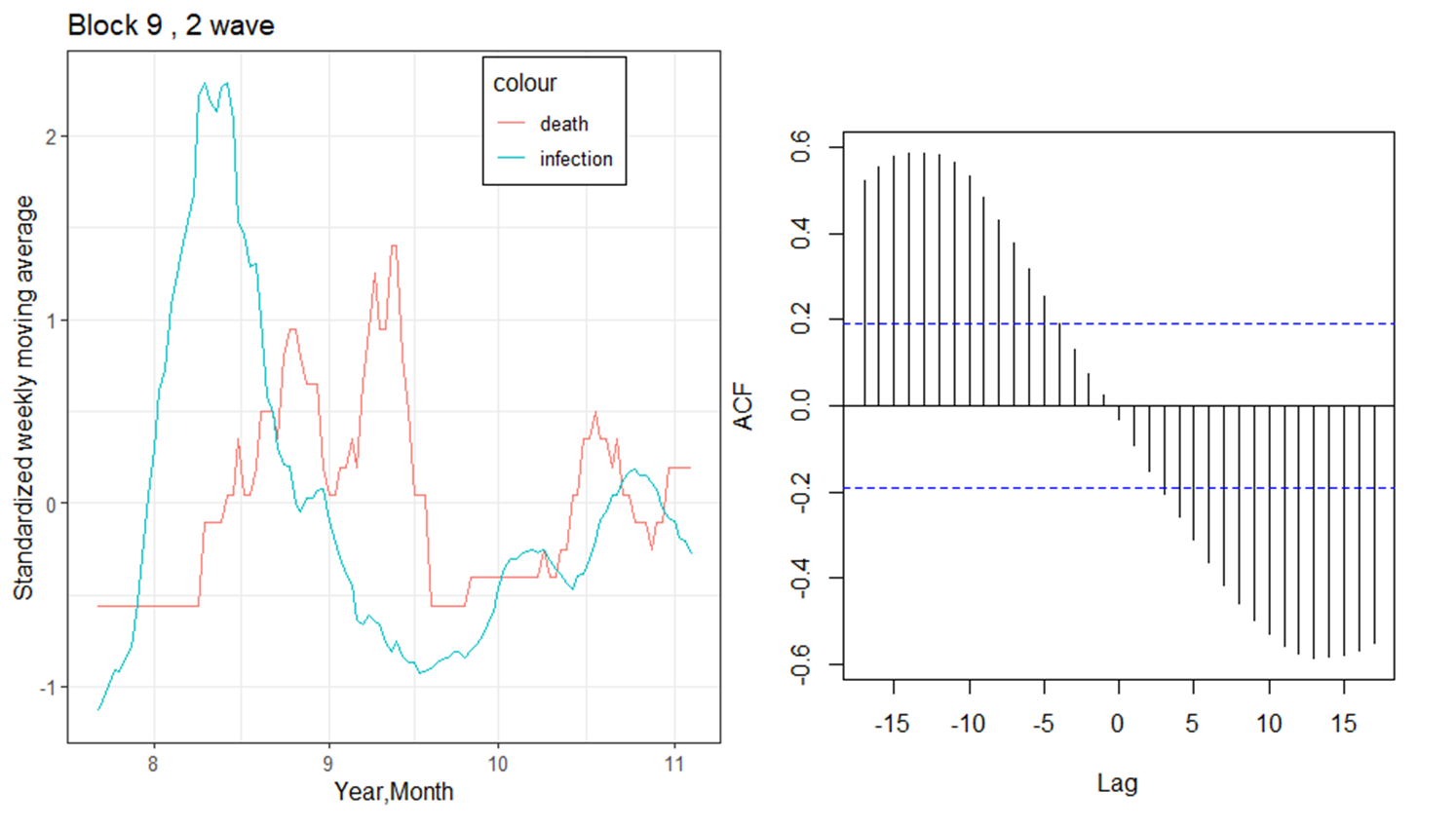
**

**
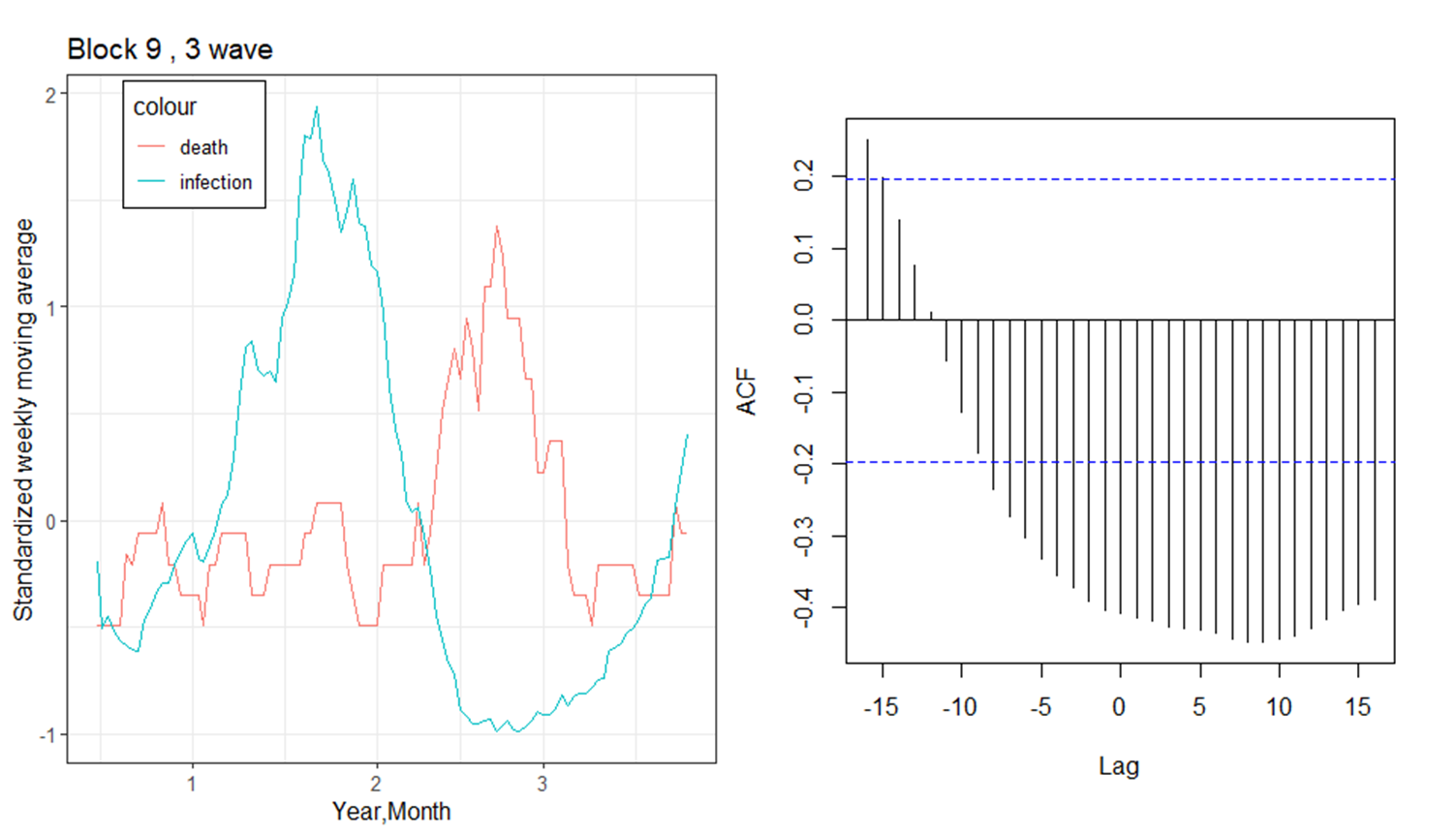
**

**
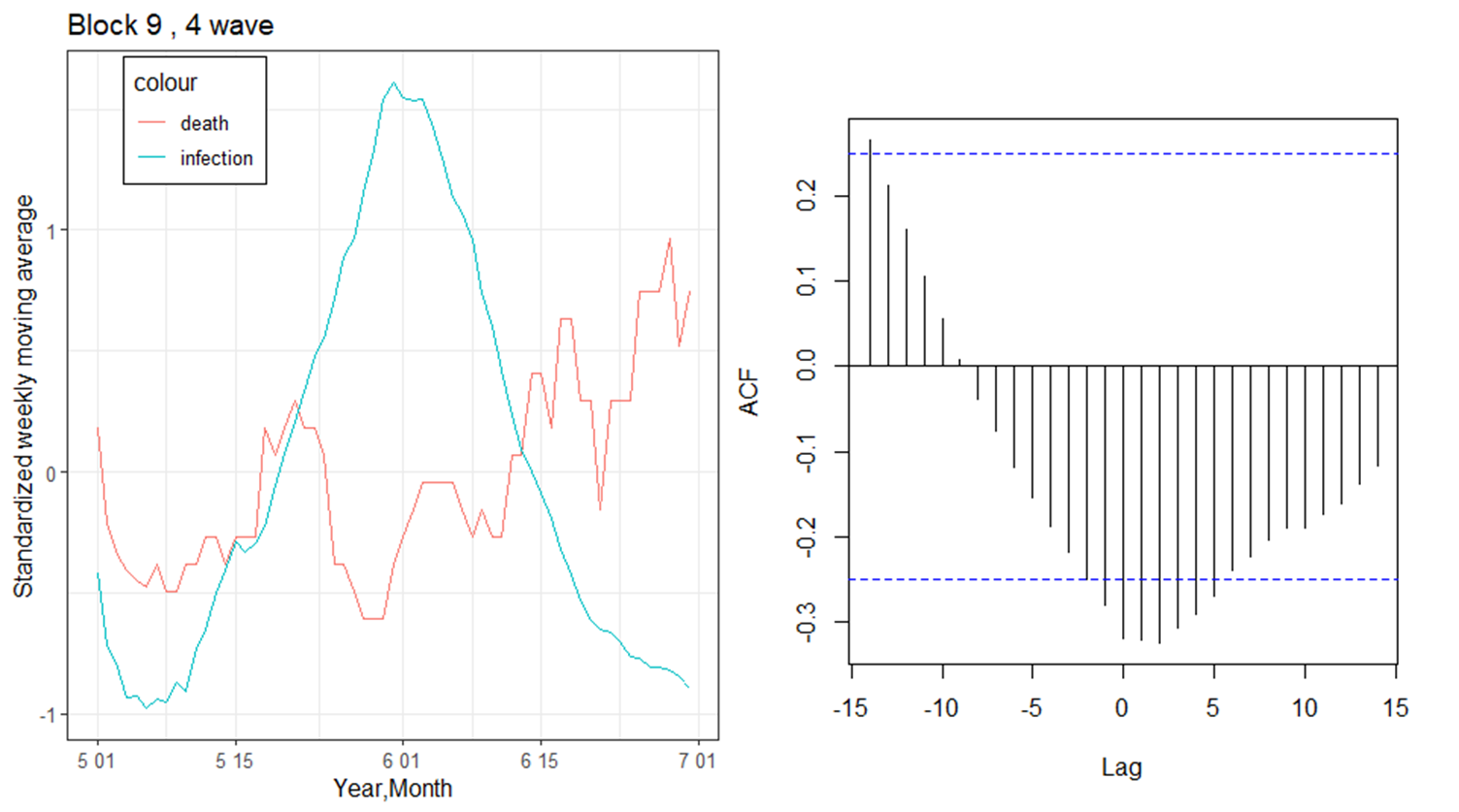
**

**
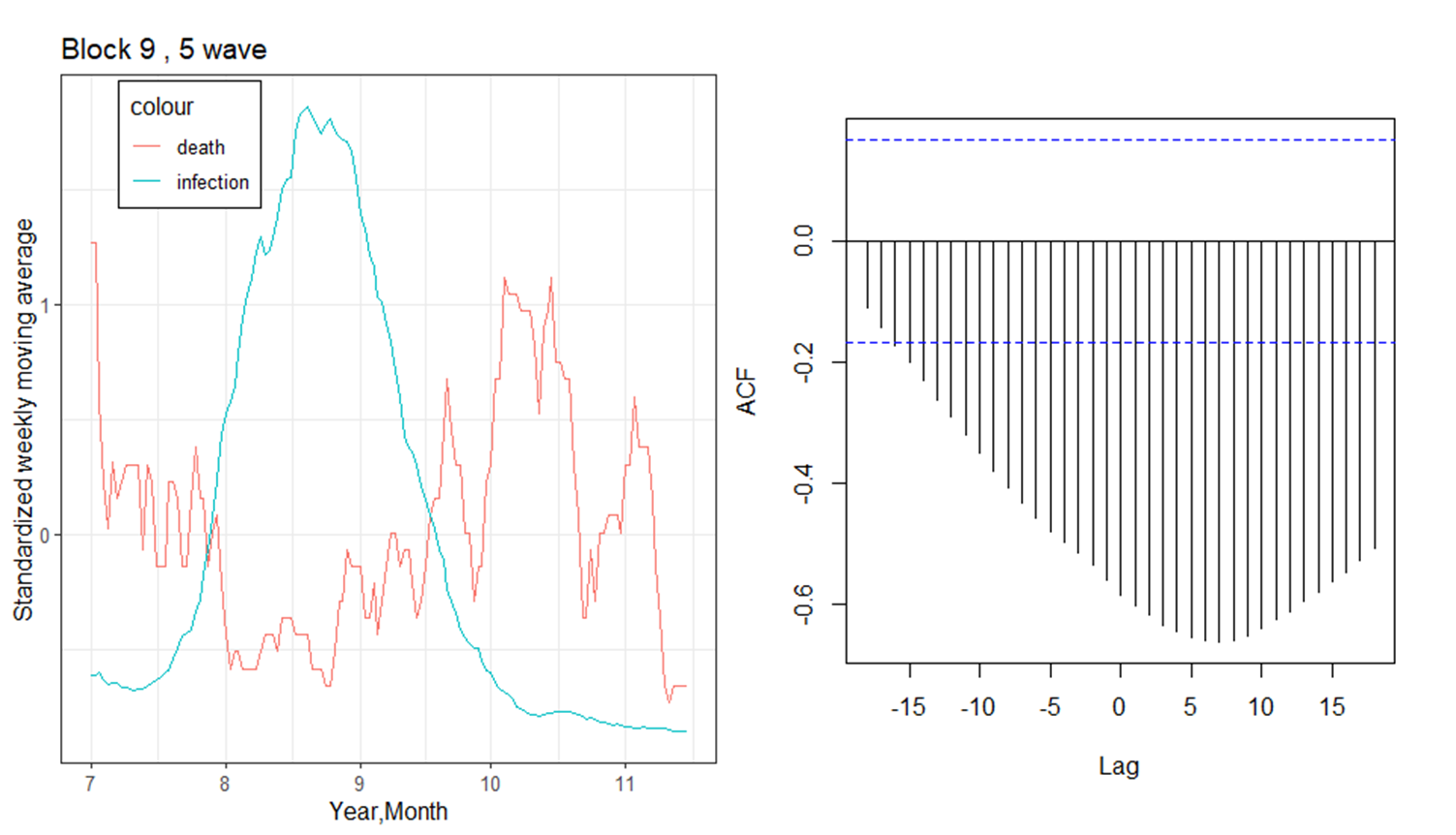
**

**
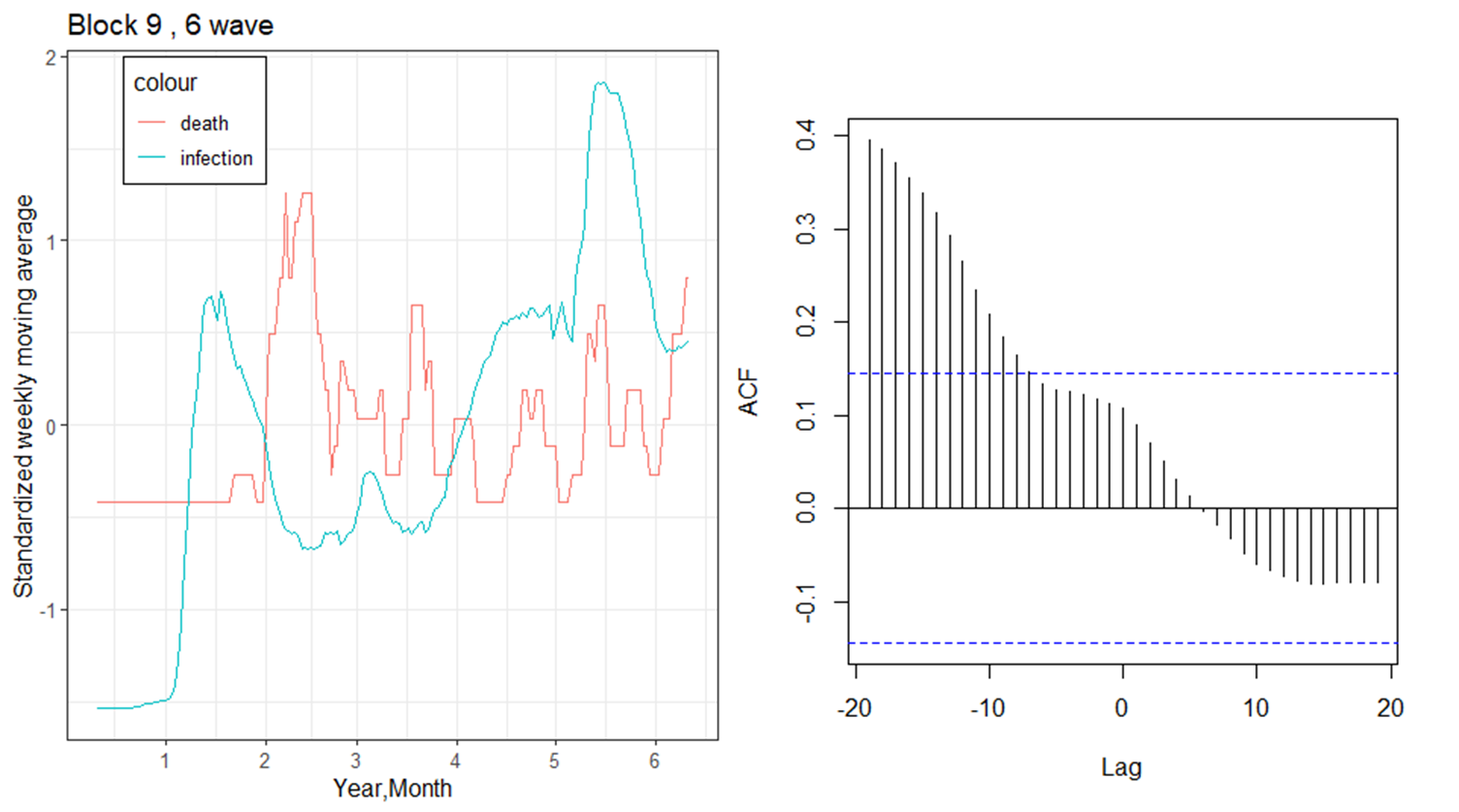
**

**
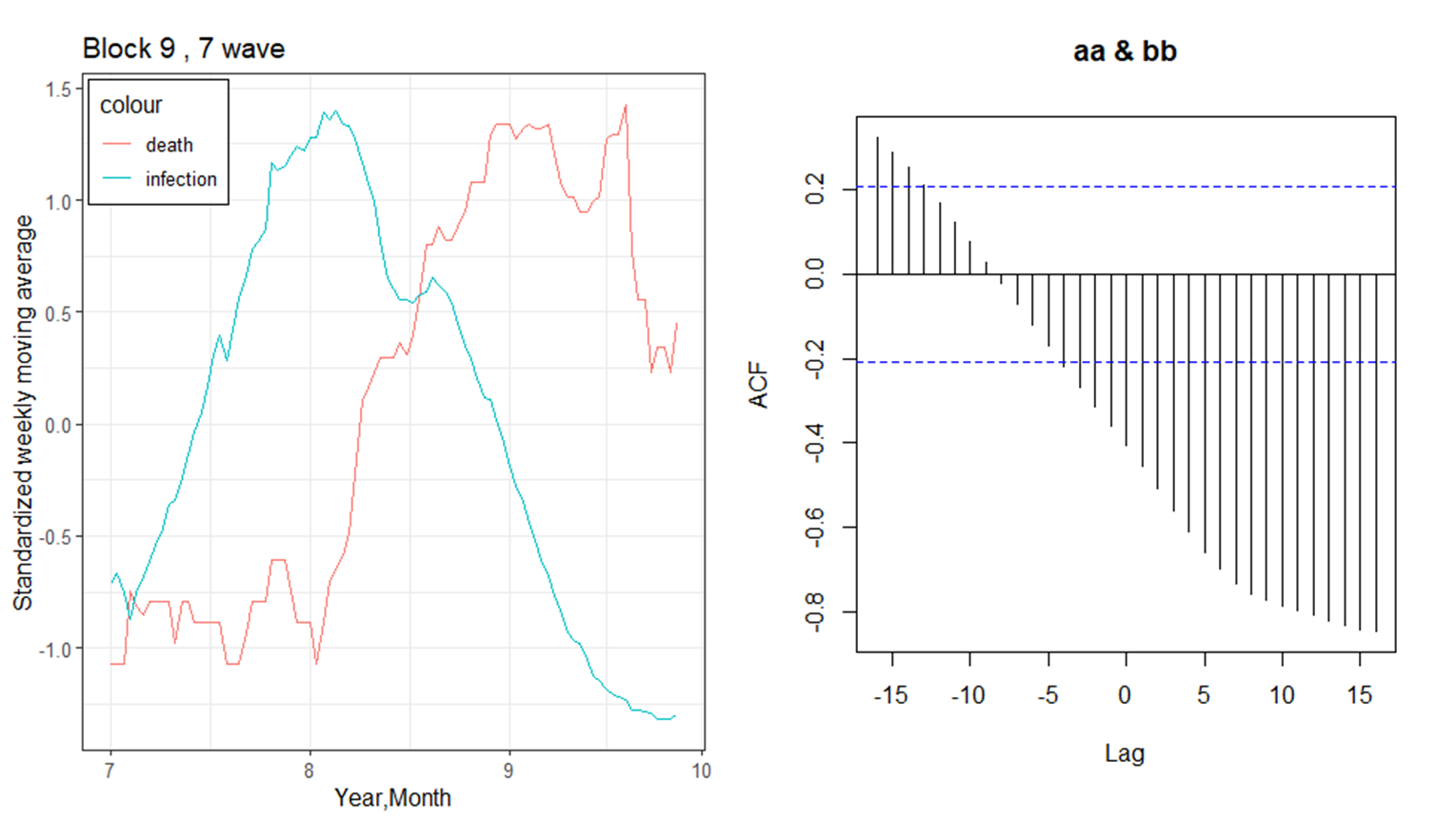
**
