# Supplementary material for: Case fatality rate considering the lag time from the onset of COVID-19 infection to related death from 2020 to 2022 in Japan
Source: IJID Reg. 2023 May 1;8:36–48. doi: 10.1016/j.ijregi.2023.04.013 (PMC10149354; doi:10.1016/j.ijregi.2023.04.013)
Supplement: Supplementary file 2 [file mmc2.docx]

Supplementary Table 1

Material	Provided by	Access site
Covid-19 infection number	Ministry of Health Labour and Welfare	https://www.mhlw.go.jp/stf/covid-19/kokunainohasseijoukyou.html
Vaccine coverage for elderly	Department of Medical Genome Sciences, Research Institute for Frontier Medicine, Sapporo Medical University School of Medicine	https://web.sapmed.ac.jp/canmol/coronavirus/japan_vaccine.html?a=1&age=65&d=0
Demographic data collection (2022)	IPSS	https://www.ipss.go.jp/syoushika/tohkei/Popular/P_Detail2022.asp?fname=T12-22.htm
Prefectural Gross Domestic Product (production side, nominal) *Same for expenditure side (Excel format: 31KB)		https://www.esri.cao.go.jp/jp/sna/data/data_list/kenmin/files/contents/main_2019.html
Aging Population (Section 1.4)		https://www8.cao.go.jp/kourei/whitepaper/w-2019/html/zenbun/s1_1_4.html
Special site for Covid-19	NHK	https://www3.nhk.or.jp/news/special/coronavirus/data-all/
Coronavirus Pandemic (COVID-19)	OurWorldInData.org	https://ourworldindata.org/coronavirus
